# Elemental composition and material properties of radular teeth in the heterobranch snail *Gastropteron rubrum* (Mollusca, Gastropoda, Cephalaspidea) foraging on hard organisms

**DOI:** 10.1002/ece3.10332

**Published:** 2023-08-14

**Authors:** Wencke Krings, Charlotte Neumann, Stanislav N. Gorb, Alexander Koehnsen, Heike Wägele

**Affiliations:** ^1^ Department of Electron Microscopy, Institute of Cell and Systems Biology of Animals Universität Hamburg Hamburg Germany; ^2^ Department of Cariology, Endodontology and Periodontology Universität Leipzig Leipzig Germany; ^3^ Department of Mammalogy and Palaeoanthropology Leibniz Institute for the Analysis of Biodiversity Change Hamburg Germany; ^4^ Department of Functional Morphology and Biomechanics, Zoological Institute Christian‐Albrechts‐Universität zu Kiel Kiel Germany; ^5^ Department of Phylogenetics and Evolutionary Biology Leibniz Institute for the Analysis of Biodiversity Change Bonn Germany

**Keywords:** biomineralization, elemental composition, feeding, mechanical properties, Mollusca

## Abstract

The molluscan feeding structure is the radula, a chitinous membrane with teeth, which are highly adapted to the food and the substrate to which the food is attached. In Polyplacophora and Patellogastropoda, the handling of hard ingesta can be facilitated by high content of chemical compounds containing Fe or Si in the tooth cusps. Other taxa, however, possess teeth that are less mineralized, even though animals have to avoid structural failure or high wear during feeding as well. Here, we investigated the gastropod *Gastropteron rubrum*, feeding on hard Foraminifera, diatoms and Porifera. Tooth morphologies and wear were documented by scanning electron microscopy and their mechanical properties were tested by nanoindentation. We determined that gradients of hard‐ and stiffness run along each tooth, decreasing from cusp to basis. We also found that inner lateral teeth were harder and stiffer than the outer ones. These findings allowed us to propose hypotheses about the radula‐ingesta interaction. In search for the origins of the gradients, teeth were visualized using confocal laser scanning microscopy, to determine the degree of tanning, and analyzed with energy‐dispersive X‐ray spectroscopy, to test the elemental composition. We found that the mechanical gradients did not have their origins in the elemental content, as the teeth did not contain high proportions of metals or other minerals. This indicates that their origin might be the degree of tanning. However, in the tooth surfaces that interact with the ingesta high Si and Ca contents were determined, which is likely an adaptation to reduce wear.

## Graphical abstract

Mollusks possess highly adapted radular teeth that allows them to forage on their preferred food. We here investigated the teeth of a colorful sea slug that feeds on hard Foraminifera and Porifera. Teeth contained high content of silica to reduce abrasion. Mechanical property tests revealed that this radula seems to function as a clamp, which can adjust to all kinds of irregular objects.

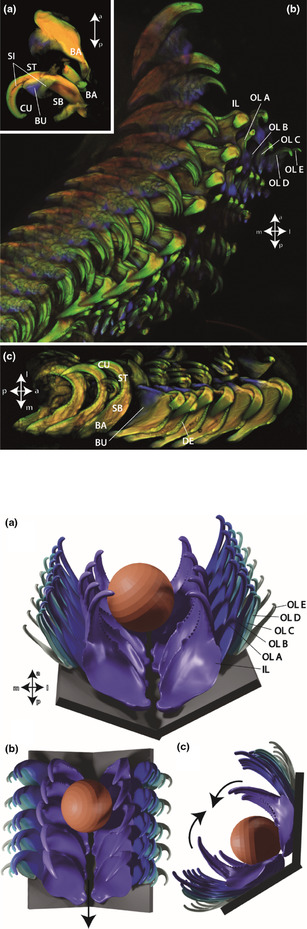

## 1 INTRODUCTION

The radula is one important molluscan apomorphy and consists of a chitinous membrane with rows of embedded teeth. Radulae can show adaptations to the preferred ingesta (i.e., food, particles on the food, substrate that the food is attached to) in morphology and the arrangement of teeth on the membrane (e.g., Hawkins et al., 1989; Krings, Karabacak, & Gorb, 2021; Krings, Kovalev, & Gorb, 2021b, 2021c; Padilla, 2003; Solem, 1972, 1974; Steneck & Watling, 1982; Ukmar‐Godec et al., 2015).

Even though the radula is constantly renewed by secretion in the posterior radular region (the “radular sac”) by over‐ and underlain epithelia (Mackenstedt & Märkel, 1987; Runham, 1963; Runham & Isarankura, 1966; Vortsepneva et al., 2022), the radular material properties are adapted to reduce wear and/or structural failure, induced by the specific ingesta source, as well. Structural failure can be reduced by the presence of mechanical property gradients (i.e., of the Young's modulus) along each tooth. In polyplacophorans, limpets, or some gastropod taxa (e.g., some members of the Paludomidae and Nudibranchia), the radula needs to transfer high forces to solid surfaces (e.g., rocks) by scratching action (e.g., Herrera et al., 2015; Krings et al., 2022a, 2022d; Lu & Barber, 2012; van der Wal et al., 1999; Weaver et al., 2010) or to hard structures of the prey (e.g., sponge spiculae) by piercing action (Krings et al., 2023). Here, each tooth shows pronounced gradients with the cusp as the hardest and stiffest region, followed by the stylus and finally the basis, as the softest and most flexible region (Gorb & Krings, 2021; Herrera et al., 2015; Krings et al., 2019, 2022c, 2022d, 2023; Lu & Barber, 2012; Pohl et al., 2020; van der Wal et al., 1999; Weaver et al., 2010). This allows teeth to bend and to either gain support from the next row of teeth, which redistributes the stress, or to deform and adjust to the prey item to avoid structural damage. These mechanical property gradients have their origin in the degree of tanning, the content of inorganics, the regional water content, or the chitin fiber arrangement (e.g., Brooker & Shaw, 2012; Faivre & Ukmar‐Godec, 2015; Joester & Brooker, 2016; Krings et al., 2022d; Krings, Kovalev, & Gorb, 2021b).

With regard to abrasion resistance, some taxa, like Polyplacophora and Patellogastropoda, incorporate high proportions of iron and silicon into their very thick tooth leading edge (i.e., the surface of the tooth that interacts directly with the ingesta) resulting in hard tooth cusps as adaptation to feeding from algae growing on stones (e.g., Barber et al., 2015; Han et al., 2011; Krings et al., 2022c; Lu & Barber, 2012; Saunders et al., 2011; Shaw et al., 2009a, 2009b, 2010; van der Wal et al., 1999; Wang et al., 2014; Wealthall et al., 2005; Weaver et al., 2010). High inorganic contents such as Ca or Si were also found on the leading edges (“leading surfaces”) of other gastropod taxa (e.g., some Paludomidae foraging on algae from rocks and Nudibranchia foraging on Porifera) as well. However, the coating was very thin in comparison to the leading edge of Polyplacophora, suggesting that these teeth resemble highly functional lightweight structures (Krings et al., 2022a, 2023). Also, teeth that come in contact with abrasive particles, like sand, were found to contain a thin layer with high content of Ca on all tooth surfaces, presumably to prevent high wear (Krings & Gorb, 2023a). However, for most gastropod taxa there are huge gaps in knowledge.

In this context, we here aim at unraveling the functional principles that reflect adaptation to this hard and abrasive ingesta source in the radular teeth of the gastropod *Gastropteron rubrum* (Rafinesque, 1814; Heterobranchia, Euopisthobranchia), which forages on Foraminifera (DeLaHoz et al., 2018). This species belongs to the Cephalaspidea, which are known for their predation on Foraminifera (Berry & Thomson, 1990; Cedhagen, 1996; Chester, 1993; Eilertsen & Malaquias, 2013; Hurst, 1965; Malaquias et al., 2004; Rudman, 1972a, 1972b; Shonman & Nybakken, 1978; Thompson, 1976, 1988). This is the first tooth analysis of a gastropod that mainly preys on this kind of food. First, the stomach content of the sea slug was documented to gain insight into the food composition in detail. Then, the teeth were carefully documented unraveling the wear at the tooth cusps. The mechanical properties of the teeth were tested to get insights into the mechanical adaptations of the radular apparatus. In the search for the origins of the mechanical properties in the teeth, we also investigated the material composition.

## 2 MATERIALS AND METHODS

### 2.1 Specimens and preparation

Individuals of *Gastropteron rubrum* were collected by Yvonne Grzymbowski at Els Capets, Costa Brava, Spain, in November 2004, and fixed in 96% EtOH. Eight adult specimens were dissected for this study.

### 2.2 Scanning electron microscopy (SEM) and 3D visualization

For documentation of morphology using SEM, three radulae were carefully extracted and cleaned by a short ultrasonic bath in 70% EtOH. Subsequently they were mounted on SEM specimen holders by double‐sided adhesive carbon tape and sputter‐coated with platinum (5 nm layer). For visualization, we used a SEM Zeiss LEO 1525 (One Zeiss Drive). Only mature teeth from the working zone, which can be identified by lack of covering epithelia, were studied. To document the wear on the teeth, radulae were rewetted by 70% EtOH afterwards, cleaned by a short ultrasonic bath, rearranged on SEM sample holders and visualized again in the SEM. Nomenclature of teeth was adapted from Ong et al. (2017).

For the 3D visualization, mature radular teeth of the working zone of two radulae were extracted manually with forceps. Each tooth was mounted on SEM specimen holders by double‐sided adhesive carbon tape, sputter‐coated with platinum (5 nm layer), and visualized under the SEM from all sides. Using the 3D software Blender v2.83 (Blender Foundation), the teeth were then modeled by hand constantly comparing the 3D visualization with the SEM images taken from different sides (see also protocol in Krings, Marcé‐Nogué, et al., 2020; Krings, Marcé‐Nogué, & Gorb, 2021). In the same manner, the position and embedment of the teeth within the membrane were reconstructed.

### 2.3 Ingesta analyses

For ingesta analyses, the intestines of four specimens were opened and the particles carefully extracted by tweezers. We differentiated between ingesta in the proximal and the distal part of the intestine.

### 2.4 Confocal laser scanning microscope (CLSM)

To document the autofluorescence of the tooth material, two cleaned radulae and some individual mature teeth were arranged on object glass slides, following the procedure of Michels and Gorb (2012). Each radula was surrounded by a stack of reinforcement rings. The rings were filled with glycerin (greater than or equal to 99.5%, free of water, Carl Roth GmbH & Co. KG) and subsequently covered by a glass cover slip. Following the protocol of Krings et al. (2022d, 2023), samples were visualized employing a Zeiss LSM 700 confocal laser scanning microscope (Carl Zeiss Microscopy GmbH). Four stable solid‐state lasers with wavelengths of 405, 488, 555, and 639 nm were used. Bandpass or longpass emission filters (420–480 nm, greater than or equal to 490 nm, greater than or equal to 560 nm, or greater than or equal to 640 nm) were applied. After scanning, images of autofluorescence were superimposed (with maximum intensity projection) using the software Zeiss Efficient Navigation (Zen; Carl Zeiss MicroImaging GmbH). Finally, the color blue was assigned to the autofluorescence signal received from the laser with wavelength 405 nm, green to 488 nm, red (50% saturation) to 555 nm and red (50% saturation) to 639 nm.

### 2.5 Energy dispersive X‐ray spectroscopy (EDX)

For analysis of the elemental composition, three cleaned radulae (ultrasonic bath for 20 s) were attached to glass object slides by double‐sided adhesive tape, following our previous protocol (Krings et al., 2022a, 2022c, 2023). Then, each radula was surrounded by a small metallic ring. Afterwards the ring was filled with epoxy resin (Reckli Epoxy WST, RECKLI GmbH) to cover the radula completely. After polymerization, lasting for 3 days at room temperature, glass object slides, and adhesive tape were removed. Samples were polished with sandpapers of different roughness, until the cross‐sections of teeth were on display, and smoothened with aluminum oxide polishing powder suspension of 0.3 μm grainsize (PRESI GmbH) on a polishing machine (Minitech 233/333, PRESI GmbH) to receive a plain smooth surface. The embedding and smoothening prevent artifacts such as electron scattering during EDX analysis. Embedded samples were subsequently cleaned in an ultrasonic bath for 5 min, then mounted on SEM sample holders and sputter‐coated with platinum (5 nm layer). Elemental composition was determined with the SEM Zeiss LEO 1525 equipped with an octane silicon drift detector (SDD; micro analyses system TEAM, EDAX Inc.). For each sample, the same settings were used (i.e., an acceleration voltage of 20 kV, working distance, lens opening, etc.). Before analysis, the detector was calibrated with copper.

Small areas (no mapping) were analyzed to receive the data. Following elements were detected and their proportions measured: H (hydrogen), C (carbon), N (nitrogen), O (oxygen), Pt (platinum), Al (aluminum), Ca (calcium), Cl (chlorine), Cu (copper), Fe (iron), K (potassium), Mg (magnesium), Na (sodium), P (phosphorus), S (sulfur), Si (silicon), and Zn (zinc). Some elements were not discussed as they are either the elemental basis of chitin and proteins (H, C, N, O), the coating (Pt), or the polishing powder (Al, O). For test purposes, we also performed 10 EDX tests on the epoxy to identify putative pollution due to the mechanical application, embedding or polishing. We could not detect Si (which is part of the sandpaper), or any other elements, that we further discuss as Ae (Ca, Cl, Cu, Fe, K, Mg, P + Pt, S, Si, Zn), in the resin. Their presence is therefore considered part of the teeth.

The single peak of P overlaps with one of Pt. Due of this, the software could not discriminate between these two elements and P content could not be reliably determined. Therefore, P and Pt are discussed together (P + Pt). We, however, measured 20 areas of pure epoxy to receive values on their Pt content (mean ± SD; 0.15 ± 0.02 atomic %) to further estimate the proportions of P in the teeth.

**FIGURE 1:**
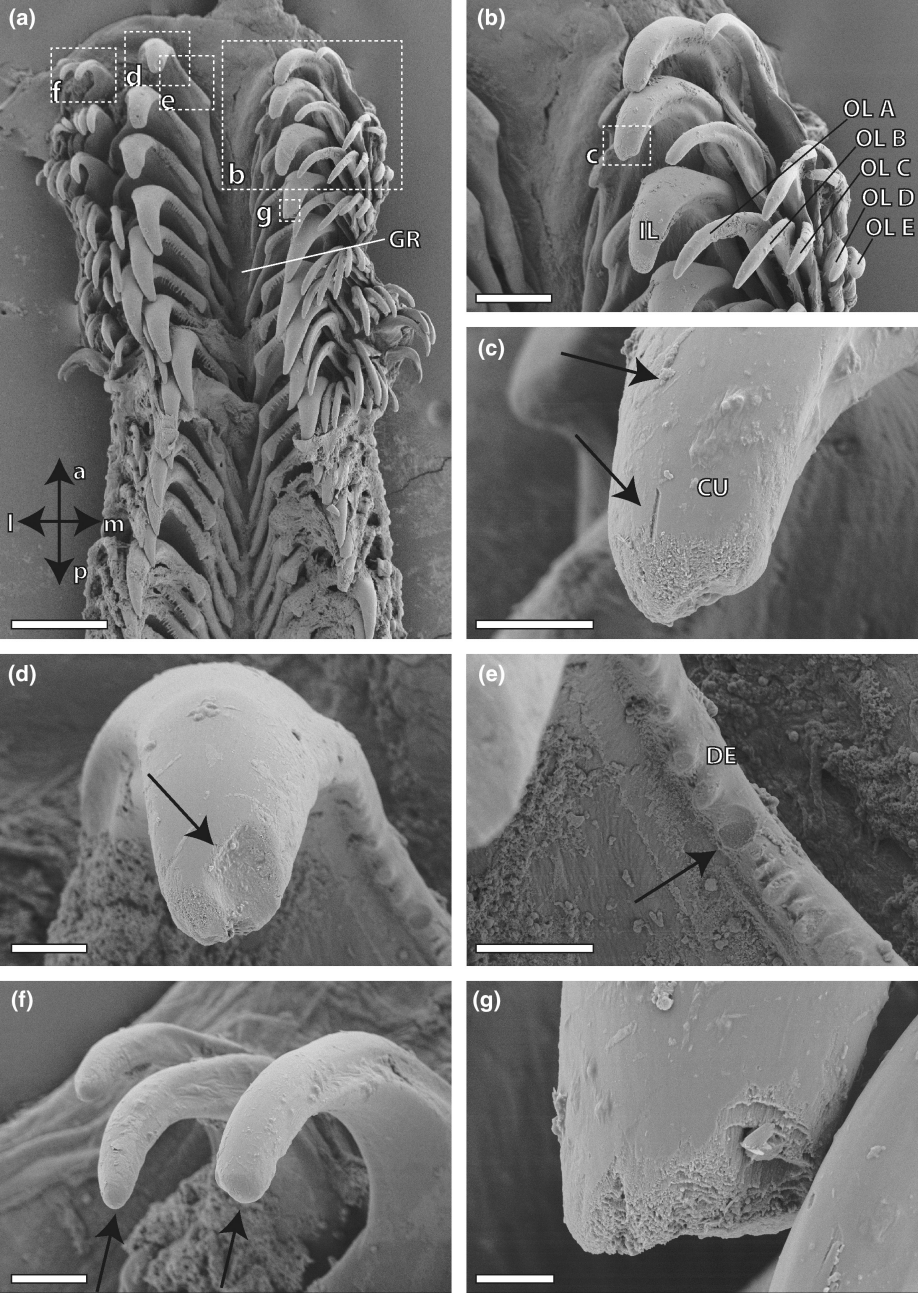
(a) Radula, overview with anatomical directions. (b–g) Magnifications, displaying cracks and wear (highlighted by arrows), probably resulting from interaction with hard ingesta particles (stones, spicules, sand, Foraminifera tests). a, anterior (towards the degenerative zone); CU, cusp; DE, denticle; GR, groove; IL, inner lateral; l, lateral; m, medial; OL A, outer lateral A; OL B, outer lateral B; OL C, outer lateral C; OL D, outer lateral D; OL E, outer lateral E; p, posterior (towards the building zone). Scale bars: (a) 200 μm; (b) 80 μm; (c–f) 20 μm; (g) 8 μm.

**FIGURE 2:**
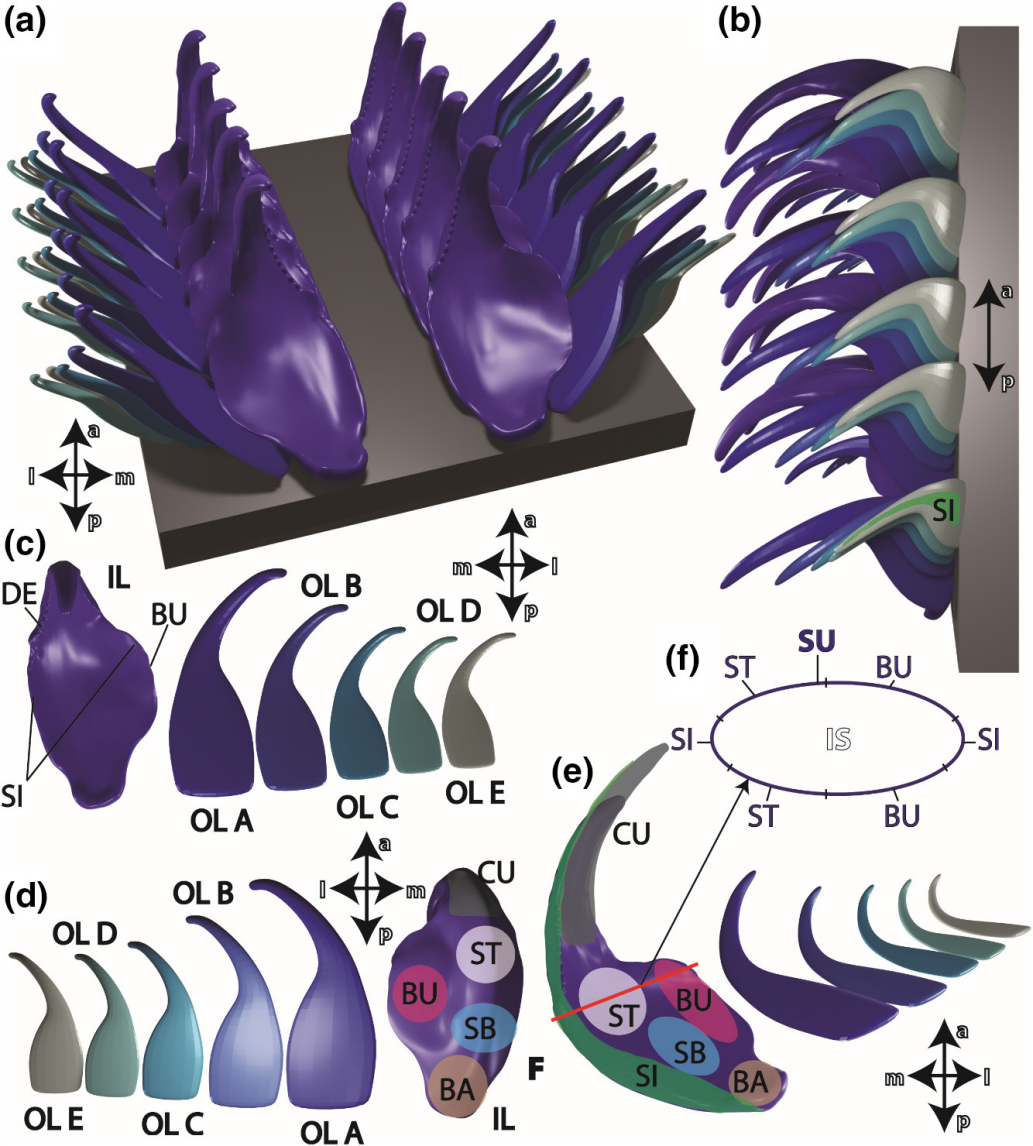
3D model of the radula to introduce the nomenclature of tooth regions and the sites that were tested. (a) Radula from above. (b) Radula from the side. (c) The individual teeth from above. (d) From below. (e) Teeth from medial view, the red line indicates a cross section, from where point‐measurements were taken via EDX. (f) Schematic outline of a characteristic polished section area of a tooth. When this area was on display, the inner structure and the surfaces could be tested with point‐measurements via EDX. The surface was separated into different regions (surface of e.g., the bulge, the stylus, the sides). a, anterior (towards the degenerative zone); BA, basis; BU, bulge; CU, cusp; DE, denticle; IL, inner lateral; IS, inner structure; l, lateral; m, medial; OL A, outer lateral A; OL B, outer lateral B; OL C, outer lateral C; OL D, outer lateral D; OL E, outer lateral E; p, posterior (towards the building zone); SB, basis of stylus; SI, side; ST, terminal stylus; SU, surface.

We tested the inner tooth structure by EDX and the thin outer layer (“surface”) of the teeth (500–1000 nm thickness), which covers the inner tooth structure. We did not detect high content of elements in the inner structure and no differences in the distribution there. We thus decided to summarize the point measurements of the inner structure. With regard to the surfaces, we could determine variations in the distribution of elements and thus differentiated between the tooth basis, the bulges, the basal region of the stylus (stylus, basis), the terminal region of the stylus (stylus, terminal), the cusps and the sides (see Figures 1 and 2 for nomenclature). 474 point measurements on 180 mature teeth were conducted: 204 on 70 inner laterals (of which 70 on the inner tooth structure and 134 on the surface); 56 (22 on the inner tooth structure and 34 on the surface) on 22 outer laterals A, B, C; 54 (22 on the inner tooth structure and 32 on the surface) on 22 outer laterals D; 48 (22 on the inner tooth structure and 26 on the surface) on 22 outer laterals E.

### 2.6 Nanoindentation

**FIGURE A1:**
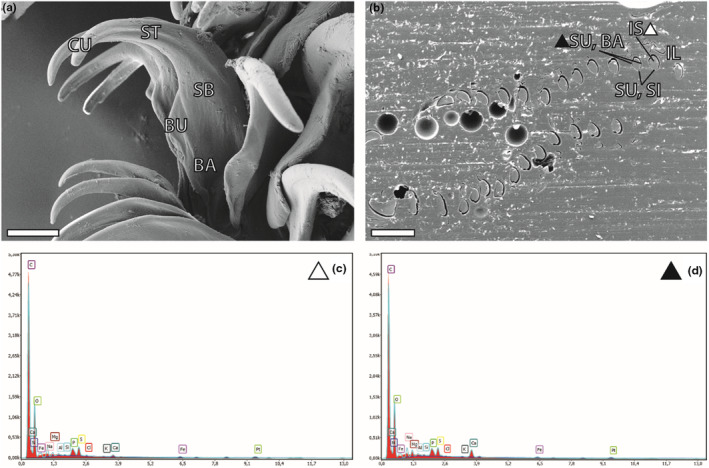
(a) SEM image of teeth. (b) Embedded radula sample for EDX analysis and nanoindentation experiments. Representative EDX spectra of the inner tooth structure (c) and the surface at the tooth basis (d). BA, basis; BU, bulge; CU, cusp; IL, inner lateral tooth; IS, inner tooth structure; SB, basis of stylus; SI, side; SU, tooth surface; ST, terminal stylus. Scale bars: (a) 40 μm; (b) 0.5mm.

To test the mechanical properties, nanoindentation experiments were performed on three additional radulae (for detailed protocol see Gorb & Krings, 2021; Krings et al., 2022c, 2022d, 2023). Radulae were arranged on glass object slides and surrounded by a small metallic ring. Afterwards, each ring was filled with epoxy resin, which covered the radula completely. After polymerization, samples were polished with sandpapers until tooth sections were on display (see Figure A1), and smoothened with aluminum oxide polishing powder suspension on a polishing machine. Samples were cleaned in an ultrasonic bath for 5 min. A nanoindenter SA2 (MTS Nano Instruments) equipped with a Berkovich indenter tip and a dynamic contact module head was employed. Hardness (*H*) and Young's modulus (*E*) were determined from force‐distance curves by applying the continuous stiffness mode. All tests were performed under normal room conditions (relative humidity 28%–30%, temperature 22–24°C) and each indent and corresponding curve were both manually controlled. After this, samples were smoothened and polished until the next target localities were on display.

Overall, the inner structure of each tooth was tested at five localities to receive data on mechanical property gradients within each tooth. *E* and *H* were determined at penetration depths of 500–1000 nm. For each site indented, we received ~60 values obtained at different indentation depths, which were averaged to receive one *H* and one *E* mean value per indent. 413 localities were overall tested: 118 on the inner lateral, 59 on the outer lateral A, outer lateral B, outer lateral C, outer lateral D, and outer lateral *E*, respectively.

### 2.7 Statistical analyses

All statistical analyses were performed with JMP Pro, Version 14 (SAS Institute Inc., 1989–2007). Mean values and standard deviations were calculated and Shapiro–Wilk‐*W*‐tests for testing of normality were conducted. As the data was non‐normally distributed, a Kruskal–Wallis/Wilcoxon test, followed by pairwise comparison with Wilcoxon method, was carried out.

## 3 RESULTS

### 3.1 Morphology and wear of teeth

Individuals of *Gastropteron rubrum* possessed one prominent inner lateral tooth, followed by five outer laterals to each side of the radula, per row (see Figures 1a and 2a–f). The size of these outer laterals decreased towards the margin of the radula. A central tooth was missing and the prominent inner laterals were separated by a groove. Each inner lateral contained about 10 small denticles on the medial side and a bulge on the lateral side. Each outer lateral possessed one large cusp (tip).

Using SEM, we investigated the wear of each tooth type. On the inner laterals, spalling (Figure 1d,e,g) and scratches (Figure 1c) on the cusps and the denticles were found (see Figure 1a–e); in some cases, structural loss was rather high on these regions (Figure 1g). In contrast, we did not find scratches or spalling on the outer laterals (Figure 1f); the degree of wear decreased towards the outer laterals.

### 3.2 Ingesta analyses

**FIGURE 3:**
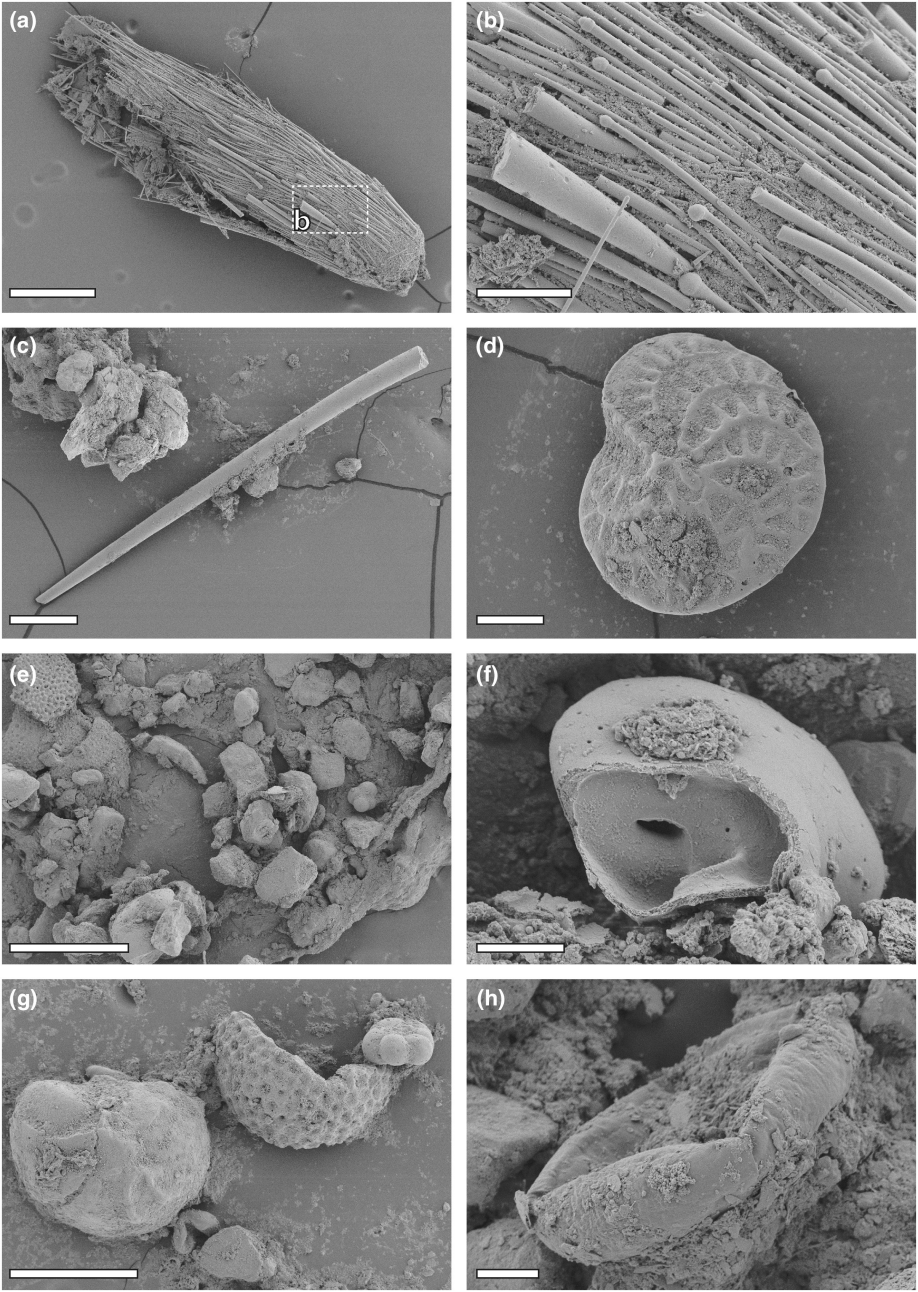
Intestine content. (a–c) Spiculae, including the tylostyle type from Heteroscleromorpha (Porifera). (d) Foraminifera tests, extracted from the proximal region of the intestine. (e) Intestine content with tests of Foraminifera and stones. (f) Cracked Foraminifera tests. (g) Foraminifera tests from the distal region of the intestine. (h) Diatom. Scale bars: (a) 400 μm; (b, d) 80 μm; (c, g) 200 μm; (e) 400 μm; (f) 20 μm; (h) 40 μm.

Analyses of the intestine revealed, that *G. rubrum* took in sand particles, Foraminifera and diatoms (Figure 3d–g). However, spicules of the tylostyle type were also found, which shows that Heteroscleromorpha sponges, like Axinellida, Biemnida, Merliida, Polymastiida, Clionaida, Tethyida, and Suberitida were eaten (see Morrow & Cárdenas, 2015; Figure 3a–c). The prey structures extracted from the distal region of the intestine (see Figure 3g) were more brittle and fractured than those from the proximal region of the intestine (compare with Figure 3d).

### 3.3 Autofluorescence signals

**FIGURE 4:**
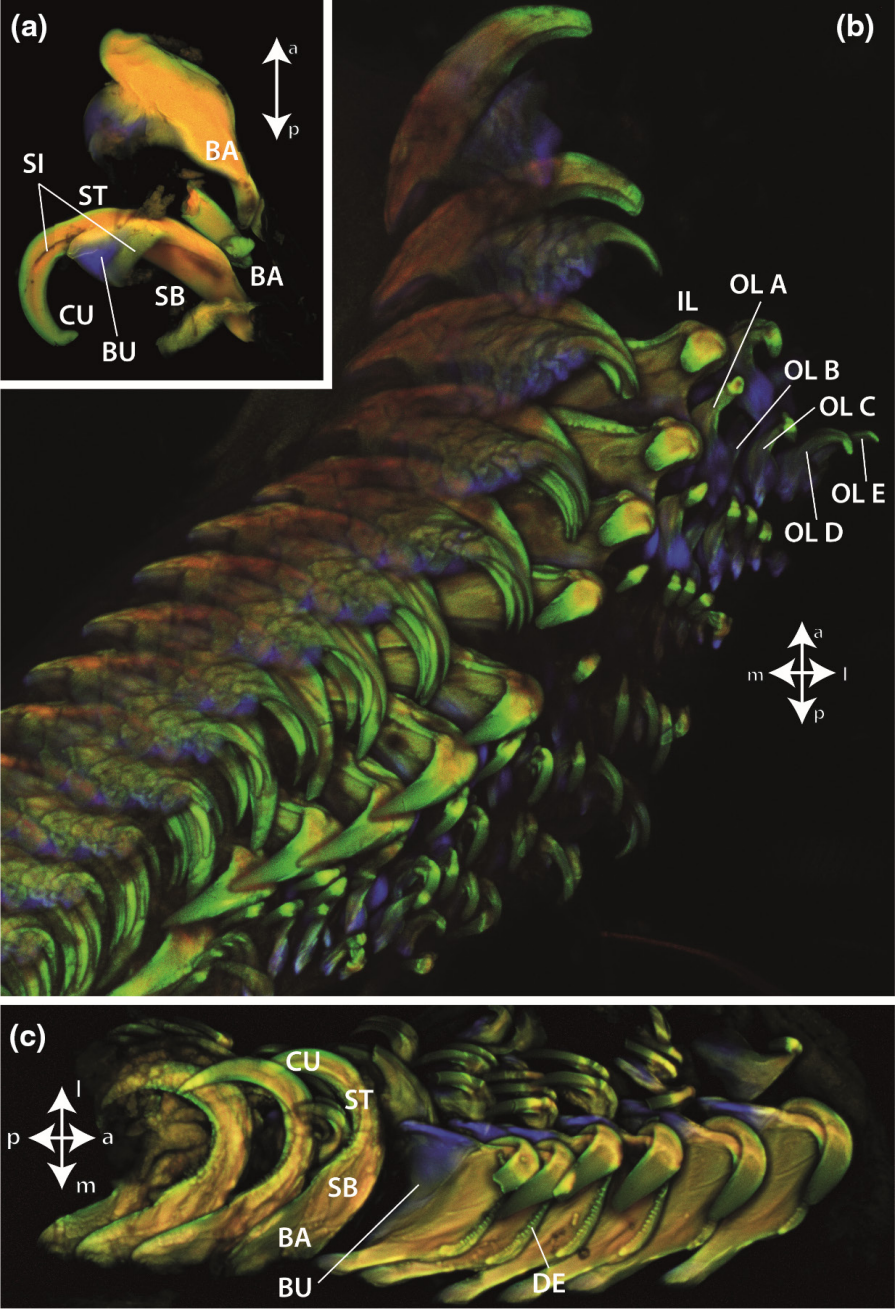
CLSM images of *Gastropteron rubrum* radula. (a) Mature inner lateral teeth. (b) Whole radula. (c) One side of the radular working zone. a, anterior (towards the degenerative zone); BA, basis; BU, bulge; CU, cusp; DE, denticle; IL, inner lateral; l, lateral; m, medial; OL A, outer lateral A; OL B, outer lateral B; OL C, outer lateral C; OL D, outer lateral D; OL E, outer lateral E; p, posterior (towards the building zone); SB, basis of stylus; SI, side; ST, terminal stylus.

All cusps and denticles from the inner lateral teeth emitted a strong green autofluorescence signal on their anterior and posterior surfaces (i.e., they emitted a strong signal after excitation with the 488 nm laser). The cusps' lateral surfaces, the tooth styli and bases appeared rather red‐ to yellow‐brown (i.e., emitted a strong signal after excitation with the lasers of 555 and 639 nm wavelength) (see Figure 4). The bulges and bases of the teeth exhibited a strong blue signal (i.e., emitted a strong signal after excitation with the 405 nm laser).

### 3.4 Elemental analysis by EDX

**FIGURE 5:**
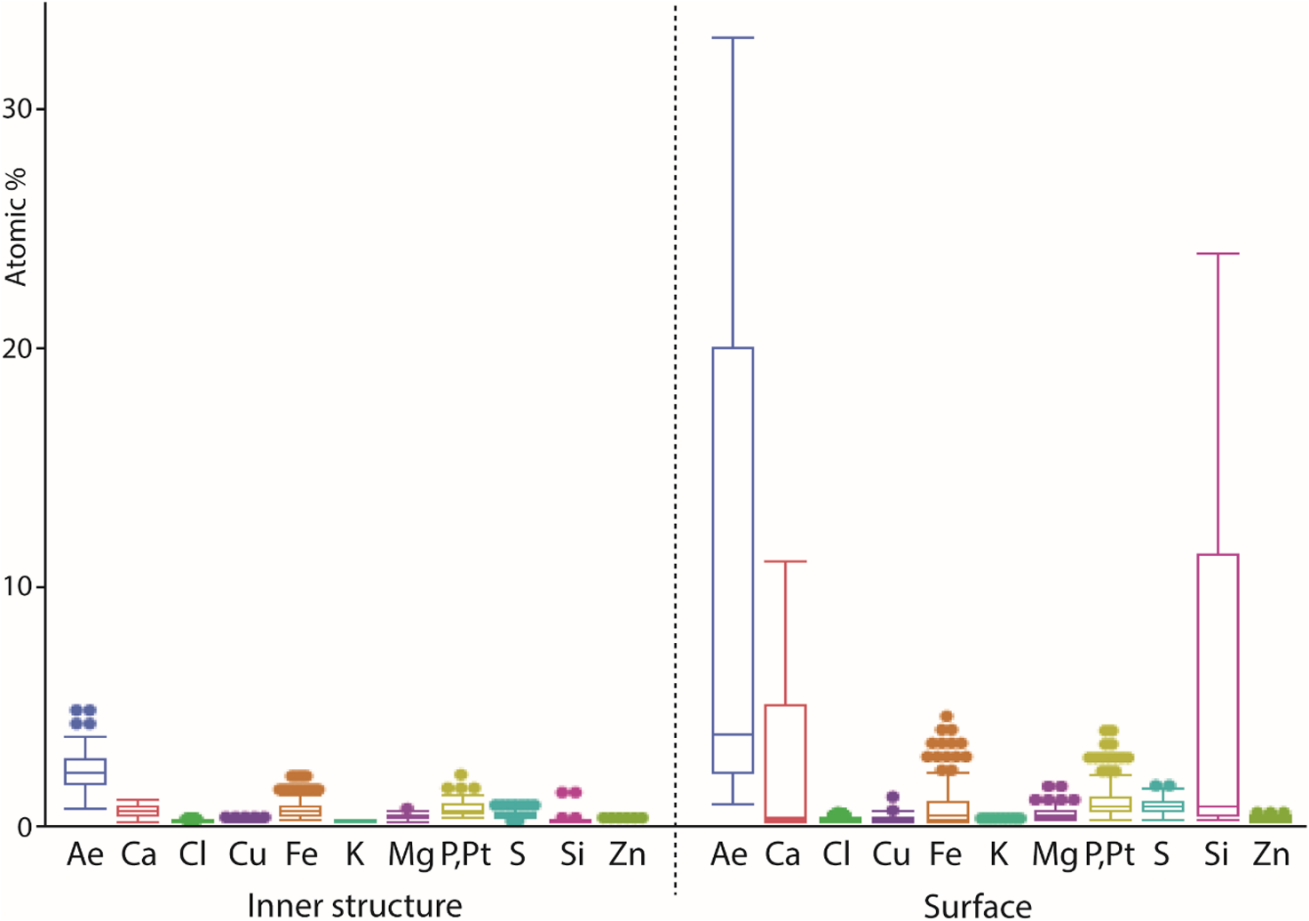
Results from the EDX analysis. Content of Ca, Cl, Cu, Fe, K, Mg, P + Pt, S, Si, Zn, and Ae (sum of Ca, Cl, Cu, Fe, K, Mg, P + Pt, S, Si, and Zn) for the inner tooth structure and the tooth surface (all tooth types and regions are pooled together).

EDX can determine the elements present, but not the bonding conditions. We detected Ca, Cl, Cu, Fe, K, Mg, P + Pt, S, Si, and Zn in the teeth (see Figure 5). The content of each individual element, except Fe, showed highly significant differences between the inner structure and the surface (results from Wilcoxon‐test: *p* < .0001*, for *p*‐values see Table A1).

**FIGURE 6:**
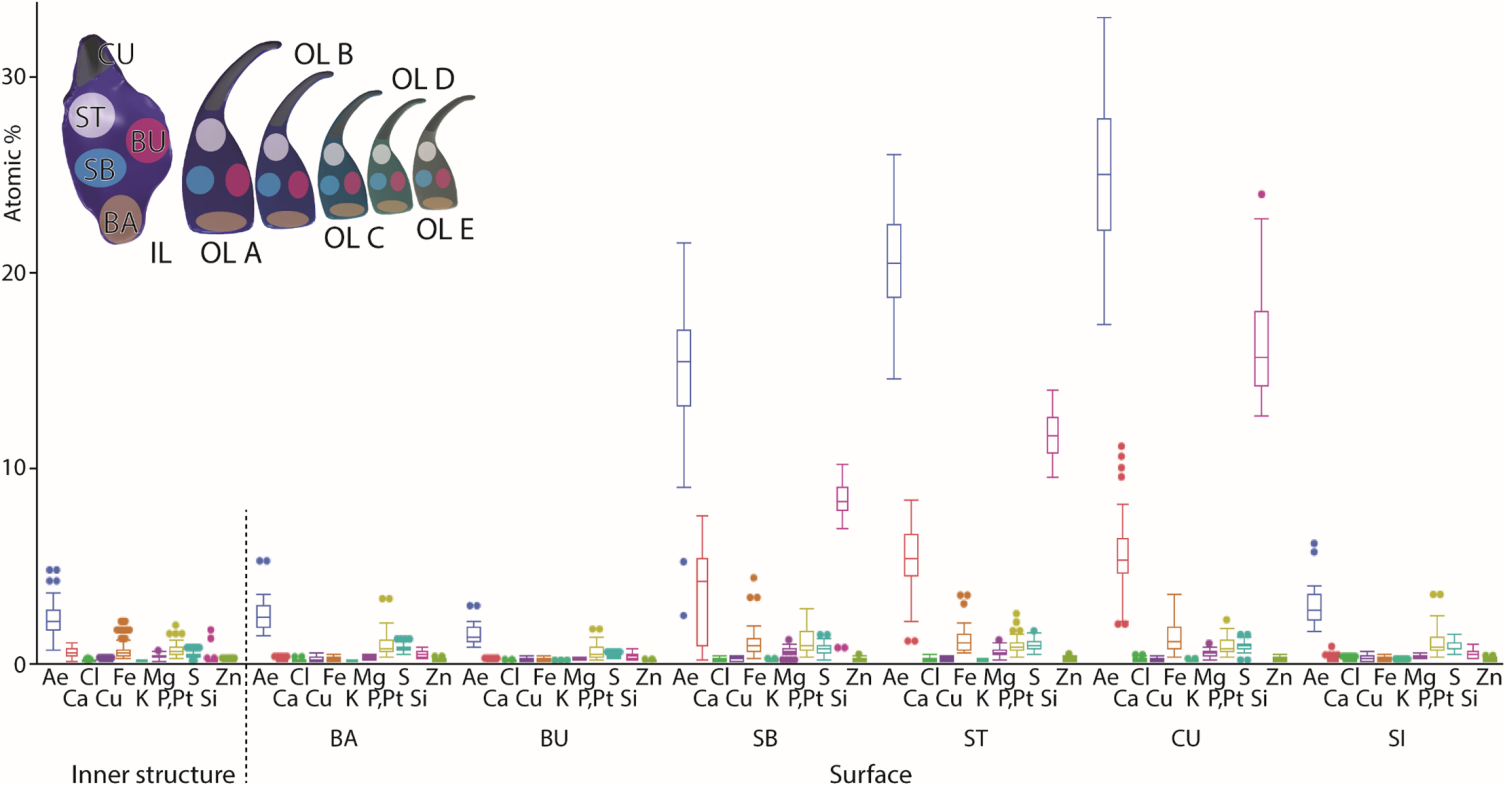
Results from the EDX analysis. Contents of Ca, Cl, Cu, Fe, K, Mg, P + Pt, S, Si, Zn, and Ae (sum of Ca, Cl, Cu, Fe, K, Mg, P + Pt, S, Si, and Zn) for the inner tooth structure and the tooth surface regions (tooth types are pooled together). BA, basis; BU, bulge; CU, cusp; IL, inner lateral; OL A, outer lateral A; OL B, outer lateral B; OL C, outer lateral C; OL D, outer lateral D; OL E, outer lateral E; SB, basis of stylus; SI, side; ST, terminal stylus.

In the inner tooth structure, the following proportions were found (sorted from high to low mean content): P + Pt (mean ± standard deviation: 0.56 ± 0.26 atomic %), Fe (0.53 ± 0.35), Ca (0.47 ± 0.23), S (0.34 ± 0.12), Mg (0.26 ± 0.09), Zn (0.06 ± 0.03), Cu (0.05 ± 0.04), Si (0.04 ± 0.13), Cl (0.03 ± 0.03), and K (0.03 ± 0.02). All lateral teeth showed a rather small proportion of these elements in the inner tooth structure, compared to the surface (see below; see Table A2 for elemental content in the inner structure of the different teeth; see Figure 6). Between the tooth types, we could not detect significant differences by pairwise comparison for the individual elemental contents (see Table A3 for *p*‐values).

In the surface, the following proportions were detected (sorted from high to low mean content): Si (mean ± standard deviation: 5.79 ± 6.40 atomic %), Ca (2.28 ± 2.28), P + Pt (0.85 ± 0.60), S (0.67 ± 0.30), Fe (0.60 ± 0.72), Mg (0.34 ± 0.20), Cu (0.14 ± 0.11), Zn (0.12 ± 0.08), Cl (0.08 ± 0.08), and K (0.05 ± 0.03). In each tooth, especially Si and Ca were present in larger proportions in comparison to the other elements (see Table A2 for elemental content of the surfaces).

**FIGURE A2:**
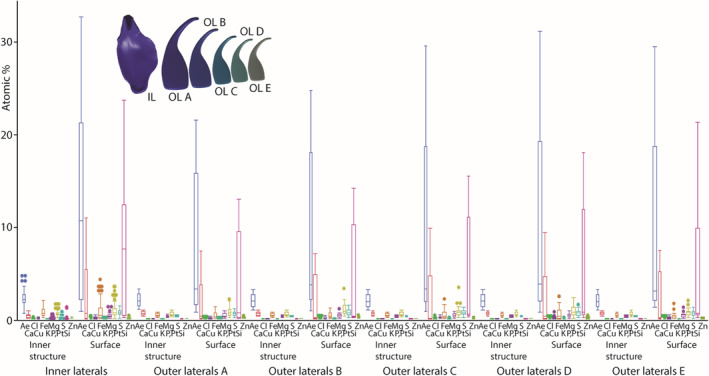
Results from EDX analysis. Content of Ca, Cl, Cu, Fe, K, Mg, P + Pt, S, Si, Zn, and Ae (sum of Ca, Cl, Cu, Fe, K, Mg, P + Pt, S, Si, and Zn) for the inner tooth structure and the tooth surface for each tooth type. Regions of tooth surfaces are pooled together.

When the results from the tooth surfaces were sorted according to the tooth region (see Figure 2 for nomenclature and six for results), we found that the cusps contained highest contents of all elements (Ae), followed by the styli (terminal), the styli (bases), the sides, the tooth bases, and finally the bulges with the lowest content of Ae (see Table A4). This gradient in Ae was primarily caused by the content and distribution of Si (at cusps, mean ± standard deviation: 16.24 ± 2.76 atomic %; at bulges: 0.27 ± 0.14) and Ca (at cusps: 5.56 ± 2.01; at bulges: 0.05 ± 0.05). But Fe, which was present in lower proportions, probably also contributes to the gradient (at cusps: 1.26 ± 0.75; at bulges: 0.09 ± 0.07; see Table A4 for elemental content). The decreases of Si, Ca and Fe from tips to bases were found in each tooth type (see Table A5 for elemental contents of the different teeth and their regions). When comparing the various regions of each tooth, differences were highly significant (see Table A6 for *p*‐values). For the distribution of Cl, Cu, K, Mg, P + Pt, S, and Zn, no clear gradient could be detected, even though most regions differed highly significantly as determined by pairwise comparison (see Table A6 for *p*‐values). These elements were present in rather small proportions in each tooth (mean is <1 atomic %; see Tables A2 and A4). When comparing the different tooth types, we could not determine clear differences between them (see Figure A2). In most cases, we detected highly significant differences between the individual regions of the surface for Si, Ca, and Fe, when comparing the different teeth by pairwise comparison (see Table A3 for *p*‐values).

### 3.5 Mechanical properties

The hardness (*H*) describes the resistance to local plastic deformation induced by abrasion or indentation. The Young's modulus (*E*) is the measure of the stiffness of a solid material and describes the relationship between tensile stress and axial strain.

**FIGURE 7:**
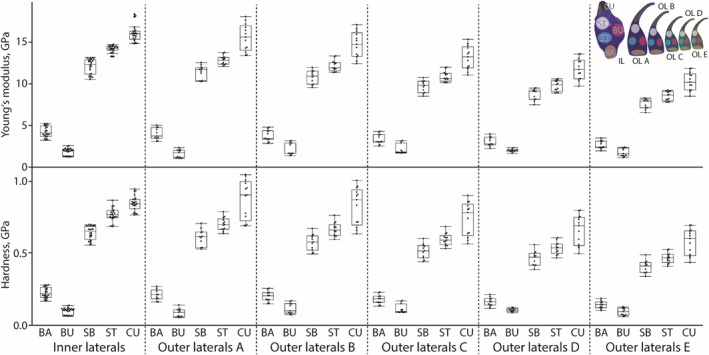
Results from the nanoindentation experiments. Young's modulus and hardness (both given in GPa) for the different teeth and their regions. BA, basis; BU, bulge; CU, cusp; SB, basis of stylus; ST, terminal stylus. BA, basis; BU, bulge; CU, cusp; IL, inner lateral; OL A, outer lateral A; OL B, outer lateral B; OL C, outer lateral C; OL D, outer lateral D; OL E, outer lateral E; SB, basis of stylus; SI, side; ST, terminal stylus.

In every tooth, the cusp (*E* mean values range between 10.27 and 15.95 GPa; *H* mean values range between 0.58 and 0.85 GPa) was always the stiffest and hardest region, followed by the terminal stylus (*E* = 8.51–14.18 GPa; *H* = 0.47–0.77 GPa), the basis of the stylus (*E* = 7.58–12.09 GPa; *H* = 0.41–0.64 GPa), the basis (*E* = 2.64–4.23 GPa; *H* = 0.14–0.23 GPa), and finally the bulge as the softest and most flexible region (*E* = 1.75–1.84 GPa; *H* = 0.09–0.10 GPa). The parts of the inner laterals were harder and stiffer, followed by the outer laterals A, B, C, D, and finally *E* with the softest and most flexible parts (see Figure 7 and Table A7 for all values).

Pairwise comparison by Wilcoxon method revealed, that the regions within each tooth showed highly significant differences (*p* < 0.0001*; see Table A8 for all *p*‐values). Most regions were also highly significantly different, when they were compared between the teeth (mostly *p* < 0.0001*; see Table A8 for all *p*‐values).

Despite the differences of *E* and *H* values along the various regions of each tooth and between teeth, the two values always exhibited a very high positive correlation (*r* = 0.99, *p* < 0.0001*).

### 3.6 Relationship between autofluorescence, elemental composition, and mechanical properties

The regions of the teeth appearing green under CLSM (i.e., emitted a strong signal after excitation with the 488 nm laser) contained high proportions of Si and Ca. The sides of the stylus and the bases appeared red‐ to yellow‐brown (i.e., emitted a strong signal after excitation with the lasers of 555 and 639 nm wavelength); here no high contents of Ca and Si were detected. The bulges appeared blue (i.e., emitted a strong signal after excitation with the lasers of 405 nm wavelength); these regions contained no high content of the targeted elements and were the softest and most flexible tooth parts. We could not determine relationships between the elemental content of the inner tooth structure and the mechanical property values.

## 4 DISCUSSION

### 4.1 Mechanical behavior and foraging

The mechanical properties of materials directly contribute to the mechanical behavior of structures. The Young's modulus (*E*) relates to the ability of a structure to transmit force (Bendsøe, 1989, 1995; Bendsøe & Kikuchi, 1988; Dumont et al., 2009), its resistance to failure as well as the structures' mechanical behavior while puncturing (e.g., Freeman & Lemen, 2007; for review on puncture mechanics see Anderson, 2018). The hardness (*H*) is the measure of the resistance to local plastic deformation induced by indentation or abrasion.

Some gastropod species (i.e., paludomid taxa) feeding on soft ingesta (i.e., algae growing on soft substrates like sand or mud) possess soft and more flexibles teeth (*E* ≤ 8 GPa, *H* ≤ 1 GPa) without clear and pronounced gradients in mechanical properties from the tooth basis across the stylus to the cusp (Gorb & Krings, 2021). These teeth are probably not capable of transferring high forces without structural failure, but possess an increased ability to deform, bend, and twist, which reduces the risk of breaking (Krings, Kovalev, & Gorb, 2021b, 2021c). Since all teeth within one row have similar mechanical properties, they probably also have a similar function (“monofunctional radula”; see Gorb & Krings, 2021; Krings, 2020).

Species foraging on the solid ingesta (members of Paludomidae foraging on algae covering rocks, Patellogastropoda, Fissurellidae, Polyplacophora) or have some interactions with hard ingesta (the nudibranch gastropods *Felimare picta* and *Doris pseudoargus* feeding on Porifera with hard spiculae) possess harder and stiffer teeth reducing wear and structural failure. Each tooth shows pronounced gradients in both *H* and *E*: the cusp (especially the leading edge) is the hardest and stiffest part, followed by the stylus, and finally the basis as the softest and most flexible part (Barber et al., 2015; Gorb & Krings, 2021; Grunenfelder et al., 2014; Krings et al., 2019, 2022c, 2022d, 2023; Lu & Barber, 2012; Ukmar‐Godec et al., 2017; Weaver et al., 2010). In the above mentioned taxa, the tooth cusps puncture the ingesta or scratch across solid surfaces, with the possible formation of local stress at the cusps, but without high degrees of wear or structural failure. The softer and more flexible stylus, together with the basis, provides flexibility and act as shock absorber against mechanical impacts (Gorb & Krings, 2021; Herrera et al., 2015; Krings et al., 2019, 2022c, 2022d, 2023; Pohl et al., 2020).

The dominant teeth of the Polyplacophora and *Patella vulgata* (Patellogastropoda) are characterized by a very high inorganic content and high *E* and *H* values (see Table 1; Barber et al., 2015; Grunenfelder et al., 2014; Krings et al., 2022c; Lu & Barber, 2012; Weaver et al., 2010). Less mineralized teeth are softer and more flexible: in the vetigastropod *Megathura crenulata* (Fissurellidae), *E* values of 16 GPa were determined (see Table 1; Ukmar‐Godec et al., 2017). In the two investigated nudibranch species, where the inner structure of teeth also contained low inorganic content, *E*_max_ values of 15 GPa and *H*_max_ values of 0.9 GPa were found (see Table 1; Krings et al., 2023). Their thin leading surfaces were however significantly harder (*H*_max_ = 2.3 GPa) and stiffer (*E*_max_ = 45 GPa) than the inner structure, due to high proportions of Si or Ca. The unmineralized teeth of paludomid gastropods foraging from solid surfaces were even softer and more flexible in comparison to the inner structure of the nudibranch taxa (*H* = ~0.4 GPa and *E* = ~8 GPa; see Table 1; Gorb & Krings, 2021). However, here the neighboring teeth could interlock when loaded, leading to stress redistribution when in contact with the ingesta. This mechanical behavior of radula teeth is prospered by the arrangement and geometry of teeth, the water‐content and the material properties, which enables the bending capacity (Herrera et al., 2015; Hickman, 1980, 1984; Krings, Brütt, et al., 2020; Krings & Gorb, 2021; Krings, Karabacak, & Gorb, 2021; Krings, Kovalev, & Gorb, 2021b, 2021c; Krings, Marcé‐Nogué, et al., 2020; Krings, Marcé‐Nogué, & Gorb, 2021; Montroni et al., 2019; Morris & Hickman, 1981; Padilla, 2003; Solem, 1972; Ukmar‐Godec et al., 2015).

**TABLE 1:** Results from previous mechanical property studies in comparison to the results from *Gastropteron rubrum*.

Taxon	Young's modulus	Hardness	Origin of mechanical properties	References
Polyplacophora	30–130 GPa	4–12 GPa	High inorganic content	Weaver et al. (2010), Grunenfelder et al. (2014), Krings et al. (2022c)
*Patella vulgata*	52–150 GPa	3–7 GPa	High inorganic content	Lu and Barber (2012), Barber et al. (2015)
*Megathura crenulata*	16 GPa	–	Cross‐linking	Ukmar‐Godec et al. (2017)
Nudibranchia (*Felimare picta*, *Doris pseudoargus*)	15 GPa (inner structure), 45 GPa (leading edge)	0.9 GPa (inner structure), 2.3 GPa (leading edge)	Probably cross‐linking (inner structure), high inorganic content (leading edges)	Krings et al. (2023)
Paludomid gastropods	4.1–9.2 GPa	0.10–0.50 GPa	Probably cross‐linking	Gorb and Krings (2021)
*Gastropteron rubrum*	1.60–15.95 GPa	0.10–0.85 GPa	Potentially cross‐linking	

*Note*: Here, the Young's modulus (*E*) and the hardness (*H*) values and their origins are listed.

In some solid substrate feeders (i.e., the nudibranch gastropods *Felimare picta* and *Doris pseudoargus*), the different teeth of each row had similar mechanical properties. Here, teeth probably also had similar functions (“monofunctional radula”; Krings et al., 2023). However, in other taxa, there were pronounced gradients within each transversal tooth row present, i.e., different tooth types had different mechanical properties. For example, in some paludomid gastropods, the central teeth were the stiffest and hardest elements, followed by the lateral, and finally the marginal teeth (Gorb & Krings, 2021; Krings et al., 2019, 2022d). The central and lateral teeth are probably capable of loosening algae from rocks, whereas the marginal teeth rather collect loosened food particles in a complex motion of the buccal mass afterwards. Since teeth of one row probably had different functions, this type of radula was previously termed “multifunctional radula” (see Gorb & Krings, 2021; Krings, 2020).

**FIGURE 8:**
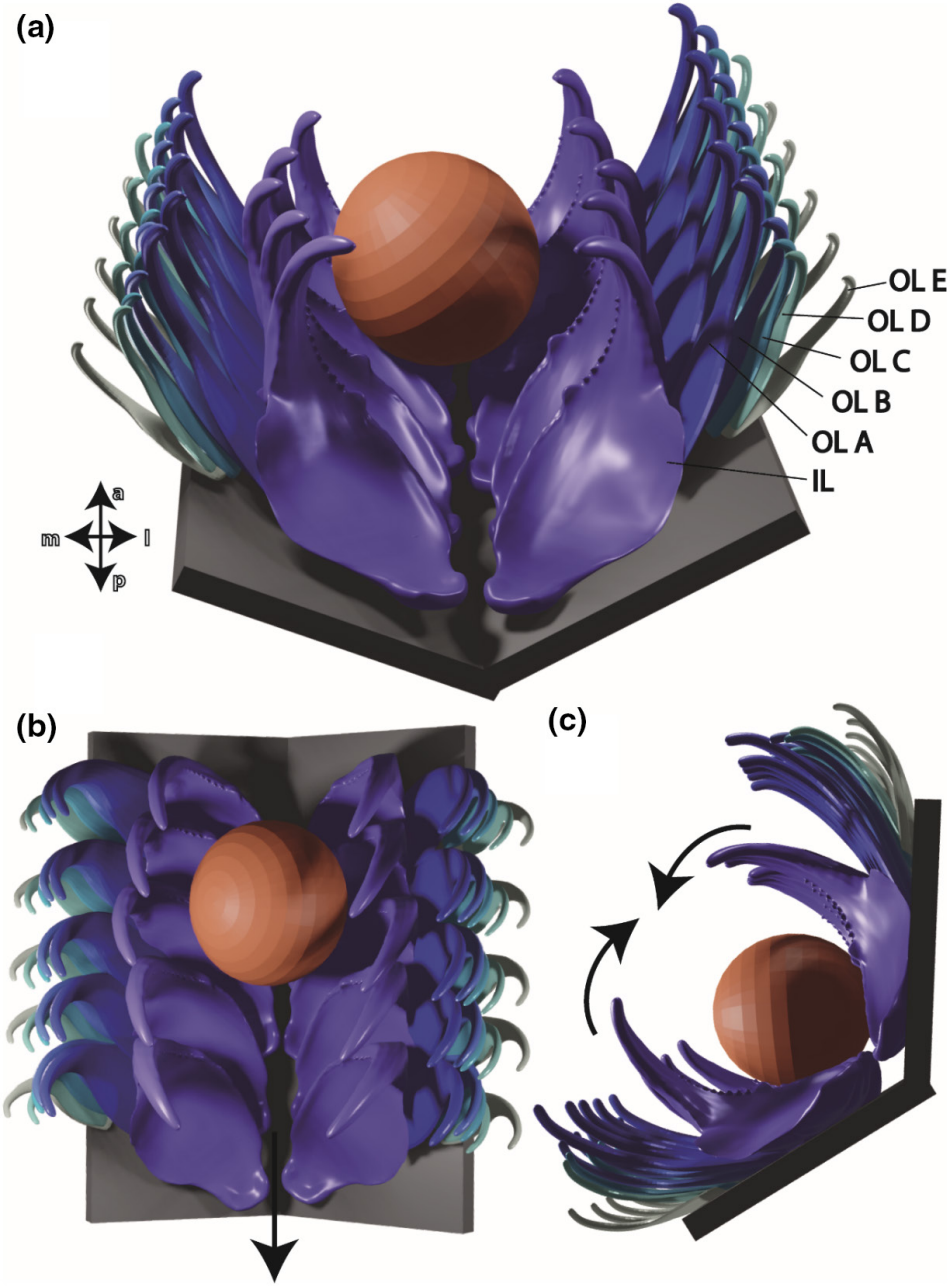
Proposed biomechanical behavior of *Gastropteron rubrum*'s radula, based on data from mechanical property tests and the documentation of the radular wear. (a) Radula 3D model from frontal view with clamped Foraminifera between inner laterals. (b) Model from above. (c) Model from the side. Arrows indicate the folding towards the radular groove, which enables the clamping of ingesta. Afterwards the radula with the particles is pulled into the mouth cavity (arrow in b). a, anterior; IL, inner lateral; l, lateral; m, medial; OL A, outer lateral A; OL B, outer lateral B; OL C, outer lateral C; OL D, outer lateral D; OL E, outer lateral E; p, posterior.

The teeth of *G. rubrum* show mechanical properties that are comparable to nudibranch teeth. In *G. rubrum*, the large inner laterals are harder and stiffer than the smaller outer laterals, with the outermost being the softest and most flexible ones. This indicates that the different teeth might experience different loads during foraging. With regard to the tested regions, we found that the bases and the bulges are most flexible and soft. This suggests that the teeth can bend in anterior–posterior direction around their bases, probably adjusting to the sizes of different prey items. Additionally, teeth are probably capable of bending in lateral‐medial direction with the bulges serving as cushions (see Figure 8). This mechanical behavior was also observed when the radula was manipulated by tweezers: the radula could be folded around the groove towards medial. As consequence, the teeth bent towards the center forming a groove. SEM documentation revealed that the degree of wear and structural failure decreased towards the radular sides. All of this suggests, that during feeding, the Foraminifera, the sand particles or Porifera parts are clamped between the inner laterals during folding along the groove (Figure 8c). This behavior of radula was previously also described for other cephalaspid taxa foraging on worms or bivalves (Hurst, 1965; Rudman, 1972a). During this, the softer and more flexible outer teeth could serve as cushions and supporting structures, which would render this radula to be multifunctional since teeth have different functions. Subsequently, the radula with the particles is probably pulled into the mouth cavity. This system would allow *G. rubrum* to take in ingesta of different sizes, since its radula could easily adapt.

The prey from the distal region of the intestine was more brittle and fractured than the prey extracted from the proximal regions. In many Cephalaspidea, a strongly muscularized gizzard with plates is located after the esophagus, which crushes the food items as shells, Foraminifera, or diatoms (Cedhagen, 1996; Eilertsen & Malaquias, 2013; Malaquias et al., 2004; Rudman, 1972a; Shepelenko et al., 2015). In Gastropteridae, however, gizzard plates were probably lost (Rudman, 1978) and were not detected in *G. rubrum* here. Besides of the prey items, we detected various sand particles in the intestine. This either indicates, that individuals of *G. rubrum* do not feed selectively, but instead probably feed on the sand surface and take everything randomly in, or that they use the sand to crush the prey items. However, the fragmentation of the prey items might also involve acidic liquids, which should be investigated in the future.

### 4.2 Wear reduction

Wear reducing mechanisms are well‐investigated in Polyplacophora and Patellogastropoda. Here, high proportions of iron and silicon are incorporated into the thick leading edges (surface layer), which are hard and protected against wear (Brooker et al., 2003; Kim et al., 1989; Kirschvink & Lowenstam, 1979; Kisailus & Nemoto, 2018; Krings et al., 2022c; Lee, Brooker, Macey, et al., 2003; Lee, Brooker, van Bronswijk, et al., 2003; Lowenstam & Weiner, 1989; Saunders et al., 2009, 2011; van der Wal, 1989; van der Wal et al., 1999; Wang et al., 2013; Weaver et al., 2010). Compared to the harder outer surface layer, the teeth possess a softer inner core, which reduces crack formation (Grunenfelder et al., 2014; van der Wal et al., 1989; van der Wal et al., 1999). In some paludomid and nudibranch taxa, we previously also identified a surface layer with high Si or Ca content at the tooth region, which interacts with the food (Krings et al., 2022a, 2023; Krings & Gorb, 2023a). This layer, however, was very thin in comparison with that of the chitons and limpets, rendering these teeth as lightweight structures. In *G. rubrum*, we detected high content of Si and Ca in the tooth regions, which interact with the ingesta. This suggests that this also reduces wear during feeding. A similar adaptation involving the incorporation of Si at the tooth tip and the presence of a soft tooth base to reduce wear by foraging on hard prey, such as diatoms, has also been found in the gnathobases of Copepoda (Michels et al., 2012; Michels & Gorb, 2015).

### 4.3 The origin of the mechanical properties

In some taxa (i.e., Polyplacophora or Patellogastropoda), high proportions of iron, silicon, and calcium are incorporated into some tooth cusps, which directly relate to mechanical property differences in the various tooth regions (for in depth reviews, see Brooker & Shaw, 2012; Faivre & Ukmar‐Godec, 2015; Joester & Brooker, 2016). In *G. rubrum*, each tooth showed mechanical property gradients in its inner structure; but we could not relate these gradients with the inorganic content.

As the radula is composed of an organic matrix of chitin fibers with associated proteins (Guralnick & Smith, 1999; Runham, 1963), the fiber architecture (i.e., fiber density, size, etc.) can promote regional mechanical property differences (Evans et al., 1990, 1994; Gordon & Joester, 2011; Grunenfelder et al., 2014; Krings, Brütt, et al., 2020, Krings, Marcé‐Nogué, et al., 2020, Krings et al., 2022a; Lu & Barber, 2012; Runham, 1963; Shaw et al., 2010; Stegbauer et al., 2021; Ukmar‐Godec, 2016; Ukmar‐Godec et al., 2017; van der Wal, 1989; van der Wal et al., 1999; Wang et al., 2013; Wealthall et al., 2005). Whether this is the case for *G. rubrum* awaits further investigations, for example, in the form of tooth section investigations in TEM.

The chitin can also show different regional degrees of tanning, which result in different mechanical properties. The degree of tanning can be visualized by applying CLSM, according to the protocol of Michels and Gorb (2012). This protocol was developed for insect cuticle, which consists of unmineralized chitin. It allowed previously the identification of cuticle regions with certain dominating material composition in insect cuticle (see Table 2): Blue signals were induced from regions containing high proportions of resilin or proteins; these regions were relatively soft and flexible. Sclerotized cuticle was associated with a red signal; this region was relatively hard and stiff. Weakly‐sclerotized chitin was related to a green signal indicating regions, which were flexible and relatively tough. When proteins were abundant, those structures appeared brown, yellow, or pink in overlay. This protocol (Michels & Gorb, 2012) was applied in many studies on arthropod cuticles (e.g., Beutel et al., 2020; Friedrich & Kubiak, 2018; Lehnert et al., 2021; Matsumura et al., 2021; Peisker et al., 2013) and cross‐validated by employing nanoindentation in lady beetles and antlions (Krings & Gorb, 2023b; Peisker et al., 2013).

**TABLE 2:** Results from previous CSLM studies in comparison to the results from *Gastropteron rubrum*.

Laser wavelength (nm)	Assigned color	Insect cuticle (Michels & Gorb, 2012)	Crustacean feeding structures (Krings et al., 2022b; Michels et al., 2012; Michels & Gorb, 2015)	Paludomid radula (*Lavigeria grandis*; Krings et al., 2022d)	Nudibranch radula (*Felimare picta* and *Doris pseudoargus*; Krings et al., 2023)	Radula of *Gastropteron rubrum*
405	Blue	Resilin, proteins, unsclerotized chitin	Calcium and proteins or probably unsclerotized chitin	Probably proteins or unsclerotized chitin	Calcium and proteins or unsclerotized chitin	Probably proteins or unsclerotized chitin
488	Green	Weakly‐sclerotized chitin	Silicon	Probably weakly‐sclerotized chitin	Silicon	Silicon
555 and 639	Red	Sclerotized chitin	Probably sclerotized chitin	Probably sclerotized chitin	Probably sclerotized chitin	Probably sclerotized chitin
405, 488, 555, 639	Brown, yellow, or pink	Proteins and sclerotized chitin in overlay	Proteins and chitin in overlay	Probably proteins and chitin in overlay	–	–

*Note*: Here, the lasers of the distinct wavelengths, their assigned colors and the materials, which emitted the strong autofluorescence signals after excitements, are listed.

The same protocol was applied by Krings et al. (2022d) to the radula of the paludomid *Lavigeria grandis*, which possesses relatively low mineral content. Here, the autofluorescence signals (see Table 2) directly related to the mechanical property values received from nanoindentation technique. We also applied this protocol for the radulae of the nudibranch gastropods *Felimare picta* and *Doris pseudoargus*, but detected that the autofluorescence signal was distorted by the content of Ca and Si in the tooth surfaces (Krings et al., 2023). Nudibranch teeth with surfaces full of Ca showed a strong blue signal, and teeth containing high Si content in the surfaces a strong green signal (see Table 2). This pattern was previously also detected in crustacean feeding structures, the gnathobases and gastric mill teeth, containing either high content of Ca or Si (see Table 2; Krings et al., 2022b; Michels et al., 2012; Michels & Gorb, 2015). In *G. rubrum*, we detected that the surfaces containing Si appeared green (see Table 2). This shows that the protocol of Michels and Gorb (2012) cannot be directly applied to mineralized structures and that EDX analyses should be included into studies. However, the tooth sides of *G. rubrum* did not contain high proportions of Ca and Si or any of the other analyzed elements, so that the protocol could be applied to these regions. Potentially teeth were here sclerotized with a decreasing degree towards the basis. The bases and bulges of *G. rubrum* teeth were unmineralized and appeared strongly blue, which indicates that either proteins or unsclerotized chitin were present in these regions, which both increase the softness and flexibility.

## AUTHOR CONTRIBUTIONS

**Wencke Krings:** Conceptualization (lead); data curation (lead); funding acquisition (lead); investigation (equal); methodology (equal); project administration (lead); supervision (lead); validation (lead); visualization (lead); writing – original draft (lead); writing – review and editing (lead). **Charlotte Neumann:** Investigation (equal); methodology (equal); validation (supporting); visualization (supporting). **Stanislav N. Gorb:** Conceptualization (supporting); methodology (supporting); project administration (supporting); resources (lead); writing – review and editing (supporting). **Alexander Koehnsen:** Investigation (supporting); methodology (supporting). **Heike Wägele:** Conceptualization (supporting); project administration (supporting); resources (supporting); supervision (supporting); writing – review and editing (equal).

## FUNDING INFORMATION

This research was financed by the Deutsche Forschungsgemeinschaft (DFG) grant 470833544 to WK.

## CONFLICT OF INTEREST STATEMENT

The authors declare that they have no competing interests.

## CONSENT FOR PUBLICATION

Not applicable.

## Data Availability

The 3D model can be found in Dryad: Wencke Krings, 3D model radula of *Gastropteron rubrum*; DOI: 10.5061/dryad.s4mw6m9bp. The data on mechanical properties and elemental analysis can be found in the Appendix 1.

## APPENDIX 1

**TABLE A1:** Results from the EDX analysis (mean and standard deviation; given in atomic %) for the inner tooth structure and the tooth surface (all tooth types and regions pooled together).

Locality	*N*	Ca		Results from Wilcoxon‐test			Cl		Results from Wilcoxon‐test			Cu		Results from Wilcoxon‐test		
		Mean	SD	χ^2^	df	*p*‐value	Mean	SD	χ^2^	df	*p*‐value	Mean	SD	χ^2^	df	*p*‐value
Inner structure	180	0.47	0.23	5.1434	1	<.0001*	0.03	0.03	141.9183	1	<.0001*	0.05	0.04	136.0744	1	<.0001*
Surface	294	2.28	2.28				0.08	0.08				0.14	0.11			

Locality	*N*	Fe		Results from Wilcoxon‐test			K		Results from Wilcoxon‐test			Mg		Results from Wilcoxon‐test		
		Mean	SD	χ^2^	df	*p*‐value	Mean	SD	χ^2^	df	*p*‐value	Mean	SD	χ^2^	df	*p*‐value
Inner structure	180	0.53	0.35	10.8848	1	.0010*	0.03	0.02	81.4326	1	<.0001*	0.26	0.09	39.7468	1	<.0001*
Surface	294	0.60	0.72				0.05	0.03				0.34	0.20			

Locality	*N*	P + Pt		Results from Wilcoxon‐test			S		Results from Wilcoxon‐test			Si		Results from Wilcoxon‐test		
		Mean	SD	χ^2^	df	*p*‐value	Mean	SD	χ^2^	df	*p*‐value	Mean	SD	χ^2^	df	*p*‐value
Inner structure	180	0.56	0.26	44.7110	1	<.0001*	0.34	0.12	239.1987	1	<.0001*	0.04	0.13	305.4376	1	<.0001*
Surface	294	0.85	0.60				0.67	0.30				5.79	6.40			

Locality	*N*	Zn		Results from Wilcoxon‐test			Ae		Results from Wilcoxon‐test							
		Mean	SD	χ^2^	df	*p*‐value	Mean	SD	χ^2^	df	*p*‐value					
Inner structure	180	0.06	0.03	117.5291	1	<.0001*	2.12	0.74	153.9240	1	<.0001*					
Surface	294	0.12	0.08				10.63	9.70								

*Note*: Results from pairwise comparison by Wilcoxon method between the inner tooth structure and the surface layer for the elemental proportions. Orange *p*‐values = highly significant differences, red *p*‐values = significant differences, black = no significant differences.

Abbreviations: df, degrees of freedom; *N*, quantity of point measurements; SD, standard deviation.

**TABLE A2:** Results from the EDX analysis (mean and standard deviation; given in atomic %).

Tooth type	Locality	*N*	Ca		Cl		Cu		Fe		K		Mg	
			Mean	SD	Mean	SD	Mean	SD	Mean	SD	Mean	SD	Mean	SD
Inner laterals	Inner structure	70	0.32	0.20	0.05	0.04	0.08	0.04	0.73	0.46	0.03	0.01	0.23	0.09
Inner laterals	Surface	134	2.60	3.01	0.08	0.07	0.15	0.11	0.80	0.90	0.05	0.03	0.31	0.17
Outer laterals A	Inner structure	22	0.60	0.20	0.01	0.01	0.02	0.01	0.41	0.18	0.03	0.02	0.29	0.09
Outer laterals A	Surface	34	1.62	2.18	0.06	0.06	0.10	0.08	0.38	0.38	0.05	0.03	0.36	0.23
Outer laterals B	Inner structure	22	0.56	0.19	0.01	0.01	0.02	0.01	0.40	0.18	0.03	0.02	0.27	0.09
Outer laterals B	Surface	34	1.99	2.66	0.08	0.09	0.13	0.11	0.43	0.37	0.06	0.04	0.36	0.22
Outer laterals C	Inner structure	22	0.56	0.19	0.01	0.01	0.02	0.01	0.40	0.18	0.03	0.02	0.27	0.09
Outer laterals C	Surface	34	2.21	2.78	0.08	0.08	0.15	0.11	0.51	0.51	0.05	0.03	0.37	0.23
Outer laterals D	Inner structure	22	0.56	0.18	0.01	0.01	0.02	0.01	0.40	0.18	0.03	0.02	0.27	0.09
Outer laterals D	Surface	32	2.21	2.78	0.07	0.08	0.09	0.10	0.52	0.59	0.06	0.03	0.39	0.22
Outer laterals E	Inner structure	22	0.56	0.19	0.01	0.01	0.02	0.01	0.40	0.18	0.03	0.02	0.28	0.09
Outer laterals E	Surface	26	1.87	2.71	0.11	0.10	0.15	0.13	0.31	0.36	0.05	0.02	0.29	0.23

Tooth type	Locality	*N*	P + Pt		S		Si		Zn		Ae	
			Mean	SD	Mean	SD	Mean	SD	Mean	SD	Mean	SD
Inner laterals	Inner structure	70	0.58	0.30	0.43	0.14	0.06	0.20	0.07	0.04	2.25	0.79
Inner laterals	Surface	134	0.85	0.64	0.69	0.30	6.63	6.80	0.11	0.07	12.01	10.34
Outer laterals A	Inner structure	22	0.55	0.24	0.30	0.07	0.03	0.02	0.05	0.02	2.09	0.72
Outer laterals A	Surface	34	0.80	0.55	0.59	0.25	4.26	5.07	0.11	0.06	8.10	7.76
Outer laterals B	Inner structure	22	0.55	0.24	0.29	0.06	0.02	0.01	0.05	0.02	2.02	0.71
Outer laterals B	Surface	34	0.99	0.64	0.70	0.32	4.81	5.53	0.13	0.08	9.41	8.51
Outer laterals C	Inner structure	22	0.55	0.24	0.29	0.07	0.02	0.02	0.05	0.02	2.03	0.71
Outer laterals C	Surface	34	0.81	0.60	0.67	0.28	5.19	5.84	0.13	0.09	9.96	9.32
Outer laterals D	Inner structure	22	0.55	0.24	0.29	0.07	0.02	0.01	0.05	0.02	2.02	0.70
Outer laterals D	Surface	32	0.85	0.54	0.66	0.32	6.09	6.57	0.11	0.08	10.82	9.99
Outer laterals E	Inner structure	22	0.55	0.25	0.28	0.06	0.02	0.01	0.05	0.02	2.03	0.71
Outer laterals E	Surface	26	0.73	0.40	0.63	0.33	5.11	7.13	0.09	0.05	9.02	9.82

*Note*: For the inner tooth structure and the tooth surface, elemental proportions are sorted according to tooth types.

Abbreviations: *N*, quantity of point measurements; SD, standard deviation.

**TABLE A3:** For the elemental proportions: results from pairwise comparison by Wilcoxon method between the inner tooth structure and the tooth surface sorted to tooth types.

Element	Structure 1	Structure 2	Results from Wilcoxon‐test			*p*‐value	Element	Structure 1	Structure 2	Results from Wilcoxon‐test			*p*‐value
			χ^2^	df	*p*‐value					χ^2^	df	*p*‐value	
Ae	IL Olayer	IL IS	163.706	11	<.0001*	<.0001*	Mg	IL Olayer	IL IS	55.1818	11	<.0001*	.0015*
Ae	OL B Olayer	IL IS				<.0001*	Mg	OL D Olayer	IL IS				.0003*
Ae	OL D Olayer	IL IS				.0002*	Mg	OL D Olayer	IL Olayer				.0531
Ae	OL C Olayer	IL IS				.0015*	Mg	OL B Olayer	IL IS				.0110*
Ae	OL E Olayer	IL IS				.0021*	Mg	OL C Olayer	IL IS				.0110*
Ae	OL B Olayer	OL B IS				.0006*	Mg	OL A IS	IL IS				.0253*
Ae	OL D Olayer	OL B IS				.0007*	Mg	OL A Olayer	IL IS				.0462*
Ae	OL B Olayer	OL A IS				.0010*	Mg	OL C IS	IL IS				.0662
Ae	OL D Olayer	OL D IS				.0007*	Mg	OL C Olayer	IL Olayer				.2015
Ae	OL A Olayer	IL IS				.0201*	Mg	OL E IS	IL IS				.0704
Ae	OL D Olayer	OL C IS				.0008*	Mg	OL D IS	IL IS				.0718
Ae	OL D Olayer	OL A IS				.0013*	Mg	OL B IS	IL IS				.0778
Ae	OL C Olayer	OL B IS				.0052*	Mg	OL B Olayer	IL Olayer				.2681
Ae	OL C Olayer	OL C IS				.0055*	Mg	OL D Olayer	OL B IS				.0620
Ae	OL E Olayer	OL C IS				.0023*	Mg	OL D Olayer	OL C IS				.0658
Ae	OL E Olayer	OL D IS				.0023*	Mg	OL D Olayer	OL D IS				.0698
Ae	OL E Olayer	OL E IS				.0023*	Mg	OL A Olayer	IL Olayer				.3982
Ae	OL E Olayer	OL B IS				.0024*	Mg	OL D Olayer	OL A IS				.1111
Ae	OL C Olayer	OL A IS				.0078*	Mg	OL C Olayer	OL B IS				.2113
Ae	OL E Olayer	OL A IS				.0044*	Mg	OL C Olayer	OL C IS				.2270
Ae	OL A Olayer	OL A IS				.0274*	Mg	OL B Olayer	OL B IS				.2436
Ae	OL D Olayer	OL A Olayer				.2430	Mg	OL C Olayer	OL A IS				.2791
Ae	OL B Olayer	OL A Olayer				.2777	Mg	OL D Olayer	OL A Olayer				.4263
Ae	OL C Olayer	OL A Olayer				.3739	Mg	OL B Olayer	OL A IS				.4109
Ae	OL E Olayer	OL A Olayer				.4468	Mg	OL D Olayer	OL B Olayer				.4724
Ae	OL D Olayer	OL C Olayer				.5944	Mg	OL C Olayer	OL B Olayer				.6677
Ae	OL D Olayer	OL B Olayer				.6861	Mg	OL A Olayer	OL A IS				.6749
Ae	OL E Olayer	OL C Olayer				.8991	Mg	OL C Olayer	OL A Olayer				.6992
Ae	OL C IS	OL B IS				.9813	Mg	OL E Olayer	IL IS				.7981
Ae	OL E IS	OL D IS				.9813	Mg	OL D Olayer	OL C Olayer				.7630
Ae	OL D IS	OL B IS				1.0000	Mg	OL D IS	OL C IS				.9813
Ae	OL E IS	OL B IS				1.0000	Mg	OL D IS	OL B IS				1.0000
Ae	OL E IS	OL C IS				1.0000	Mg	OL E IS	OL C IS				1.0000
Ae	OL D IS	OL C IS				.9439	Mg	OL C IS	OL B IS				.9813
Ae	OL C Olayer	OL B Olayer				.9072	Mg	OL E IS	OL D IS				.9813
Ae	OL E Olayer	OL B Olayer				.7826	Mg	OL E IS	OL B IS				.9532
Ae	OL E IS	OL A IS				.6641	Mg	OL B Olayer	OL A Olayer				.9511
Ae	OL C IS	OL A IS				.6472	Mg	OL C IS	OL A IS				.6303
Ae	OL B IS	OL A IS				.6304	Mg	OL A IS	IL Olayer				.8506
Ae	OL D IS	OL A IS				.6138	Mg	OL D IS	OL A IS				.5651
Ae	OL E Olayer	OL D Olayer				.6279	Mg	OL E IS	OL A IS				.5651
Ae	OL A IS	IL IS				.4612	Mg	OL B IS	OL A IS				.5185
Ae	OL D Olayer	IL Olayer				.5707	Mg	OL E Olayer	OL E IS				.5279
Ae	OL B IS	IL IS				.3031	Mg	OL E Olayer	OL B IS				.5012
Ae	OL E IS	IL IS				.2988	Mg	OL E Olayer	OL C IS				.4947
Ae	OL D IS	IL IS				.2946	Mg	OL E Olayer	OL D IS				.4817
Ae	OL C IS	IL IS				.2821	Mg	OL D IS	OL A Olayer				.5183
Ae	OL B IS	OL A Olayer				.0176*	Mg	OL B IS	OL A Olayer				.4915
Ae	OL C IS	OL A Olayer				.0176*	Mg	OL C IS	OL A Olayer				.4758
Ae	OL D IS	OL A Olayer				.0176*	Mg	OL E IS	OL A Olayer				.4654
Ae	OL E IS	OL A Olayer				.0176*	Mg	OL E Olayer	OL A IS				.3359
Ae	OL E Olayer	IL Olayer				.2843	Mg	OL E Olayer	OL A Olayer				.2929
Ae	OL B Olayer	IL Olayer				.2506	Mg	OL D IS	OL B Olayer				.2645
Ae	OL C Olayer	IL Olayer				.1988	Mg	OL C IS	OL B Olayer				.2369
Ae	OL E IS	OL C Olayer				.0055*	Mg	OL D IS	OL C Olayer				.2369
Ae	OL D IS	OL C Olayer				.0050*	Mg	OL E IS	OL B Olayer				.2303
Ae	OL E IS	OL D Olayer				.0008*	Mg	OL E IS	OL C Olayer				.2270
Ae	OL D IS	OL B Olayer				.0006*	Mg	OL C IS	IL Olayer				.5789
Ae	OL E IS	OL B Olayer				.0006*	Mg	OL E IS	IL Olayer				.5771
Ae	OL C IS	OL B Olayer				.0005*	Mg	OL B IS	IL Olayer				.5616
Ae	OL A Olayer	IL Olayer				.0200*	Mg	OL E Olayer	OL B Olayer				.1770
Ae	OL A IS	IL Olayer				<.0001*	Mg	OL D IS	IL Olayer				.5479
Ae	OL C IS	IL Olayer				<.0001*	Mg	OL E Olayer	OL C Olayer				.1088
Ae	OL E IS	IL Olayer				<.0001*	Mg	OL E IS	OL D Olayer				.0633
Ae	OL B IS	IL Olayer				<.0001*	Mg	OL E Olayer	OL D Olayer				.0368*
Ae	OL D IS	IL Olayer				<.0001*	Mg	OL E Olayer	IL Olayer				.2750
Ca	OL A IS	IL IS	26.1852	11	.0061	<.0001*	P + Pt	IL Olayer	IL IS	48.2629	11	<.0001*	.0023*
Ca	OL C IS	IL IS				<.0001*	P + Pt	OL B Olayer	IL IS				<.0001*
Ca	OL D IS	IL IS				<.0001*	P + Pt	OL B Olayer	IL Olayer				.0610
Ca	OL E IS	IL IS				<.0001*	P + Pt	OL D Olayer	IL IS				.0182*
Ca	OL B IS	IL IS				<.0001*	P + Pt	OL C Olayer	IL IS				.0192*
Ca	IL Olayer	IL IS				.2167	P + Pt	OL E Olayer	IL IS				.0327*
Ca	OL C IS	OL B Olayer				.3022	P + Pt	OL B Olayer	OL B IS				.0031*
Ca	OL D IS	OL B Olayer				.3022	P + Pt	OL B Olayer	OL A IS				.0042*
Ca	OL E IS	OL B Olayer				.3022	P + Pt	OL A Olayer	IL IS				.0882
Ca	OL D IS	OL A Olayer				.3264	P + Pt	OL D Olayer	OL B IS				.0561
Ca	OL B IS	OL A Olayer				.3347	P + Pt	OL D Olayer	OL C IS				.0561
Ca	OL C IS	OL A Olayer				.3347	P + Pt	OL D Olayer	OL D IS				.0561
Ca	OL E IS	OL A Olayer				.3347	P + Pt	OL D Olayer	OL A IS				.0671
Ca	OL D IS	OL C Olayer				.4655	P + Pt	OL C Olayer	OL B IS				.0984
Ca	OL E IS	OL C Olayer				.4655	P + Pt	OL B Olayer	OL A Olayer				.1283
Ca	OL A Olayer	IL IS				.6599	P + Pt	OL C Olayer	OL C IS				.1091
Ca	OL E IS	OL D Olayer				.5792	P + Pt	OL C Olayer	OL A IS				.1128
Ca	OL D Olayer	IL IS				.7592	P + Pt	OL E Olayer	OL D IS				.1688
Ca	OL E Olayer	OL C Olayer				.7484	P + Pt	OL A Olayer	OL A IS				.2113
Ca	OL A IS	IL Olayer				.9048	P + Pt	OL E Olayer	OL B IS				.1820
Ca	OL C IS	IL Olayer				.9371	P + Pt	OL E Olayer	OL C IS				.1889
Ca	OL E IS	IL Olayer				.9371	P + Pt	OL D Olayer	IL Olayer				.5735
Ca	OL B IS	IL Olayer				.9411	P + Pt	OL E Olayer	OL E IS				.2032
Ca	OL D IS	IL Olayer				.9411	P + Pt	OL E Olayer	OL A IS				.2300
Ca	OL E Olayer	OL D Olayer				.9253	P + Pt	OL D Olayer	OL A Olayer				.5768
Ca	OL E Olayer	OL B Olayer				.9584	P + Pt	OL D Olayer	OL C Olayer				.5856
Ca	OL E IS	OL D IS				.9625	P + Pt	OL C Olayer	IL Olayer				.9104
Ca	OL E IS	OL C IS				.9719	P + Pt	OL E Olayer	IL Olayer				.9226
Ca	OL D Olayer	OL C Olayer				.9949	P + Pt	OL C Olayer	OL A Olayer				.9316
Ca	OL D IS	OL B IS				1.0000	P + Pt	OL E IS	OL D IS				.9719
Ca	OL D IS	OL C IS				1.0000	P + Pt	OL E Olayer	OL A Olayer				.9821
Ca	OL E IS	OL B IS				1.0000	P + Pt	OL E IS	OL B IS				.9813
Ca	OL D Olayer	OL A Olayer				.9795	P + Pt	OL E IS	OL A IS				.9906
Ca	OL D Olayer	OL B Olayer				.9795	P + Pt	OL C IS	OL A IS				1.0000
Ca	OL C IS	OL B IS				.9625	P + Pt	OL C IS	OL B IS				1.0000
Ca	OL C Olayer	OL B Olayer				.9072	P + Pt	OL D IS	OL B IS				1.0000
Ca	OL C Olayer	OL A Olayer				.8492	P + Pt	OL E IS	OL C IS				1.0000
Ca	OL B Olayer	OL A Olayer				.7267	P + Pt	OL D IS	OL C IS				.9906
Ca	OL E Olayer	OL A Olayer				.7036	P + Pt	OL B IS	OL A IS				.9719
Ca	OL B Olayer	IL IS				.7790	P + Pt	OL D IS	OL A IS				.9719
Ca	OL C Olayer	IL IS				.7368	P + Pt	OL E Olayer	OL C Olayer				.9405
Ca	OL D Olayer	OL B IS				.6035	P + Pt	OL A IS	IL IS				.8691
Ca	OL D Olayer	OL C IS				.6035	P + Pt	OL A Olayer	IL Olayer				.8917
Ca	OL D Olayer	OL D IS				.6035	P + Pt	OL E IS	IL IS				.8368
Ca	OL D Olayer	OL A IS				.5672	P + Pt	OL C IS	IL IS				.8225
Ca	OL C IS	OL A IS				.4886	P + Pt	OL D IS	IL IS				.8225
Ca	OL E IS	OL A IS				.4455	P + Pt	OL B IS	IL IS				.8083
Ca	OL D IS	OL A IS				.4316	P + Pt	OL E Olayer	OL D Olayer				.4866
Ca	OL B IS	OL A IS				.4046	P + Pt	OL D Olayer	OL B Olayer				.3556
Ca	OL C Olayer	OL A IS				.4655	P + Pt	OL E IS	OL A Olayer				.2303
Ca	OL C Olayer	OL B IS				.4655	P + Pt	OL C IS	OL A Olayer				.2239
Ca	OL C Olayer	OL C IS				.4655	P + Pt	OL B IS	OL A Olayer				.2114
Ca	OL A Olayer	OL A IS				.3181	P + Pt	OL D IS	OL A Olayer				.1994
Ca	OL B Olayer	OL B IS				.3022	P + Pt	OL E IS	OL C Olayer				.1129
Ca	OL B Olayer	OL A IS				.2645	P + Pt	OL E Olayer	OL B Olayer				.1121
Ca	OL E Olayer	IL IS				.3451	P + Pt	OL D IS	OL C Olayer				.0984
Ca	OL E Olayer	OL B IS				.0769	P + Pt	OL C Olayer	OL B Olayer				.1207
Ca	OL E Olayer	OL D IS				.0769	P + Pt	OL E IS	OL D Olayer				.0561
Ca	OL A Olayer	IL Olayer				.4391	P + Pt	OL E IS	OL B Olayer				.0034*
Ca	OL E Olayer	OL A IS				.0735	P + Pt	OL C IS	OL B Olayer				.0031*
Ca	OL E Olayer	OL C IS				.0735	P + Pt	OL D IS	OL B Olayer				.0027*
Ca	OL E Olayer	OL E IS				.0735	P + Pt	OL A IS	IL Olayer				.0816
Ca	OL E Olayer	IL Olayer				.3883	P + Pt	OL E IS	IL Olayer				.0794
Ca	OL D Olayer	IL Olayer				.3209	P + Pt	OL B IS	IL Olayer				.0751
Ca	OL B Olayer	IL Olayer				.2838	P + Pt	OL C IS	IL Olayer				.0743
Ca	OL C Olayer	IL Olayer				.2631	P + Pt	OL D IS	IL Olayer				.0743
Cl	IL Olayer	IL IS	195.6244	11	<.0001*	.0067*	S	IL Olayer	IL IS	264.2958	11	<.0001*	<.0001*
Cl	OL A Olayer	OL A IS				<.0001*	S	OL B Olayer	IL IS				<.0001*
Cl	OL E Olayer	OL A IS				<.0001*	S	OL C Olayer	IL IS				<.0001*
Cl	OL E Olayer	OL B IS				<.0001*	S	OL D Olayer	IL IS				.0002*
Cl	OL E Olayer	OL C IS				<.0001*	S	OL C Olayer	OL C IS				<.0001*
Cl	OL E Olayer	OL D IS				<.0001*	S	OL C Olayer	OL B IS				<.0001*
Cl	OL E Olayer	OL E IS				<.0001*	S	OL B Olayer	OL B IS				<.0001*
Cl	OL D Olayer	OL A IS				<.0001*	S	OL C Olayer	OL A IS				<.0001*
Cl	OL C Olayer	OL C IS				<.0001*	S	OL B Olayer	OL A IS				<.0001*
Cl	OL D Olayer	OL C IS				<.0001*	S	OL A Olayer	IL IS				.0007*
Cl	OL C Olayer	OL A IS				<.0001*	S	OL D Olayer	OL B IS				<.0001*
Cl	OL C Olayer	OL B IS				<.0001*	S	OL D Olayer	OL C IS				<.0001*
Cl	OL D Olayer	OL B IS				<.0001*	S	OL A Olayer	OL A IS				<.0001*
Cl	OL D Olayer	OL D IS				<.0001*	S	OL D Olayer	OL D IS				<.0001*
Cl	OL B Olayer	OL A IS				<.0001*	S	OL D Olayer	OL A IS				<.0001*
Cl	OL E Olayer	IL IS				.0037*	S	OL E Olayer	IL IS				.0042*
Cl	OL B Olayer	OL B IS				<.0001*	S	OL E Olayer	OL E IS				.0003*
Cl	OL E Olayer	IL Olayer				.1135	S	OL E Olayer	OL C IS				.0003*
Cl	OL E Olayer	OL A Olayer				.0224*	S	OL E Olayer	OL B IS				.0003*
Cl	OL C Olayer	IL IS				.1940	S	OL E Olayer	OL D IS				.0003*
Cl	OL E Olayer	OL D Olayer				.0758	S	OL E Olayer	OL A IS				.0006*
Cl	OL B Olayer	IL IS				.2131	S	OL B Olayer	OL A Olayer				.1602
Cl	OL D Olayer	IL IS				.2934	S	OL C Olayer	OL A Olayer				.2199
Cl	OL E Olayer	OL B Olayer				.2155	S	OL D Olayer	OL A Olayer				.4685
Cl	OL E Olayer	OL C Olayer				.2265	S	OL E Olayer	OL A Olayer				.5657
Cl	OL B Olayer	OL A Olayer				.4144	S	OL B Olayer	IL Olayer				.8761
Cl	OL A Olayer	IL IS				.5425	S	OL E IS	OL C IS				.8879
Cl	OL C Olayer	OL A Olayer				.4390	S	OL D IS	OL C IS				.9906
Cl	OL D Olayer	OL A Olayer				.8122	S	OL E IS	OL D IS				.9906
Cl	OL D IS	OL C IS				.9528	S	OL D IS	OL B IS				1.0000
Cl	OL D IS	OL B IS				.9716	S	OL E IS	OL B IS				.9532
Cl	OL E IS	OL C IS				.9430	S	OL C IS	OL B IS				.9158
Cl	OL C IS	OL B IS				.9337	S	OL C Olayer	OL B Olayer				.9170
Cl	OL E IS	OL D IS				.9337	S	OL E Olayer	OL D Olayer				.8328
Cl	OL C Olayer	OL B Olayer				.9169	S	OL C Olayer	IL Olayer				.8948
Cl	OL E IS	OL B IS				.8862	S	OL D Olayer	OL C Olayer				.7242
Cl	OL D IS	OL A IS				.8217	S	OL E Olayer	OL C Olayer				.6926
Cl	OL C IS	OL A IS				.6781	S	OL E Olayer	OL B Olayer				.6438
Cl	OL B IS	OL A IS				.6435	S	OL D Olayer	OL B Olayer				.6350
Cl	OL E IS	OL A IS				.5447	S	OL D IS	OL A IS				.5338
Cl	OL D Olayer	OL B Olayer				.6031	S	OL B IS	OL A IS				.5184
Cl	OL D Olayer	OL C Olayer				.5545	S	OL E IS	OL A IS				.5033
Cl	OL B Olayer	IL Olayer				.7104	S	OL C IS	OL A IS				.4595
Cl	OL C Olayer	IL Olayer				.6987	S	OL D Olayer	IL Olayer				.6031
Cl	OL D Olayer	IL Olayer				.4091	S	OL E Olayer	IL Olayer				.5098
Cl	OL A Olayer	IL Olayer				.1488	S	OL A Olayer	IL Olayer				.1059
Cl	OL D IS	OL B Olayer				<.0001*	S	OL E IS	OL D Olayer				<.0001*
Cl	OL C IS	OL B Olayer				<.0001*	S	OL D IS	OL A Olayer				<.0001*
Cl	OL E IS	OL B Olayer				<.0001*	S	OL C IS	OL A Olayer				<.0001*
Cl	OL D IS	OL C Olayer				<.0001*	S	OL E IS	OL A Olayer				<.0001*
Cl	OL B IS	OL A Olayer				<.0001*	S	OL B IS	OL A Olayer				<.0001*
Cl	OL D IS	OL A Olayer				<.0001*	S	OL D IS	OL B Olayer				<.0001*
Cl	OL E IS	OL D Olayer				<.0001*	S	OL E IS	OL B Olayer				<.0001*
Cl	OL E IS	OL C Olayer				<.0001*	S	OL C IS	OL B Olayer				<.0001*
Cl	OL C IS	OL A Olayer				<.0001*	S	OL D IS	OL C Olayer				<.0001*
Cl	OL E IS	OL A Olayer				<.0001*	S	OL E IS	OL C Olayer				<.0001*
Cl	OL A IS	IL IS				<.0001*	S	OL A IS	IL IS				<.0001*
Cl	OL D IS	IL IS				<.0001*	S	OL D IS	IL IS				<.0001*
Cl	OL B IS	IL IS				<.0001*	S	OL C IS	IL IS				<.0001*
Cl	OL C IS	IL IS				<.0001*	S	OL B IS	IL IS				<.0001*
Cl	OL E IS	IL IS				<.0001*	S	OL E IS	IL IS				<.0001*
Cl	OL B IS	IL Olayer				<.0001*	S	OL A IS	IL Olayer				<.0001*
Cl	OL D IS	IL Olayer				<.0001*	S	OL B IS	IL Olayer				<.0001*
Cl	OL C IS	IL Olayer				<.0001*	S	OL D IS	IL Olayer				<.0001*
Cl	OL A IS	IL Olayer				<.0001*	S	OL C IS	IL Olayer				<.0001*
Cl	OL E IS	IL Olayer				<.0001*	S	OL E IS	IL Olayer				<.0001*
Cu	IL Olayer	IL IS	209.6904	11	<.0001*	<.0001*	Si	IL Olayer	IL IS	310.6357	11	<.0001*	<.0001*
Cu	OL C Olayer	OL A IS				<.0001*	Si	OL C Olayer	IL IS				<.0001*
Cu	OL C Olayer	OL B IS				<.0001*	Si	OL B Olayer	IL IS				<.0001*
Cu	OL C Olayer	OL C IS				<.0001*	Si	OL A Olayer	IL IS				<.0001*
Cu	OL B Olayer	OL B IS				<.0001*	Si	OL D Olayer	IL IS				<.0001*
Cu	OL B Olayer	OL A IS				<.0001*	Si	OL E Olayer	IL IS				<.0001*
Cu	OL A Olayer	OL A IS				<.0001*	Si	OL A Olayer	OL A IS				<.0001*
Cu	OL E Olayer	OL D IS				<.0001*	Si	OL B Olayer	OL A IS				<.0001*
Cu	OL E Olayer	OL A IS				<.0001*	Si	OL B Olayer	OL B IS				<.0001*
Cu	OL E Olayer	OL B IS				<.0001*	Si	OL C Olayer	OL A IS				<.0001*
Cu	OL E Olayer	OL E IS				<.0001*	Si	OL C Olayer	OL B IS				<.0001*
Cu	OL E Olayer	OL C IS				<.0001*	Si	OL C Olayer	OL C IS				<.0001*
Cu	OL C Olayer	IL IS				.0027*	Si	OL D Olayer	OL A IS				<.0001*
Cu	OL D Olayer	OL D IS				<.0001*	Si	OL D Olayer	OL B IS				<.0001*
Cu	OL D Olayer	OL B IS				<.0001*	Si	OL D Olayer	OL C IS				<.0001*
Cu	OL D Olayer	OL C IS				<.0001*	Si	OL D Olayer	OL D IS				<.0001*
Cu	OL D Olayer	OL A IS				<.0001*	Si	OL E Olayer	OL A IS				<.0001*
Cu	OL C Olayer	OL A Olayer				.0201*	Si	OL E Olayer	OL B IS				<.0001*
Cu	OL E Olayer	IL IS				.0852	Si	OL E Olayer	OL C IS				<.0001*
Cu	OL E Olayer	OL D Olayer				.0638	Si	OL E Olayer	OL D IS				<.0001*
Cu	OL E Olayer	OL A Olayer				.1087	Si	OL E Olayer	OL E IS				<.0001*
Cu	OL B Olayer	IL IS				.2915	Si	OL C Olayer	OL B Olayer				.0229*
Cu	OL C Olayer	OL B Olayer				.2886	Si	OL D Olayer	OL B Olayer				.0235*
Cu	OL B Olayer	OL A Olayer				.3770	Si	OL D Olayer	OL A Olayer				.0628
Cu	OL E Olayer	OL B Olayer				.4831	Si	OL C Olayer	OL A Olayer				.0826
Cu	OL D IS	OL C IS				.7677	Si	OL D Olayer	OL C Olayer				.1160
Cu	OL E IS	OL C IS				.9056	Si	OL B Olayer	OL A Olayer				.1298
Cu	OL D IS	OL B IS				.9718	Si	OL E Olayer	OL B Olayer				.1500
Cu	OL E IS	OL D IS				.9155	Si	OL D Olayer	IL Olayer				.5960
Cu	OL C Olayer	IL Olayer				.9496	Si	OL E Olayer	OL A Olayer				.2964
Cu	OL E IS	OL B IS				.8502	Si	OL E Olayer	OL C Olayer				.4292
Cu	OL A Olayer	IL IS				.9034	Si	OL D IS	OL B IS				.9154
Cu	OL C IS	OL B IS				.7145	Si	OL C IS	OL B IS				.9436
Cu	OL E Olayer	OL C Olayer				.7036	Si	OL E IS	OL B IS				.9624
Cu	OL D Olayer	OL A Olayer				.6077	Si	OL D IS	OL C IS				1.0000
Cu	OL E IS	OL A IS				.5110	Si	OL E IS	OL C IS				1.0000
Cu	OL B IS	OL A IS				.3967	Si	OL E IS	OL D IS				.9718
Cu	OL E Olayer	IL Olayer				.6338	Si	OL A IS	IL IS				.9434
Cu	OL D Olayer	OL B Olayer				.2302	Si	OL E Olayer	IL Olayer				.9465
Cu	OL D Olayer	IL IS				.3459	Si	OL E Olayer	OL D Olayer				.6672
Cu	OL D IS	OL A IS				.1070	Si	OL B IS	OL A IS				.2246
Cu	OL C IS	OL A IS				.0967	Si	OL C IS	OL A IS				.2247
Cu	OL B Olayer	IL Olayer				.1795	Si	OL D IS	OL A IS				.2244
Cu	OL D Olayer	OL C Olayer				.0076*	Si	OL E IS	OL A IS				.2246
Cu	OL E IS	OL D Olayer				<.0001*	Si	OL C Olayer	IL Olayer				.5009
Cu	OL E IS	OL A Olayer				<.0001*	Si	OL B IS	IL IS				.1110
Cu	OL C IS	OL A Olayer				<.0001*	Si	OL C IS	IL IS				.1110
Cu	OL B IS	OL A Olayer				<.0001*	Si	OL D IS	IL IS				.1110
Cu	OL D IS	OL A Olayer				<.0001*	Si	OL E IS	IL IS				.1110
Cu	OL C IS	OL B Olayer				<.0001*	Si	OL B Olayer	IL Olayer				.0249*
Cu	OL E IS	OL B Olayer				<.0001*	Si	OL A Olayer	IL Olayer				.0098*
Cu	OL D IS	OL B Olayer				<.0001*	Si	OL E IS	OL D Olayer				<.0001*
Cu	OL E IS	OL C Olayer				<.0001*	Si	OL B IS	OL A Olayer				<.0001*
Cu	OL D IS	OL C Olayer				<.0001*	Si	OL C IS	OL A Olayer				<.0001*
Cu	OL A Olayer	IL Olayer				.0046*	Si	OL C IS	OL B Olayer				<.0001*
Cu	OL D Olayer	IL Olayer				.0013*	Si	OL D IS	OL A Olayer				<.0001*
Cu	OL A IS	IL IS				<.0001*	Si	OL D IS	OL B Olayer				<.0001*
Cu	OL E IS	IL IS				<.0001*	Si	OL D IS	OL C Olayer				<.0001*
Cu	OL B IS	IL IS				<.0001*	Si	OL E IS	OL A Olayer				<.0001*
Cu	OL C IS	IL IS				<.0001*	Si	OL E IS	OL B Olayer				<.0001*
Cu	OL D IS	IL IS				<.0001*	Si	OL E IS	OL C Olayer				<.0001*
Cu	OL C IS	IL Olayer				<.0001*	Si	OL A IS	IL Olayer				<.0001*
Cu	OL E IS	IL Olayer				<.0001*	Si	OL B IS	IL Olayer				<.0001*
Cu	OL A IS	IL Olayer				<.0001*	Si	OL C IS	IL Olayer				<.0001*
Cu	OL B IS	IL Olayer				<.0001*	Si	OL D IS	IL Olayer				<.0001*
Cu	OL D IS	IL Olayer				<.0001*	Si	OL E IS	IL Olayer				<.0001*
Fe	OL C IS	OL A Olayer	38.1268	11	<.0001*	.1019	Zn	IL Olayer	IL IS	127.1231	11	<.0001*	.0003*
Fe	OL E IS	OL A Olayer				.1019	Zn	OL D Olayer	IL IS				<.0001*
Fe	OL B IS	OL A Olayer				.1129	Zn	OL B Olayer	IL IS				<.0001*
Fe	OL D IS	OL A Olayer				.1129	Zn	OL A Olayer	IL IS				.0004*
Fe	OL C Olayer	OL A Olayer				.2565	Zn	OL B Olayer	OL B IS				<.0001*
Fe	OL B Olayer	OL A Olayer				.2777	Zn	OL D Olayer	OL B IS				<.0001*
Fe	OL C IS	OL B Olayer				.4655	Zn	OL B Olayer	OL A IS				<.0001*
Fe	OL E IS	OL B Olayer				.4863	Zn	OL D Olayer	OL A IS				<.0001*
Fe	OL E IS	OL D Olayer				.4868	Zn	OL D Olayer	OL D IS				<.0001*
Fe	OL D IS	OL B Olayer				.5183	Zn	OL D Olayer	OL C IS				<.0001*
Fe	OL D IS	OL C Olayer				.6810	Zn	OL A Olayer	OL A IS				<.0001*
Fe	OL E IS	OL C Olayer				.6810	Zn	OL C Olayer	IL IS				.0054*
Fe	OL D Olayer	OL A Olayer				.7051	Zn	OL D Olayer	IL Olayer				.1266
Fe	OL C Olayer	OL B Olayer				.9072	Zn	OL B Olayer	IL Olayer				.1402
Fe	OL D IS	OL C IS				.9532	Zn	OL E Olayer	IL IS				.0496*
Fe	OL D IS	OL B IS				1.0000	Zn	OL C Olayer	OL B IS				.0105*
Fe	OL E IS	OL C IS				.9813	Zn	OL C Olayer	OL C IS				.0105*
Fe	OL C IS	OL B IS				.9625	Zn	OL C Olayer	OL A IS				.0124*
Fe	OL E IS	OL B IS				.9345	Zn	OL E Olayer	OL B IS				.0142*
Fe	OL E IS	OL D IS				.9158	Zn	OL E Olayer	OL C IS				.0150*
Fe	OL E Olayer	OL A Olayer				.9228	Zn	OL E Olayer	OL D IS				.0159*
Fe	OL E IS	OL A IS				.8973	Zn	OL E Olayer	OL E IS				.0168*
Fe	OL C IS	OL A IS				.8602	Zn	OL E Olayer	OL A IS				.0240*
Fe	OL B IS	OL A IS				.8418	Zn	OL C Olayer	IL Olayer				.4883
Fe	OL D IS	OL A IS				.8053	Zn	OL A Olayer	IL Olayer				.5804
Fe	OL D Olayer	OL B Olayer				.7338	Zn	OL D Olayer	OL A Olayer				.4042
Fe	OL C Olayer	OL B IS				.7058	Zn	OL B Olayer	OL A Olayer				.4882
Fe	OL A IS	IL Olayer				.8546	Zn	OL C Olayer	OL A Olayer				.6766
Fe	OL C Olayer	OL C IS				.6445	Zn	OL D Olayer	OL C Olayer				.6765
Fe	OL E Olayer	OL D Olayer				.6334	Zn	OL D IS	OL C IS				.9251
Fe	OL C Olayer	OL A IS				.5856	Zn	OL D Olayer	OL B Olayer				.9488
Fe	OL D Olayer	OL C Olayer				.5811	Zn	OL D IS	OL B IS				.9812
Fe	OL D Olayer	OL B IS				.5320	Zn	OL E IS	OL C IS				.9812
Fe	OL C IS	IL Olayer				.7775	Zn	OL E IS	OL B IS				1.0000
Fe	OL B Olayer	OL B IS				.5075	Zn	OL E IS	OL D IS				.9812
Fe	OL D Olayer	OL D IS				.4868	Zn	OL E IS	OL A IS				.9718
Fe	OL D IS	IL Olayer				.7697	Zn	OL C IS	OL B IS				.9625
Fe	OL E IS	IL Olayer				.7542	Zn	OL C IS	OL A IS				.7501
Fe	OL D Olayer	OL C IS				.4543	Zn	OL C Olayer	OL B Olayer				.7544
Fe	OL B IS	IL Olayer				.7503	Zn	OL B IS	OL A IS				.6798
Fe	OL D Olayer	OL A IS				.4438	Zn	OL D IS	OL A IS				.4864
Fe	OL B Olayer	OL A IS				.4452	Zn	OL E Olayer	OL A Olayer				.3067
Fe	OL E Olayer	OL B Olayer				.1794	Zn	OL E Olayer	OL C Olayer				.2385
Fe	OL E Olayer	OL C Olayer				.1746	Zn	OL E Olayer	IL Olayer				.4905
Fe	OL A Olayer	OL A IS				.0886	Zn	OL A IS	IL IS				.2791
Fe	OL E Olayer	OL B IS				.0106*	Zn	OL E Olayer	OL D Olayer				.1072
Fe	OL E Olayer	OL D IS				.0106*	Zn	OL E IS	IL IS				.2647
Fe	OL E Olayer	OL E IS				.0100*	Zn	OL C IS	IL IS				.2204
Fe	OL E Olayer	OL C IS				.0094*	Zn	OL E Olayer	OL B Olayer				.0722
Fe	OL E Olayer	OL A IS				.0074*	Zn	OL B IS	IL IS				.2069
Fe	OL C Olayer	IL Olayer				.2129	Zn	OL D IS	IL IS				.1816
Fe	OL B Olayer	IL Olayer				.1213	Zn	OL E IS	OL C Olayer				.0105*
Fe	IL Olayer	IL IS				.0376*	Zn	OL D IS	OL C Olayer				.0095*
Fe	OL D Olayer	IL Olayer				.0544	Zn	OL E IS	OL A Olayer				<.0001*
Fe	OL D Olayer	IL IS				.0035*	Zn	OL C IS	OL A Olayer				<.0001*
Fe	OL C Olayer	IL IS				.0030*	Zn	OL D IS	OL A Olayer				<.0001*
Fe	OL A IS	IL IS				.0026*	Zn	OL B IS	OL A Olayer				<.0001*
Fe	OL D IS	IL IS				.0020*	Zn	OL E IS	OL D Olayer				<.0001*
Fe	OL B IS	IL IS				.0019*	Zn	OL D IS	OL B Olayer				<.0001*
Fe	OL E IS	IL IS				.0018*	Zn	OL E IS	OL B Olayer				<.0001*
Fe	OL C IS	IL IS				.0018*	Zn	OL C IS	OL B Olayer				<.0001*
Fe	OL B Olayer	IL IS				.0004*	Zn	OL A IS	IL Olayer				.0014*
Fe	OL E Olayer	IL Olayer				.0140*	Zn	OL E IS	IL Olayer				.0011*
Fe	OL A Olayer	IL Olayer				.0083*	Zn	OL C IS	IL Olayer				.0011*
Fe	OL A Olayer	IL IS				<.0001*	Zn	OL B IS	IL Olayer				.0009*
Fe	OL E Olayer	IL IS				<.0001*	Zn	OL D IS	IL Olayer				.0008*
K	IL Olayer	IL IS	85.9342	11	<.0001*	<.0001*	K	OL E Olayer	OL C Olayer				.7426
K	OL A Olayer	IL IS				<.0001*	K	OL D IS	OL A IS				.8229
K	OL D Olayer	IL IS				<.0001*	K	OL E Olayer	OL A Olayer				.8696
K	OL B Olayer	IL IS				.0002*	K	OL D IS	OL B IS				.8597
K	OL E Olayer	IL IS				.0008*	K	OL D IS	OL C IS				.9250
K	OL C Olayer	IL IS				.0025*	K	OL C IS	OL B IS				.9625
K	OL B Olayer	OL B IS				.0297*	K	OL E IS	OL B IS				.9718
K	OL B Olayer	OL A IS				.0333*	K	OL E IS	OL C IS				1.0000
K	OL D Olayer	OL A IS				.0334*	K	OL B IS	OL A IS				.9343
K	OL D Olayer	OL B IS				.0338*	K	OL D Olayer	OL B Olayer				.9284
K	OL D Olayer	OL C IS				.0353*	K	OL E IS	OL D IS				.9063
K	OL D Olayer	OL D IS				.0402*	K	OL C IS	OL A IS				.8969
K	OL D IS	IL IS				.1698	K	OL E IS	OL A IS				.7865
K	OL A Olayer	OL A IS				.0500*	K	OL C Olayer	IL Olayer				.8947
K	OL B Olayer	IL Olayer				.3542	K	OL C Olayer	OL A Olayer				.7871
K	OL E Olayer	OL A IS				.0450*	K	OL E Olayer	OL D Olayer				.7366
K	OL E Olayer	OL B IS				.0457*	K	OL E Olayer	OL B Olayer				.4971
K	OL E Olayer	OL D IS				.0457*	K	OL C Olayer	OL B Olayer				.3605
K	OL E Olayer	OL E IS				.0480*	K	OL D IS	OL C Olayer				.1053
K	OL A IS	IL IS				.2085	K	OL E IS	OL C Olayer				.0885
K	OL D Olayer	IL Olayer				.3998	K	OL D IS	OL A Olayer				.0613
K	OL E Olayer	OL C IS				.0504	K	OL B IS	OL A Olayer				.0526
K	OL B IS	IL IS				.2354	K	OL C IS	OL A Olayer				.0526
K	OL C Olayer	OL A IS				.0879	K	OL E IS	OL A Olayer				.0486*
K	OL C Olayer	OL B IS				.0916	K	OL D IS	OL B Olayer				.0397*
K	OL C Olayer	OL C IS				.1018	K	OL E IS	OL D Olayer				.0338*
K	OL C IS	IL IS				.2994	K	OL C IS	OL B Olayer				.0352*
K	OL E IS	IL IS				.4481	K	OL E IS	OL B Olayer				.0297*
K	OL D Olayer	OL C Olayer				.3897	K	OL D IS	IL Olayer				.0330*
K	OL B Olayer	OL A Olayer				.5274	K	OL C IS	IL Olayer				.0226*
K	OL E Olayer	IL Olayer				.7794	K	OL B IS	IL Olayer				.0208*
K	OL D Olayer	OL A Olayer				.6210	K	OL A IS	IL Olayer				.0202*
K	OL A Olayer	IL Olayer				.8126	K	OL E IS	IL Olayer				.0175*

*Note*: Orange *p*‐values = highly significant differences, red *p*‐values = significant differences, black = no significant differences.

Abbreviations: *N*, quantity of point measurements; IL, inner lateral tooth; IS, inner structure; OL, outer lateral tooth; Olayer, outer layer.

**TABLE A4:** Results from the EDX analysis (mean and standard deviation; given in atomic %).

Locality	Tooth region	*N*	Ca		Cl		Cu		Fe	
			Mean	SD	Mean	SD	Mean	SD	Mean	SD
Surface	Bulge	46	0.05	0.05	0.02	0.02	0.08	0.08	0.09	0.07
Surface	Basis	46	0.08	0.07	0.06	0.05	0.15	0.12	0.14	0.09
Surface	Stylus, basis	46	3.41	2.41	0.09	0.08	0.12	0.08	1.00	0.81
Surface	Stylus, terminal	46	5.32	1.72	0.12	0.11	0.13	0.08	1.12	0.68
Surface	Cusp	46	5.56	2.01	0.10	0.08	0.13	0.07	1.26	0.75
Surface	Sides	64	0.10	0.09	0.08	0.06	0.19	0.14	0.17	0.10
Inner structure	Whole tooth area	180	0.47	0.23	0.03	0.03	0.05	0.04	0.53	0.35

Locality	Locality	*N*	K		Mg		Na		P + Pt	
			Mean	SD	Mean	SD	Mean	SD	Mean	SD
Surface	Bulge	46	0.02	0.01	0.13	0.04	0.00	0.00	0.49	0.38
Surface	Basis	46	0.05	0.03	0.23	0.08	0.00	0.00	0.91	0.67
Surface	Stylus, basis	46	0.06	0.03	0.52	0.21	0.00	0.00	1.05	0.70
Surface	Stylus, terminal	46	0.06	0.03	0.50	0.20	0.00	0.00	0.81	0.41
Surface	Cusp	46	0.05	0.03	0.44	0.17	0.00	0.00	0.80	0.44
Surface	Sides	64	0.06	0.03	0.25	0.08	0.00	0.00	0.97	0.69
Inner structure	Whole tooth area	180	0.03	0.02	0.26	0.09	0.00	0.00	0.56	0.26

Locality	Locality	*N*	S		Si		Zn		Ae	
			Mean	SD	Mean	SD	Mean	SD	Mean	SD
Surface	Bulge	46	0.26	0.10	0.27	0.14	0.04	0.02	1.37	0.49
Surface	Basis	46	0.67	0.22	0.35	0.16	0.10	0.05	2.45	0.85
Surface	Stylus, basis	46	0.66	0.27	8.00	1.77	0.14	0.08	14.74	3.80
Surface	Stylus, terminal	46	0.85	0.25	11.58	1.26	0.14	0.08	20.31	2.61
Surface	Cusp	46	0.76	0.26	16.24	2.76	0.15	0.08	25.17	4.04
Surface	Sides	64	0.78	0.27	0.38	0.19	0.11	0.06	2.76	0.87
Inner structure	Whole tooth area	180	0.34	0.12	0.04	0.13	0.06	0.03	2.12	0.74

*Note*: For the inner tooth structure and the tooth surface (all tooth types pooled together), elemental proportions are sorted according to regions.

Abbreviations: *N*, quantity of point measurements; SD, standard deviation.

**TABLE A5:** Results from the EDX analysis (mean and standard deviation; given in atomic %).

Tooth type	Locality	Locality	*N*	Ca		Cl		Cu		Fe		K		Mg	
				Mean	SD	Mean	SD	Mean	SD	Mean	SD	Mean	SD	Mean	SD
Inner laterals	Surface	Bulge	23	0.05	0.03	0.03	0.02	0.09	0.08	0.11	0.07	0.02	0.01	0.14	0.04
Inner laterals	Surface	Basis	23	0.07	0.05	0.07	0.06	0.17	0.13	0.16	0.09	0.06	0.03	0.24	0.07
Inner laterals	Surface	Stylus, basis	23	3.53	2.60	0.08	0.07	0.13	0.09	1.17	1.06	0.06	0.03	0.49	0.21
Inner laterals	Surface	Stylus, terminal	23	5.59	1.81	0.11	0.08	0.15	0.09	1.49	0.77	0.05	0.02	0.38	0.16
Inner laterals	Surface	Cusp	23	5.85	2.12	0.10	0.07	0.16	0.06	1.59	0.77	0.05	0.02	0.36	0.10
Inner laterals	Surface	Sides	19	0.08	0.06	0.08	0.06	0.22	0.14	0.20	0.10	0.06	0.03	0.26	0.08
Inner laterals	Inner structure	Whole tooth area	70	0.32	0.20	0.05	0.04	0.08	0.04	0.73	0.46	0.03	0.01	0.23	0.09
Outer laterals A	Surface	Bulge	5	0.11	0.09	0.02	0.01	0.06	0.05	0.09	0.07	0.02	0.01	0.10	0.02
Outer laterals A	Surface	Basis	5	0.16	0.14	0.04	0.02	0.12	0.11	0.12	0.09	0.05	0.01	0.18	0.01
Outer laterals A	Surface	Stylus, basis	5	2.42	2.07	0.08	0.06	0.13	0.07	0.63	0.50	0.06	0.03	0.51	0.11
Outer laterals A	Surface	Stylus, terminal	5	4.00	2.57	0.12	0.11	0.09	0.06	0.92	0.22	0.06	0.03	0.57	0.16
Outer laterals A	Surface	Cusp	5	4.11	1.46	0.07	0.06	0.09	0.07	0.62	0.12	0.04	0.02	0.66	0.18
Outer laterals A	Surface	Sides	9	0.14	0.14	0.04	0.02	0.09	0.10	0.11	0.08	0.07	0.02	0.24	0.07
Outer laterals A	Inner structure	Whole tooth area	22	0.60	0.20	0.01	0.01	0.02	0.01	0.41	0.18	0.03	0.02	0.29	0.09
Outer laterals B	Surface	Bulge	5	0.03	0.02	0.01	0.01	0.08	0.11	0.10	0.09	0.02	0.01	00.12	0.04
Outer laterals B	Surface	Basis	5	0.05	0.03	0.03	0.02	0.13	0.14	0.15	0.12	0.04	0.03	0.22	0.09
Outer laterals B	Surface	Stylus, basis	5	2.90	2.74	0.09	0.08	0.09	0.09	0.84	0.31	0.08	0.03	0.54	0.12
Outer laterals B	Surface	Stylus, terminal	5	5.52	1.51	0.20	0.15	0.14	0.07	0.73	0.17	0.07	0.02	0.63	0.26
Outer laterals B	Surface	Cusp	5	4.90	1.98	0.09	0.10	0.12	0.10	0.76	0.36	0.05	0.06	0.50	0.07
Outer laterals B	Surface	Sides	9	0.07	0.04	0.06	0.03	0.17	0.12	0.19	0.08	0.07	0.03	0.26	0.08
Outer laterals B	Inner structure	Whole tooth area	22	0.56	0.19	0.01	0.01	0.02	0.01	0.40	0.18	0.03	0.02	0.27	0.09
Outer laterals C	Surface	Bulge	5	0.03	0.02	0.02	0.02	0.10	0.07	0.10	0.07	0.01	0.01	0.10	0.04
Outer laterals C	Surface	Basis	5	0.05	0.03	0.04	0.04	0.17	0.10	0.14	0.09	0.02	0.02	0.17	0.07
Outer laterals C	Surface	Stylus, basis	5	4.75	1.84	0.09	0.09	0.13	0.06	0.94	0.35	0.08	0.03	0.59	0.07
Outer laterals C	Surface	Stylus, terminal	5	4.83	0.90	0.14	0.14	0.11	0.10	0.67	0.17	0.05	0.02	0.66	0.11
Outer laterals C	Surface	Cusp	5	5.80	2.29	0.06	0.03	0.10	0.05	1.27	0.59	0.06	0.02	0.54	0.12
Outer laterals C	Surface	Sides	9	0.08	0.06	0.10	0.09	0.24	0.15	0.19	0.12	0.04	0.02	0.26	0.08
Outer laterals C	Inner structure	Whole tooth area	22	0.56	0.19	0.01	0.01	0.02	0.01	0.40	0.18	0.03	0.02	0.27	0.09
Outer laterals D	Surface	Bulge	5	0.03	0.02	0.02	0.01	0.05	0.06	0.06	0.06	0.02	0.02	0.16	0.07
Outer laterals D	Surface	Basis	5	0.05	0.03	0.04	0.03	0.07	0.06	0.08	0.06	0.04	0.03	0.27	0.07
Outer laterals D	Surface	Stylus, basis	5	2.57	1.93	0.09	0.11	0.06	0.05	0.94	0.29	0.06	0.03	0.61	0.21
Outer laterals D	Surface	Stylus, terminal	5	5.34	1.27	0.15	0.13	0.14	0.10	0.67	0.25	0.08	0.04	0.58	0.20
Outer laterals D	Surface	Cusp	5	5.99	2.26	0.08	0.04	0.09	0.05	1.37	0.73	0.06	0.03	0.54	0.12
Outer laterals D	Surface	Sides	7	0.13	0.13	0.05	0.03	0.13	0.18	0.14	0.12	0.06	0.03	0.25	0.08
Outer laterals D	Inner structure	Whole tooth area	22	0.56	0.18	0.01	0.01	0.02	0.01	0.40	0.18	0.03	0.02	0.27	0.09
Outer laterals E	Surface	Bulge	3	0.04	0.03	0.03	0.01	0.07	0.07	0.11	0.05	0.02	0.01	0.14	0.03
Outer laterals E	Surface	Basis	3	0.06	0.05	0.08	0.03	0.13	0.11	0.14	0.04	0.04	0.03	0.26	0.08
Outer laterals E	Surface	Stylus, basis	3	4.12	2.91	0.13	0.12	0.14	0.10	0.83	0.73	0.06	0.03	0.53	0.53
Outer laterals E	Surface	Stylus, terminal	3	5.79	1.43	0.06	0.06	0.05	0.02	0.68	0.30	0.03	0.03	0.58	0.18
Outer laterals E	Surface	Cusp	3	5.75	0.97	0.25	0.12	0.13	0.05	0.38	0.20	0.07	0.03	0.18	0.21
Outer laterals E	Surface	Sides	11	0.12	0.10	0.10	0.08	0.20	0.16	0.16	0.12	0.05	0.02	0.23	0.06
Outer laterals E	Inner structure	Whole tooth area	22	0.56	0.19	0.01	0.01	0.02	0.01	0.40	0.18	0.03	0.02	0.28	0.09

Tooth type	Locality	Locality	*N*	Na		P + Pt		S		Si		Zn		Ae	
				Mean	SD	Mean	SD	Mean	SD	Mean	SD	Mean	SD	Mean	SD
Inner laterals	Surface	Bulge	23	0.00	0.00	0.51	0.39	0.29	0.11	0.30	0.15	0.05	0.02	1.48	0.47
Inner laterals	Surface	Basis	23	0.00	0.00	0.93	0.66	0.72	0.23	0.37	0.17	0.11	0.05	2.61	0.80
Inner laterals	Surface	Stylus, basis	23	0.00	0.00	1.15	0.83	0.67	0.31	8.40	1.83	0.15	0.08	15.50	3.92
Inner laterals	Surface	Stylus, terminal	23	0.00	0.00	0.76	0.44	0.86	0.20	12.19	1.17	0.11	0.08	21.38	2.58
Inner laterals	Surface	Cusp	23	0.00	0.00	0.75	0.50	0.82	0.23	17.10	2.79	0.13	0.08	26.61	3.93
Inner laterals	Surface	Sides	19	0.00	0.00	1.04	0.77	0.83	0.28	0.34	0.14	0.12	0.06	2.89	0.90
Inner laterals	Inner structure	Whole tooth area	70	0.00	0.00	0.58	0.30	0.43	0.14	0.06	0.20	0.07	0.04	2.25	0.79
Outer laterals A	Surface	Bulge	5	0.00	0.00	0.41	0.33	0.22	0.09	0.12	0.02	0.04	0.02	1.08	0.41
Outer laterals A	Surface	Basis	5	0.00	0.00	0.80	0.67	0.58	0.23	0.15	0.02	0.09	0.04	2.02	0.80
Outer laterals A	Surface	Stylus, basis	5	0.00	0.00	0.71	0.67	0.56	0.15	5.71	2.83	0.11	0.05	10.69	5.42
Outer laterals A	Surface	Stylus, terminal	5	0.00	0.00	0.87	0.37	0.80	0.22	9.54	0.09	0.17	0.09	16.86	2.41
Outer laterals A	Surface	Cusp	5	0.00	0.00	0.95	0.29	0.72	0.13	12.77	0.18	0.15	0.06	19.90	1.69
Outer laterals A	Surface	Sides	9	0.00	0.00	0.93	0.68	0.63	0.23	0.40	0.29	0.11	0.04	2.50	0.93
Outer laterals A	Inner structure	Whole tooth area	22	0.00	0.00	0.55	0.24	0.30	0.07	0.03	0.02	0.05	0.02	2.09	0.72
Outer laterals B	Surface	Bulge	5	0.00	0.00	0.75	0.61	0.26	0.11	0.21	0.04	0.06	0.03	1.54	0.85
Outer laterals B	Surface	Basis	5	0.00	0.00	1.34	1.16	0.65	0.24	0.26	0.04	0.13	0.07	2.70	1.53
Outer laterals B	Surface	Stylus, basis	5	0.00	0.00	0.97	0.33	0.70	0.26	7.51	0.24	0.14	0.10	13.59	3.01
Outer laterals B	Surface	Stylus, terminal	5	0.00	0.00	0.95	0.57	1.00	0.30	10.40	0.33	0.20	0.05	19.49	1.75
Outer laterals B	Surface	Cusp	5	0.00	0.00	0.76	0.30	0.78	0.38	13.94	0.26	0.18	0.13	21.77	2.56
Outer laterals B	Surface	Sides	9	0.00	0.00	1.10	0.63	0.77	0.18	0.22	0.07	0.10	0.05	2.72	0.67
Outer laterals B	Inner structure	Whole tooth area	22	0.00	0.00	0.55	0.24	0.29	0.06	0.02	0.01	0.05	0.02	2.02	0.71
Outer laterals C	Surface	Bulge	5	0.00	0.00	0.28	0.11	0.24	0.10	0.26	0.04	0.03	0.03	1.06	0.19
Outer laterals C	Surface	Basis	5	0.00	0.00	0.58	0.19	0.64	0.24	0.34	0.02	0.07	0.06	1.91	0.35
Outer laterals C	Surface	Stylus, basis	5	0.00	0.00	0.96	0.56	0.68	0.20	7.94	0.15	0.18	0.06	16.04	2.31
Outer laterals C	Surface	Stylus, terminal	5	0.00	0.00	0.90	0.22	0.79	0.21	11.13	0.16	0.16	0.10	19.16	0.80
Outer laterals C	Surface	Cusp	5	0.00	0.00	0.96	0.51	0.75	0.09	14.86	0.46	0.18	0.09	24.26	3.11
Outer laterals C	Surface	Sides	9	0.00	0.00	1.00	0.93	0.82	0.29	0.43	0.07	0.14	0.09	2.95	1.10
Outer laterals C	Inner structure	Whole tooth area	22	0.00	0.00	0.55	0.24	0.29	0.07	0.02	0.02	0.05	0.02	2.03	0.71
Outer laterals D	Surface	Bulge	5	0.00	0.00	0.49	0.28	0.23	0.06	0.38	0.06	0.04	0.02	1.41	0.43
Outer laterals D	Surface	Basis	5	0.00	0.00	0.80	0.37	0.57	0.14	0.47	0.05	0.10	0.04	2.30	0.58
Outer laterals D	Surface	Stylus, basis	5	0.00	0.00	1.04	0.63	0.71	0.24	8.43	0.18	0.15	0.09	14.39	1.31
Outer laterals D	Surface	Stylus, terminal	5	0.00	0.00	0.74	0.44	0.91	0.46	11.92	0.36	0.21	0.09	20.39	1.91
Outer laterals D	Surface	Cusp	5	0.00	0.00	0.89	0.52	0.81	0.10	17.03	0.79	0.17	0.10	26.72	2.89
Outer laterals D	Surface	Sides	7	0.00	0.00	1.06	0.76	0.71	0.30	0.52	0.27	0.12	0.04	2.90	0.99
Outer laterals D	Inner structure	Whole tooth area	22	0.00	0.00	0.55	0.24	0.29	0.07	0.02	0.01	0.05	0.02	2.02	0.70
Outer laterals E	Surface	Bulge	3	0.00	0.00	0.44	0.33	0.25	0.02	0.12	0.02	0.02	0.01	1.49	0.28
Outer laterals E	Surface	Basis	3	0.00	0.00	0.86	0.63	0.67	0.15	0.59	0.04	0.05	0.02	2.70	0.70
Outer laterals E	Surface	Stylus, basis	3	0.00	0.00	1.09	0.77	0.53	0.41	8.93	0.13	0.12	0.05	16.04	2.63
Outer laterals E	Surface	Stylus, terminal	3	0.00	0.00	0.89	0.18	0.67	0.31	12.46	0.01	0.09	0.03	21.07	2.24
Outer laterals E	Surface	Cusp	3	0.00	0.00	0.61	0.16	0.34	0.49	20.30	1.18	0.14	0.03	27.60	2.07
Outer laterals E	Surface	Sides	11	0.00	0.00	0.66	0.28	0.83	0.32	0.41	0.17	0.10	0.05	2.53	0.70
Outer laterals E	Inner structure	Whole tooth area	22	0.00	0.00	0.55	0.25	0.28	0.06	0.02	0.01	0.05	0.02	2.03	0.71

*Note*: For the inner tooth structure and the tooth surface, elemental proportions are sorted according to tooth types and regions.

Abbreviations: *N*, quantity of point measurements; SD, standard deviation.

**TABLE A6:** For the elemental proportions: results from pairwise comparison by Wilcoxon method between the inner tooth structure and the tooth surface (all tooth types pooled together) are sorted according to regions.

Element	Structure 1	Structure 2	Results from Wilcoxon‐test			*p*‐value	Element	Structure 1	Structure 2	Results from Wilcoxon‐test			*p*‐value
			χ^2^	df	*p*‐value					χ^2^	df	*p*‐value	
Ae	Side OL	IS	298.4635	5	<.0001*	<.0001*	Mg	Stylus, terminal OL	Basis OL	166.1782	5	<.0001*	<.0001*
Ae	Stylus, terminal OL	Basis OL				<.0001*	Mg	Stylus, basis OL	Basis OL				<.0001*
Ae	Cusp OL	Basis OL				<.0001*	Mg	Cusp OL	Basis OL				<.0001*
Ae	Stylus, basis OL	Basis OL				<.0001*	Mg	IS	Basis OL				.0276*
Ae	Cusp OL	Stylus, basis OL				<.0001*	Mg	Side OL	Basis OL				.0686
Ae	Stylus, terminal OL	Stylus, basis OL				<.0001*	Mg	Cusp OL	Stylus, terminal OL				.1907
Ae	Cusp OL	Stylus, terminal OL				<.0001*	Mg	Side OL	IS				.4387
Ae	Side OL	Basis OL				.0264*	Mg	Stylus, terminal OL	Stylus, basis OL				.1430
Ae	IS	Basis OL				.0227*	Mg	Cusp OL	Stylus, basis OL				.0101*
Ae	Bulge OL	Basis OL				<.0001*	Mg	Bulge OL	Basis OL				<.0001*
Ae	Bulge OL	Stylus, basis OL				<.0001*	Mg	Bulge OL	Stylus, basis OL				<.0001*
Ae	Bulge OL	Stylus, terminal OL				<.0001*	Mg	Bulge OL	Cusp OL				<.0001*
Ae	Bulge OL	Cusp OL				<.0001*	Mg	Side OL	Cusp OL				<.0001*
Ae	Bulge OL	Side OL				<.0001*	Mg	Side OL	Stylus, terminal OL				<.0001*
Ae	Side OL	Stylus, basis OL				<.0001*	Mg	Bulge OL	Stylus, terminal OL				<.0001*
Ae	Side OL	Stylus, terminal OL				<.0001*	Mg	Side OL	Stylus, basis OL				<.0001*
Ae	Side OL	Cusp OL				<.0001*	Mg	Bulge OL	Side OL				<.0001*
Ae	Bulge OL	IS				<.0001*	Mg	IS	Cusp OL				<.0001*
Ae	IS	Stylus, basis OL				<.0001*	Mg	IS	Stylus, terminal OL				<.0001*
Ae	IS	Stylus, terminal OL				<.0001*	Mg	IS	Stylus, basis OL				<.0001*
Ae	IS	Cusp OL				<.0001*	Mg	Bulge OL	IS				<.0001*
Ca	IS	Basis OL	339.5842	5	<.0001*	<.0001*	P + Pt	Side OL	IS	47.0579	5	<.0001*	<.0001*
Ca	Stylus, terminal OL	Basis OL				<.0001*	P + Pt	Side OL	Cusp OL				.2152
Ca	Cusp OL	Basis OL				<.0001*	P + Pt	Stylus, basis OL	Basis OL				.2658
Ca	Stylus, basis OL	Basis OL				<.0001*	P + Pt	Side OL	Basis OL				.4506
Ca	Cusp OL	Stylus, basis OL				.0002*	P + Pt	Stylus, terminal OL	Basis OL				.7518
Ca	Stylus, terminal OL	Stylus, basis OL				.0002*	P + Pt	Side OL	Stylus, terminal OL				.7968
Ca	Side OL	Basis OL				.0312*	P + Pt	Cusp OL	Basis OL				.5819
Ca	Cusp OL	Stylus, terminal OL				.9222	P + Pt	Cusp OL	Stylus, terminal OL				.4234
Ca	Bulge OL	Basis OL				.0065*	P + Pt	Side OL	Stylus, basis OL				.4506
Ca	Bulge OL	Side OL				<.0001*	P + Pt	Stylus, terminal OL	Stylus, basis OL				.2494
Ca	Bulge OL	Stylus, basis OL				<.0001*	P + Pt	Cusp OL	Stylus, basis OL				.0947
Ca	Bulge OL	Stylus, terminal OL				<.0001*	P + Pt	Bulge OL	Basis OL				<.0001*
Ca	Bulge OL	Cusp OL				<.0001*	P + Pt	Bulge OL	Cusp OL				<.0001*
Ca	Side OL	Stylus, basis OL				<.0001*	P + Pt	Bulge OL	Stylus, terminal OL				<.0001*
Ca	Side OL	Stylus, terminal OL				<.0001*	P + Pt	Bulge OL	Stylus, basis OL				<.0001*
Ca	Side OL	Cusp OL				<.0001*	P + Pt	Bulge OL	Side OL				<.0001*
Ca	IS	Stylus, basis OL				<.0001*	P + Pt	Bulge OL	IS				.0036*
Ca	Side OL	IS				<.0001*	P + Pt	IS	Cusp OL				.0008*
Ca	Bulge OL	IS				<.0001*	P + Pt	IS	Basis OL				.0003*
Ca	IS	Stylus, terminal OL				<.0001*	P + Pt	IS	Stylus, terminal OL				<.0001*
Ca	IS	Cusp OL				<.0001*	P + Pt	IS	Stylus, basis OL				<.0001*
Cl	Side OL	IS	151.7305	5	<.0001*	<.0001*	S	Side OL	IS	250.0864	5	<.0001*	<.0001*
Cl	Stylus, terminal OL	Basis OL				.0005*	S	Stylus, terminal OL	Stylus, basis OL				.0002*
Cl	Cusp OL	Basis OL				.0045*	S	Stylus, terminal OL	Basis OL				.0003*
Cl	Side OL	Basis OL				.0142*	S	Side OL	Basis OL				.0147*
Cl	Stylus, terminal OL	Stylus, basis OL				.0318*	S	Cusp OL	Stylus, basis OL				.0101*
Cl	Stylus, basis OL	Basis OL				.1245	S	Cusp OL	Basis OL				.0126*
Cl	Cusp OL	Stylus, basis OL				.3055	S	Side OL	Stylus, basis OL				.0420*
Cl	Side OL	Stylus, basis OL				.6958	S	Stylus, basis OL	Basis OL				.8178
Cl	Cusp OL	Stylus, terminal OL				.3247	S	Side OL	Cusp OL				.5245
Cl	Side OL	Cusp OL				.1651	S	Cusp OL	Stylus, terminal OL				.2044
Cl	Side OL	Stylus, terminal OL				.0300*	S	Side OL	Stylus, terminal OL				.0820
Cl	Bulge OL	IS				.1582	S	Bulge OL	Stylus, basis OL				<.0001*
Cl	Bulge OL	Basis OL				<.0001*	S	Bulge OL	Cusp OL				<.0001*
Cl	Bulge OL	Stylus, basis OL				<.0001*	S	Bulge OL	Basis OL				<.0001*
Cl	Bulge OL	Cusp OL				<.0001*	S	Bulge OL	Stylus, terminal OL				<.0001*
Cl	Bulge OL	Stylus, terminal OL				<.0001*	S	Bulge OL	IS				<.0001*
Cl	Bulge OL	Side OL				<.0001*	S	Bulge OL	Side OL				<.0001*
Cl	IS	Basis OL				<.0001*	S	IS	Stylus, basis OL				<.0001*
Cl	IS	Stylus, basis OL				<.0001*	S	IS	Basis OL				<.0001*
Cl	IS	Cusp OL				<.0001*	S	IS	Cusp OL				<.0001*
Cl	IS	Stylus, terminal OL				<.0001*	S	IS	Stylus, terminal OL				<.0001*
Cu	Side OL	IS	139.2310	5	<.0001*	<.0001*	Si	Side OL	IS	381.5313	5	<.0001*	<.0001*
Cu	Bulge OL	IS				.0165*	Si	Bulge OL	IS				<.0001*
Cu	Side OL	Stylus, basis OL				.0211*	Si	Stylus, terminal OL	Basis OL				<.0001*
Cu	Side OL	Basis OL				.0723	Si	Cusp OL	Basis OL				<.0001*
Cu	Side OL	Stylus, terminal OL				.1967	Si	Cusp OL	Stylus, basis OL				<.0001*
Cu	Side OL	Cusp OL				.3232	Si	Stylus, basis OL	Basis OL				<.0001*
Cu	Stylus, terminal OL	Stylus, basis OL				.3307	Si	Stylus, terminal OL	Stylus, basis OL				<.0001*
Cu	Cusp OL	Stylus, basis OL				.3546	Si	Cusp OL	Stylus, terminal OL				<.0001*
Cu	Cusp OL	Basis OL				.8056	Si	Side OL	Basis OL				.5148
Cu	Cusp OL	Stylus, terminal OL				.8238	Si	Bulge OL	Basis OL				.0360*
Cu	Stylus, terminal OL	Basis OL				.9969	Si	Bulge OL	Side OL				.0106*
Cu	Stylus, basis OL	Basis OL				.4509	Si	Bulge OL	Stylus, basis OL				<.0001*
Cu	Bulge OL	Stylus, basis OL				.0197*	Si	Bulge OL	Stylus, terminal OL				<.0001*
Cu	Bulge OL	Basis OL				.0065*	Si	Bulge OL	Cusp OL				<.0001*
Cu	Bulge OL	Stylus, terminal OL				.0011*	Si	Side OL	Stylus, basis OL				<.0001*
Cu	Bulge OL	Cusp OL				.0003*	Si	Side OL	Stylus, terminal OL				<.0001*
Cu	Bulge OL	Side OL				<.0001*	Si	Side OL	Cusp OL				<.0001*
Cu	IS	Basis OL				<.0001*	Si	IS	Basis OL				<.0001*
Cu	IS	Stylus, basis OL				<.0001*	Si	IS	Stylus, basis OL				<.0001*
Cu	IS	Stylus, terminal OL				<.0001*	Si	IS	Stylus, terminal OL				<.0001*
Cu	IS	Cusp OL				<.0001*	Si	IS	Cusp OL				<.0001*
Fe	IS	Basis OL	252.1298	5	<.0001*	<.0001*	Zn	Side OL	IS	127.5303	5	<.0001*	<.0001*
Fe	Stylus, terminal OL	Basis OL				<.0001*	Zn	Stylus, basis OL	Basis OL				.0045*
Fe	Cusp OL	Basis OL				<.0001*	Zn	Cusp OL	Basis OL				.0048*
Fe	Stylus, basis OL	Basis OL				<.0001*	Zn	Stylus, terminal OL	Basis OL				.0082*
Fe	Cusp OL	Stylus, basis OL				.0411*	Zn	Side OL	Basis OL				.0891
Fe	Side OL	Basis OL				.1680	Zn	Cusp OL	Stylus, terminal OL				.7665
Fe	Stylus, terminal OL	Stylus, basis OL				.1742	Zn	Cusp OL	Stylus, basis OL				.8056
Fe	Cusp OL	Stylus, terminal OL				.3775	Zn	Stylus, terminal OL	Stylus, basis OL				.9906
Fe	Bulge OL	Basis OL				.0189*	Zn	Side OL	Stylus, terminal OL				.1526
Fe	Bulge OL	Side OL				.0003*	Zn	Side OL	Stylus, basis OL				.0820
Fe	Bulge OL	Stylus, basis OL				<.0001*	Zn	Side OL	Cusp OL				.0615
Fe	Bulge OL	Cusp OL				<.0001*	Zn	Bulge OL	Basis OL				<.0001*
Fe	Bulge OL	Stylus, terminal OL				<.0001*	Zn	Bulge OL	Stylus, terminal OL				<.0001*
Fe	IS	Stylus, basis OL				<.0001*	Zn	Bulge OL	Cusp OL				<.0001*
Fe	Side OL	Stylus, basis OL				<.0001*	Zn	Bulge OL	Stylus, basis OL				<.0001*
Fe	Side OL	Cusp OL				<.0001*	Zn	Bulge OL	Side OL				<.0001*
Fe	Side OL	Stylus, terminal OL				<.0001*	Zn	Bulge OL	IS				<.0001*
Fe	IS	Stylus, terminal OL				<.0001*	Zn	IS	Basis OL				<.0001*
Fe	IS	Cusp OL				<.0001*	Zn	IS	Stylus, terminal OL				<.0001*
Fe	Side OL	IS				<.0001*	Zn	IS	Cusp OL				<.0001*
Fe	Bulge OL	IS				<.0001*	Zn	IS	Stylus, basis OL				<.0001*
K	Side OL	IS	88.4850	5	<.0001*	<.0001*	K	Bulge OL	Basis OL				<.0001*
K	Stylus, basis OL	Basis OL				.0186*	K	Bulge OL	Stylus, terminal OL				<.0001*
K	Side OL	Basis OL				.0520	K	Bulge OL	Cusp OL				<.0001*
K	Stylus, terminal OL	Basis OL				.1520	K	IS	Basis OL				.0007*
K	Cusp OL	Basis OL				.3125	K	Bulge OL	Stylus, basis OL				<.0001*
K	Side OL	Cusp OL				.4875	K	Bulge OL	Side OL				<.0001*
K	Side OL	Stylus, terminal OL				.8533	K	Bulge OL	IS				<.0001*
K	Cusp OL	Stylus, terminal OL				.7159	K	IS	Cusp OL				<.0001*
K	Stylus, terminal OL	Stylus, basis OL				.3958	K	IS	Stylus, terminal OL				<.0001*
K	Side OL	Stylus, basis OL				.4253	K	IS	Stylus, basis OL				<.0001*
K	Cusp OL	Stylus, basis OL				.1531							

*Note*: Orange *p*‐values = highly significant differences, red *p*‐values = significant differences, black = no significant differences.

Abbreviation: df, degrees of freedom; IS, inner structure; OL, outer layer.

**TABLE A7:** Results from nanoindentation experiments (mean and standard deviation).

Tooth type	Locality	*N*	Young's modulus		Hardness	
			Mean	SD	Mean	SD
Inner laterals	Bulge	24	1.84	0.42	0.10	0.02
Inner laterals	Basis	24	4.23	0.68	0.23	0.04
Inner laterals	Stylus, basis	24	12.09	0.89	0.64	0.05
Inner laterals	Stylus, terminal	23	14.18	0.41	0.77	0.04
Inner laterals	Cusp	23	15.95	0.88	0.85	0.05
Outer laterals A	Bulge	11	1.60	0.49	0.10	0.03
Outer laterals A	Basis	11	3.96	0.71	0.21	0.04
Outer laterals A	Stylus, basis	11	11.44	0.81	0.61	0.06
Outer laterals A	Stylus, terminal	13	12.81	0.53	0.70	0.04
Outer laterals A	Cusp	13	15.55	1.61	0.88	0.14
Outer laterals B	Bulge	11	2.17	0.73	0.12	0.04
Outer laterals B	Basis	11	3.76	0.68	0.20	0.04
Outer laterals B	Stylus, basis	11	10.81	0.80	0.58	0.06
Outer laterals B	Stylus, terminal	13	12.15	0.66	0.67	0.05
Outer laterals B	Cusp	13	14.69	1.55	0.83	0.13
Outer laterals C	Bulge	11	2.12	0.60	0.11	0.03
Outer laterals C	Basis	11	3.36	0.61	0.18	0.03
Outer laterals C	Stylus, basis	11	9.67	0.72	0.52	0.05
Outer laterals C	Stylus, terminal	13	10.87	0.62	0.60	0.04
Outer laterals C	Cusp	13	13.13	1.40	0.74	0.12
Outer laterals D	Bulge	11	1.98	0.17	0.11	0.01
Outer laterals D	Basis	11	3.03	0.57	0.16	0.03
Outer laterals D	Stylus, basis	11	8.69	0.70	0.46	0.05
Outer laterals D	Stylus, terminal	13	9.75	0.65	0.54	0.04
Outer laterals D	Cusp	13	11.77	1.26	0.67	0.10
Outer laterals E	Bulge	11	1.75	0.49	0.09	0.03
Outer laterals E	Basis	11	2.64	0.49	0.14	0.03
Outer laterals E	Stylus, basis	11	7.58	0.60	0.41	0.04
Outer laterals E	Stylus, terminal	13	8.51	0.56	0.47	0.04
Outer laterals E	Cusp	13	10.27	1.10	0.58	0.09

*Note*: For each tooth type, the hardness and Young's modulus (both given in GPa) from different regions (basis, stylus, and cusp) of the inner tooth structure are given.

Abbreviations: *N*, quantity of point measurements; SD, standard deviation.

**TABLE A8:** For the mechanical property hardness (*H*) and Young's modulus (*E*): results from Kruskal–Wallis test and pairwise comparison by Wilcoxon method between the regions.

Parameter	Structure 1	Structure 2	Results from Wilcoxon‐test			*p*‐value	Parameter	Structure 1	Structure 2	Results from Wilcoxon‐test			*p*‐value
			χ^2^	df	*p*‐value					χ^2^	df	*p*‐value	
*H*	IL Stylus, basis	IL Basis	386.8802	29	<.0001*	<.0001*	*E*	IL Stylus, basis	IL Basis	386.8802	29	<.0001*	<.0001*
*H*	IL Stylus, basis	IL Bulge				<.0001*	*E*	IL Stylus, basis	IL Bulge				<.0001*
*H*	IL Stylus, term	IL Basis				<.0001*	*E*	IL Stylus, term	IL Basis				<.0001*
*H*	IL Stylus, term	IL Bulge				<.0001*	*E*	IL Stylus, term	IL Bulge				<.0001*
*H*	IL Cusp	IL Basis				<.0001*	*E*	IL Stylus, term	IL Stylus, basis				<.0001*
*H*	IL Cusp	IL Bulge				<.0001*	*E*	IL Cusp	IL Basis				<.0001*
*H*	IL Cusp	IL Stylus, basis				<.0001*	*E*	IL Cusp	IL Bulge				<.0001*
*H*	IL Stylus, term	IL Stylus, basis				<.0001*	*E*	IL Cusp	IL Stylus, basis				<.0001*
*H*	OL A Stylus, term	IL Basis				<.0001*	*E*	IL Cusp	IL Stylus, term				<.0001*
*H*	OL A Stylus, term	IL Bulge				<.0001*	*E*	OL A Stylus, term	IL Basis				<.0001*
*H*	OL A Cusp	IL Basis				<.0001*	*E*	OL A Stylus, term	IL Bulge				<.0001*
*H*	OL A Cusp	IL Bulge				<.0001*	*E*	OL A Cusp	IL Basis				<.0001*
*H*	OL B Stylus, term	IL Basis				<.0001*	*E*	OL A Cusp	IL Bulge				<.0001*
*H*	OL B Stylus, term	IL Bulge				<.0001*	*E*	OL A Cusp	IL Stylus, basis				<.0001*
*H*	OL B Cusp	IL Basis				<.0001*	*E*	OL B Stylus, term	IL Basis				<.0001*
*H*	OL B Cusp	IL Bulge				<.0001*	*E*	OL B Stylus, term	IL Bulge				<.0001*
*H*	OL C Stylus, term	IL Basis				<.0001*	*E*	OL B Cusp	IL Basis				<.0001*
*H*	OL C Stylus, term	IL Bulge				<.0001*	*E*	OL B Cusp	IL Bulge				<.0001*
*H*	OL C Cusp	IL Basis				<.0001*	*E*	OL C Stylus, term	IL Basis				<.0001*
*H*	OL C Cusp	IL Bulge				<.0001*	*E*	OL C Stylus, term	IL Bulge				<.0001*
*H*	OL D Stylus, term	IL Basis				<.0001*	*E*	OL C Cusp	IL Basis				<.0001*
*H*	OL D Stylus, term	IL Bulge				<.0001*	*E*	OL C Cusp	IL Bulge				<.0001*
*H*	OL Cusp	IL Basis				<.0001*	*E*	OL D Stylus, term	IL Basis				<.0001*
*H*	OL Cusp	IL Bulge				<.0001*	*E*	OL D Stylus, term	IL Bulge				<.0001*
*H*	OL E Stylus, term	IL Basis				<.0001*	*E*	OL D Cusp	IL Basis				<.0001*
*H*	OL E Stylus, term	IL Bulge				<.0001*	*E*	OL D Cusp	IL Bulge				<.0001*
*H*	OL E Cusp	IL Basis				<.0001*	*E*	OL E Stylus, term	IL Basis				<.0001*
*H*	OL E Cusp	IL Bulge				<.0001*	*E*	OL E Stylus, term	IL Bulge				<.0001*
*H*	OL A Cusp	IL Stylus, basis				<.0001*	*E*	OL E Cusp	IL Basis				<.0001*
*H*	OL A Basis	IL Bulge				<.0001*	*E*	OL E Cusp	IL Bulge				<.0001*
*H*	OL A Stylus, basis	IL Basis				<.0001*	*E*	OL A Basis	IL Bulge				<.0001*
*H*	OL A Stylus, basis	IL Bulge				<.0001*	*E*	OL A Stylus, basis	IL Basis				<.0001*
*H*	OL B Basis	IL Bulge				<.0001*	*E*	OL A Stylus, basis	IL Bulge				<.0001*
*H*	OL B Stylus, basis	IL Basis				<.0001*	*E*	OL B Basis	IL Bulge				<.0001*
*H*	OL B Stylus, basis	IL Bulge				<.0001*	*E*	OL B Stylus, basis	IL Basis				<.0001*
*H*	OL C Stylus, basis	IL Basis				<.0001*	*E*	OL B Stylus, basis	IL Bulge				<.0001*
*H*	OL C Stylus, basis	IL Bulge				<.0001*	*E*	OL C Stylus, basis	IL Basis				<.0001*
*H*	OL D Stylus, basis	IL Basis				<.0001*	*E*	OL C Stylus, basis	IL Bulge				<.0001*
*H*	OL D Stylus, basis	IL Bulge				<.0001*	*E*	OL D Stylus, basis	IL Basis				<.0001*
*H*	OL E Stylus, basis	IL Basis				<.0001*	*E*	OL D Stylus, basis	IL Bulge				<.0001*
*H*	OL E Stylus, basis	IL Bulge				<.0001*	*E*	OL E Stylus, basis	IL Basis				<.0001*
*H*	IL Cusp	IL Stylus, term				<.0001*	*E*	OL E Stylus, basis	IL Bulge				<.0001*
*H*	OL C Basis	IL Bulge				<.0001*	*E*	OL C Basis	IL Bulge				<.0001*
*H*	OL D Basis	IL Bulge				<.0001*	*E*	OL D Basis	IL Bulge				<.0001*
*H*	OL B Cusp	IL Stylus, basis				.0001*	*E*	OL B Cusp	IL Stylus, basis				<.0001*
*H*	OL E Basis	IL Bulge				.0002*	*E*	OL E Basis	IL Bulge				.0002*
*H*	OL A Stylus, term	OL A Basis				<.0001*	*E*	OL A Cusp	OL A Stylus, term				<.0001*
*H*	OL A Stylus, term	OL A Bulge				<.0001*	*E*	OL A Stylus, term	OL A Basis				<.0001*
*H*	OL A Cusp	OL A Basis				<.0001*	*E*	OL A Stylus, term	OL A Bulge				<.0001*
*H*	OL A Cusp	OL A Bulge				<.0001*	*E*	OL A Cusp	OL A Basis				<.0001*
*H*	OL B Stylus, term	OL A Basis				<.0001*	*E*	OL A Cusp	OL A Bulge				<.0001*
*H*	OL B Stylus, term	OL A Bulge				<.0001*	*E*	OL A Cusp	OL A Stylus, basis				<.0001*
*H*	OL B Stylus, term	OL B Basis				<.0001*	*E*	OL B Stylus, term	OL A Basis				<.0001*
*H*	OL B Stylus, term	OL B Bulge				<.0001*	*E*	OL B Stylus, term	OL A Bulge				<.0001*
*H*	OL B Cusp	OL A Basis				<.0001*	*E*	OL B Stylus, term	OL B Basis				<.0001*
*H*	OL B Cusp	OL A Bulge				<.0001*	*E*	OL B Stylus, term	OL B Bulge				<.0001*
*H*	OL B Cusp	OL B Basis				<.0001*	*E*	OL B Cusp	OL A Basis				<.0001*
*H*	OL B Cusp	OL B Bulge				<.0001*	*E*	OL B Cusp	OL A Bulge				<.0001*
*H*	OL C Stylus, term	OL A Basis				<.0001*	*E*	OL B Cusp	OL B Basis				<.0001*
*H*	OL C Stylus, term	OL A Bulge				<.0001*	*E*	OL B Cusp	OL B Bulge				<.0001*
*H*	OL C Stylus, term	OL B Basis				<.0001*	*E*	OL B Cusp	OL B Stylus, basis				<.0001*
*H*	OL C Stylus, term	OL B Bulge				<.0001*	*E*	OL C Stylus, term	OL A Basis				<.0001*
*H*	OL C Stylus, term	OL C Basis				<.0001*	*E*	OL C Stylus, term	OL A Bulge				<.0001*
*H*	OL C Stylus, term	OL C Bulge				<.0001*	*E*	OL C Stylus, term	OL B Basis				<.0001*
*H*	OL C Cusp	OL A Basis				<.0001*	*E*	OL C Stylus, term	OL B Bulge				<.0001*
*H*	OL C Cusp	OL A Bulge				<.0001*	*E*	OL C Stylus, term	OL C Basis				<.0001*
*H*	OL C Cusp	OL B Basis				<.0001*	*E*	OL C Stylus, term	OL C Bulge				<.0001*
*H*	OL C Cusp	OL B Bulge				<.0001*	*E*	OL C Cusp	OL A Basis				<.0001*
*H*	OL C Cusp	OL C Basis				<.0001*	*E*	OL C Cusp	OL A Bulge				<.0001*
*H*	OL C Cusp	OL C Bulge				<.0001*	*E*	OL C Cusp	OL B Basis				<.0001*
*H*	OL D Stylus, term	OL A Basis				<.0001*	*E*	OL C Cusp	OL B Bulge				<.0001*
*H*	OL D Stylus, term	OL A Bulge				<.0001*	*E*	OL C Cusp	OL C Basis				<.0001*
*H*	OL D Stylus, term	OL B Basis				<.0001*	*E*	OL C Cusp	OL C Bulge				<.0001*
*H*	OL D Stylus, term	OL B Bulge				<.0001*	*E*	OL C Cusp	OL C Stylus, basis				<.0001*
*H*	OL D Stylus, term	OL C Basis				<.0001*	*E*	OL D Stylus, term	OL A Basis				<.0001*
*H*	OL D Stylus, term	OL C Bulge				<.0001*	*E*	OL D Stylus, term	OL A Bulge				<.0001*
*H*	OL D Stylus, term	OL D Basis				<.0001*	*E*	OL D Stylus, term	OL B Basis				<.0001*
*H*	OL D Stylus, term	OL D Bulge				<.0001*	*E*	OL D Stylus, term	OL B Bulge				<.0001*
*H*	OL Cusp	OL A Basis				<.0001*	*E*	OL D Stylus, term	OL C Basis				<.0001*
*H*	OL Cusp	OL A Bulge				<.0001*	*E*	OL D Stylus, term	OL C Bulge				<.0001*
*H*	OL Cusp	OL B Basis				<.0001*	*E*	OL D Stylus, term	OL D Basis				<.0001*
*H*	OL Cusp	OL B Bulge				<.0001*	*E*	OL D Stylus, term	OL D Bulge				<.0001*
*H*	OL Cusp	OL C Basis				<.0001*	*E*	OL D Cusp	OL A Basis				<.0001*
*H*	OL Cusp	OL C Bulge				<.0001*	*E*	OL D Cusp	OL A Bulge				<.0001*
*H*	OL Cusp	OL D Basis				<.0001*	*E*	OL D Cusp	OL B Basis				<.0001*
*H*	OL Cusp	OL D Bulge				<.0001*	*E*	OL D Cusp	OL B Bulge				<.0001*
*H*	OL E Stylus, term	OL A Basis				<.0001*	*E*	OL D Cusp	OL C Basis				<.0001*
*H*	OL E Stylus, term	OL A Bulge				<.0001*	*E*	OL D Cusp	OL C Bulge				<.0001*
*H*	OL E Stylus, term	OL B Basis				<.0001*	*E*	OL D Cusp	OL D Basis				<.0001*
*H*	OL E Stylus, term	OL B Bulge				<.0001*	*E*	OL D Cusp	OL D Bulge				<.0001*
*H*	OL E Stylus, term	OL C Basis				<.0001*	*E*	OL D Cusp	OL D Stylus, basis				<.0001*
*H*	OL E Stylus, term	OL C Bulge				<.0001*	*E*	OL E Stylus, term	OL A Basis				<.0001*
*H*	OL E Stylus, term	OL D Basis				<.0001*	*E*	OL E Stylus, term	OL A Bulge				<.0001*
*H*	OL E Stylus, term	OL D Bulge				<.0001*	*E*	OL E Stylus, term	OL B Basis				<.0001*
*H*	OL E Stylus, term	OL E Basis				<.0001*	*E*	OL E Stylus, term	OL B Bulge				<.0001*
*H*	OL E Stylus, term	OL E Bulge				<.0001*	*E*	OL E Stylus, term	OL C Basis				<.0001*
*H*	OL E Cusp	OL A Basis				<.0001*	*E*	OL E Stylus, term	OL C Bulge				<.0001*
*H*	OL E Cusp	OL A Bulge				<.0001*	*E*	OL E Stylus, term	OL D Basis				<.0001*
*H*	OL E Cusp	OL B Basis				<.0001*	*E*	OL E Stylus, term	OL D Bulge				<.0001*
*H*	OL E Cusp	OL B Bulge				<.0001*	*E*	OL E Stylus, term	OL E Basis				<.0001*
*H*	OL E Cusp	OL C Basis				<.0001*	*E*	OL E Stylus, term	OL E Bulge				<.0001*
*H*	OL E Cusp	OL C Bulge				<.0001*	*E*	OL E Cusp	OL A Basis				<.0001*
*H*	OL E Cusp	OL D Basis				<.0001*	*E*	OL E Cusp	OL A Bulge				<.0001*
*H*	OL E Cusp	OL D Bulge				<.0001*	*E*	OL E Cusp	OL B Basis				<.0001*
*H*	OL E Cusp	OL E Basis				<.0001*	*E*	OL E Cusp	OL B Bulge				<.0001*
*H*	OL E Cusp	OL E Bulge				<.0001*	*E*	OL E Cusp	OL C Basis				<.0001*
*H*	OL A Stylus, term	IL Stylus, basis				.0015*	*E*	OL E Cusp	OL C Bulge				<.0001*
*H*	OL B Cusp	OL B Stylus, basis				<.0001*	*E*	OL E Cusp	OL D Basis				<.0001*
*H*	OL C Cusp	OL C Stylus, basis				<.0001*	*E*	OL E Cusp	OL D Bulge				<.0001*
*H*	OL A Cusp	OL A Stylus, basis				<.0001*	*E*	OL E Cusp	OL E Basis				<.0001*
*H*	OL E Cusp	OL E Stylus, basis				.0001*	*E*	OL E Cusp	OL E Bulge				<.0001*
*H*	OL Cusp	OL D Stylus, basis				.0001*	*E*	OL E Cusp	OL E Stylus, basis				<.0001*
*H*	OL A Stylus, basis	OL A Basis				<.0001*	*E*	OL B Cusp	OL A Stylus, basis				<.0001*
*H*	OL A Stylus, basis	OL A Bulge				<.0001*	*E*	OL B Cusp	OL B Stylus, term				.0001*
*H*	OL B Basis	OL A Bulge				<.0001*	*E*	OL C Cusp	OL C Stylus, term				.0001*
*H*	OL B Stylus, basis	OL A Basis				<.0001*	*E*	OL E Cusp	OL E Stylus, term				.0001*
*H*	OL B Stylus, basis	OL A Bulge				<.0001*	*E*	OL D Cusp	OL D Stylus, term				.0002*
*H*	OL B Stylus, basis	OL B Basis				<.0001*	*E*	OL A Stylus, basis	OL A Basis				<.0001*
*H*	OL B Stylus, basis	OL B Bulge				<.0001*	*E*	OL A Stylus, basis	OL A Bulge				<.0001*
*H*	OL C Stylus, basis	OL A Basis				<.0001*	*E*	OL B Basis	OL A Bulge				<.0001*
*H*	OL C Stylus, basis	OL A Bulge				<.0001*	*E*	OL B Stylus, basis	OL A Basis				<.0001*
*H*	OL C Stylus, basis	OL B Basis				<.0001*	*E*	OL B Stylus, basis	OL A Bulge				<.0001*
*H*	OL C Stylus, basis	OL B Bulge				<.0001*	*E*	OL B Stylus, basis	OL B Basis				<.0001*
*H*	OL C Stylus, basis	OL C Basis				<.0001*	*E*	OL B Stylus, basis	OL B Bulge				<.0001*
*H*	OL C Stylus, basis	OL C Bulge				<.0001*	*E*	OL C Basis	OL A Bulge				<.0001*
*H*	OL D Stylus, basis	OL A Basis				<.0001*	*E*	OL C Stylus, basis	OL A Basis				<.0001*
*H*	OL D Stylus, basis	OL A Bulge				<.0001*	*E*	OL C Stylus, basis	OL A Bulge				<.0001*
*H*	OL D Stylus, basis	OL B Basis				<.0001*	*E*	OL C Stylus, basis	OL B Basis				<.0001*
*H*	OL D Stylus, basis	OL B Bulge				<.0001*	*E*	OL C Stylus, basis	OL B Bulge				<.0001*
*H*	OL D Stylus, basis	OL C Basis				<.0001*	*E*	OL C Stylus, basis	OL C Basis				<.0001*
*H*	OL D Stylus, basis	OL C Bulge				<.0001*	*E*	OL C Stylus, basis	OL C Bulge				<.0001*
*H*	OL D Stylus, basis	OL D Basis				<.0001*	*E*	OL D Stylus, basis	OL A Basis				<.0001*
*H*	OL D Stylus, basis	OL D Bulge				<.0001*	*E*	OL D Stylus, basis	OL A Bulge				<.0001*
*H*	OL E Stylus, basis	OL A Basis				<.0001*	*E*	OL D Stylus, basis	OL B Basis				<.0001*
*H*	OL E Stylus, basis	OL A Bulge				<.0001*	*E*	OL D Stylus, basis	OL B Bulge				<.0001*
*H*	OL E Stylus, basis	OL B Basis				<.0001*	*E*	OL D Stylus, basis	OL C Basis				<.0001*
*H*	OL E Stylus, basis	OL B Bulge				<.0001*	*E*	OL D Stylus, basis	OL C Bulge				<.0001*
*H*	OL E Stylus, basis	OL C Basis				<.0001*	*E*	OL D Stylus, basis	OL D Basis				<.0001*
*H*	OL E Stylus, basis	OL C Bulge				<.0001*	*E*	OL D Stylus, basis	OL D Bulge				<.0001*
*H*	OL E Stylus, basis	OL D Basis				<.0001*	*E*	OL E Stylus, basis	OL A Basis				<.0001*
*H*	OL E Stylus, basis	OL D Bulge				<.0001*	*E*	OL E Stylus, basis	OL A Bulge				<.0001*
*H*	OL E Stylus, basis	OL E Basis				<.0001*	*E*	OL E Stylus, basis	OL B Basis				<.0001*
*H*	OL E Stylus, basis	OL E Bulge				<.0001*	*E*	OL E Stylus, basis	OL B Bulge				<.0001*
*H*	OL B Cusp	OL A Stylus, basis				.0002*	*E*	OL E Stylus, basis	OL C Basis				<.0001*
*H*	OL C Basis	OL A Bulge				.0001*	*E*	OL E Stylus, basis	OL C Bulge				<.0001*
*H*	OL D Basis	OL A Bulge				.0002*	*E*	OL E Stylus, basis	OL D Basis				<.0001*
*H*	OL B Stylus, term	OL B Stylus, basis				.0010*	*E*	OL E Stylus, basis	OL D Bulge				<.0001*
*H*	OL C Stylus, term	OL C Stylus, basis				.0010*	*E*	OL E Stylus, basis	OL E Basis				<.0001*
*H*	OL A Stylus, term	OL A Stylus, basis				.0011*	*E*	OL E Stylus, basis	OL E Bulge				<.0001*
*H*	OL E Basis	OL A Bulge				.0008*	*E*	OL D Basis	OL A Bulge				.0001*
*H*	OL A Cusp	OL A Stylus, term				.0025*	*E*	OL A Stylus, term	OL A Stylus, basis				.0003*
*H*	OL B Cusp	OL B Stylus, term				.0025*	*E*	OL C Cusp	OL B Stylus, basis				.0003*
*H*	OL C Cusp	OL C Stylus, term				.0025*	*E*	OL D Cusp	OL C Stylus, basis				.0003*
*H*	OL Cusp	OL C Stylus, basis				.0018*	*E*	OL E Basis	OL A Bulge				.0004*
*H*	OL E Cusp	OL E Stylus, term				.0029*	*E*	OL B Stylus, term	OL B Stylus, basis				.0008*
*H*	OL C Cusp	OL B Stylus, basis				.0021*	*E*	OL B Cusp	OL A Stylus, term				.0012*
*H*	OL Cusp	OL D Stylus, term				.0035*	*E*	OL E Basis	OL D Bulge				.0008*
*H*	OL D Stylus, term	OL D Stylus, basis				.0026*	*E*	OL C Stylus, term	OL C Stylus, basis				.0014*
*H*	OL E Stylus, term	OL E Stylus, basis				.0026*	*E*	OL E Cusp	OL D Stylus, basis				.0018*
*H*	OL E Basis	OL D Bulge				.0020*	*E*	OL A Cusp	IL Stylus, term				.0231*
*H*	OL C Cusp	IL Stylus, basis				.0249*	*E*	OL C Cusp	IL Stylus, basis				.0293*
*H*	OL E Cusp	OL D Stylus, basis				.0045*	*E*	OL C Cusp	OL A Stylus, basis				.0054*
*H*	OL C Basis	OL B Bulge				.0031*	*E*	OL A Stylus, term	IL Stylus, basis				.0344*
*H*	OL A Cusp	IL Stylus, term				.0322*	*E*	OL C Basis	OL B Bulge				.0047*
*H*	OL D Basis	OL C Bulge				.0071*	*E*	OL D Stylus, term	OL D Stylus, basis				.0077*
*H*	OL C Cusp	OL A Stylus, basis				.0175*	*E*	OL E Stylus, term	OL E Stylus, basis				.0077*
*H*	OL B Stylus, term	OL A Stylus, basis				.0205*	*E*	OL D Basis	OL C Bulge				.0058*
*H*	OL B Cusp	OL A Stylus, term				.0274*	*E*	OL E Basis	OL C Bulge				.0215*
*H*	OL D Basis	OL B Bulge				.0256*	*E*	OL D Basis	OL B Bulge				.0256*
*H*	OL E Basis	OL C Bulge				.0302*	*E*	OL D Cusp	OL C Stylus, term				.0578
*H*	OL Cusp	OL B Stylus, basis				.0426*	*E*	OL B Stylus, term	OL A Stylus, basis				.0559
*H*	OL E Cusp	OL C Stylus, basis				.0822	*E*	OL C Cusp	OL B Stylus, term				.0727
*H*	OL D Bulge	OL A Bulge				.0877	*E*	OL D Cusp	OL B Stylus, basis				.0822
*H*	OL B Cusp	IL Stylus, term				.1989	*E*	OL B Bulge	OL A Bulge				.1150
*H*	OL Cusp	OL C Stylus, term				.1239	*E*	OL D Bulge	OL A Bulge				.1310
*H*	OL B Bulge	OL A Bulge				.1006	*E*	OL E Basis	OL B Bulge				.1310
*H*	OL E Basis	OL B Bulge				.1310	*E*	OL B Bulge	IL Bulge				.2945
*H*	OL C Cusp	OL B Stylus, term				.1662	*E*	OL E Cusp	OL C Stylus, basis				.2024
*H*	OL B Bulge	IL Bulge				.2785	*E*	OL C Bulge	OL A Bulge				.2122
*H*	OL Cusp	OL A Stylus, basis				.1643	*E*	OL B Cusp	IL Stylus, term				.3564
*H*	OL C Bulge	OL A Bulge				.1484	*E*	OL E Cusp	OL D Stylus, term				.2815
*H*	OL E Cusp	OL D Stylus, term				.1824	*E*	OL C Bulge	OL B Bulge				.2934
*H*	OL B Stylus, term	IL Stylus, basis				.3163	*E*	OL E Bulge	OL A Bulge				.2934
*H*	OL A Cusp	IL Cusp				.3073	*E*	OL D Bulge	OL C Bulge				.3933
*H*	OL Cusp	IL Stylus, basis				.3816	*E*	OL D Cusp	OL A Stylus, basis				.4869
*H*	OL E Bulge	OL A Bulge				.3245	*E*	OL D Bulge	IL Bulge				.6569
*H*	OL D Stylus, term	OL C Stylus, basis				.3539	*E*	OL C Cusp	OL A Stylus, term				.6444
*H*	OL C Cusp	OL A Stylus, term				.3833	*E*	OL D Stylus, term	OL C Stylus, basis				.6851
*H*	OL C Stylus, term	OL B Stylus, basis				.4173	*E*	OL D Bulge	OL B Bulge				.7928
*H*	OL C Bulge	OL B Bulge				.5114	*E*	OL C Bulge	IL Bulge				.9292
*H*	OL D Bulge	IL Bulge				.6828	*E*	OL C Stylus, term	OL B Stylus, basis				1.0000
*H*	OL D Bulge	OL C Bulge				.6936	*E*	OL B Stylus, term	IL Stylus, basis				.9873
*H*	OL C Bulge	IL Bulge				.7899	*E*	OL E Bulge	IL Bulge				.9858
*H*	OL Cusp	OL B Stylus, term				.7976	*E*	OL E Bulge	OL D Bulge				.4307
*H*	OL E Stylus, term	OL D Stylus, basis				.8167	*E*	OL D Cusp	OL B Stylus, term				.4418
*H*	OL E Cusp	OL B Stylus, basis				.8620	*E*	OL A Cusp	IL Cusp				.4892
*H*	OL D Bulge	OL B Bulge				.8955	*E*	OL D Cusp	IL Stylus, basis				.4741
*H*	OL B Cusp	IL Cusp				.9475	*E*	OL B Basis	OL A Basis				.3246
*H*	OL E Cusp	OL C Stylus, term				.9183	*E*	OL E Stylus, term	OL D Stylus, basis				.3247
*H*	OL C Cusp	IL Stylus, term				.9213	*E*	OL D Basis	OL C Basis				.2372
*H*	OL E Bulge	IL Bulge				.8451	*E*	OL E Cusp	OL B Stylus, basis				.2466
*H*	OL Cusp	OL A Stylus, term				.5727	*E*	OL C Basis	OL B Basis				.1891
*H*	OL E Cusp	OL A Stylus, basis				.4868	*E*	OL E Bulge	OL C Bulge				.1891
*H*	OL C Stylus, term	OL A Stylus, basis				.4512	*E*	OL B Cusp	OL A Cusp				.1584
*H*	OL B Basis	OL A Basis				.4306	*E*	OL E Basis	OL D Basis				.1150
*H*	OL E Bulge	OL D Bulge				.3933	*E*	OL E Cusp	OL C Stylus, term				.1370
*H*	OL E Bulge	OL C Bulge				.3246	*E*	OL A Basis	IL Basis				.2202
*H*	OL C Basis	OL B Basis				.2643	*E*	OL C Stylus, term	OL A Stylus, basis				.0929
*H*	OL D Basis	OL C Basis				.2643	*E*	OL E Bulge	OL B Bulge				.0762
*H*	OL B Cusp	OL A Cusp				.2930	*E*	OL B Stylus, basis	OL A Stylus, basis				.0660
*H*	OL A Basis	IL Basis				.3285	*E*	OL C Basis	OL A Basis				.0569
*H*	OL B Stylus, basis	OL A Stylus, basis				.1678	*E*	OL A Bulge	IL Bulge				.1059
*H*	OL E Basis	OL D Basis				.1486	*E*	OL B Basis	IL Basis				.0985
*H*	OL E Bulge	OL B Bulge				.1310	*E*	OL D Basis	OL B Basis				.0256*
*H*	OL D Stylus, term	OL B Stylus, basis				.0725	*E*	OL D Cusp	OL A Stylus, term				.0355*
*H*	OL C Basis	OL A Basis				.0488*	*E*	OL B Stylus, term	OL A Stylus, term				.0257*
*H*	OL D Stylus, basis	OL C Stylus, basis				.0488*	*E*	OL C Cusp	OL B Cusp				.0240*
*H*	OL Cusp	OL C Cusp				.0649	*E*	OL E Basis	OL C Basis				.0126*
*H*	OL B Stylus, term	OL A Stylus, term				.0612	*E*	OL D Cusp	OL C Cusp				.0183*
*H*	OL C Cusp	OL B Cusp				.0578	*E*	OL D Stylus, basis	OL C Stylus, basis				.0104*
*H*	OL A Stylus, basis	IL Stylus, basis				.1221	*E*	OL D Basis	OL A Basis				.0086*
*H*	OL E Cusp	OL Cusp				.0513	*E*	OL E Cusp	OL A Stylus, basis				.0108*
*H*	OL C Stylus, basis	OL B Stylus, basis				.0256*	*E*	OL C Stylus, basis	OL B Stylus, basis				.0058*
*H*	OL A Bulge	IL Bulge				.0914	*E*	OL C Cusp	IL Stylus, term				.0323*
*H*	OL B Basis	IL Basis				.0914	*E*	OL E Cusp	OL D Cusp				.0089*
*H*	OL E Stylus, basis	OL D Stylus, basis				.0215*	*E*	OL A Stylus, basis	IL Stylus, basis				.0345*
*H*	OL E Stylus, term	OL C Stylus, basis				.0277*	*E*	OL E Stylus, basis	OL D Stylus, basis				.0039*
*H*	OL D Basis	OL B Basis				.0181*	*E*	OL D Stylus, term	OL B Stylus, basis				.0054*
*H*	OL E Basis	OL C Basis				.0151*	*E*	OL B Cusp	IL Cusp				.0231*
*H*	OL E Cusp	OL B Stylus, term				.0240*	*E*	OL C Bulge	OL C Basis				.0025*
*H*	OL C Cusp	OL A Cusp				.0210*	*E*	OL E Basis	OL B Basis				.0016*
*H*	OL D Basis	OL A Basis				.0104*	*E*	OL E Bulge	OL E Basis				.0016*
*H*	OL E Cusp	IL Stylus, basis				.0504	*E*	OL E Stylus, term	OL C Stylus, basis				.0018*
*H*	OL Cusp	OL B Cusp				.0077*	*E*	OL C Cusp	OL A Cusp				.0021*
*H*	OL E Cusp	OL C Cusp				.0056*	*E*	OL C Stylus, basis	OL A Stylus, basis				.0004*
*H*	OL C Stylus, basis	OL A Stylus, basis				.0025*	*E*	OL E Basis	OL A Basis				.0004*
*H*	OL D Stylus, term	OL A Stylus, basis				.0038*	*E*	OL D Stylus, term	OL A Stylus, basis				.0006*
*H*	OL E Bulge	OL E Basis				.0020*	*E*	OL B Bulge	OL B Basis				.0003*
*H*	OL C Bulge	OL C Basis				.0016*	*E*	OL C Bulge	OL B Basis				.0003*
*H*	OL E Basis	OL B Basis				.0013*	*E*	OL D Stylus, term	OL C Stylus, term				.0006*
*H*	OL D Stylus, term	OL C Stylus, term				.0021*	*E*	OL C Bulge	OL A Basis				.0002*
*H*	OL Cusp	OL A Cusp				.0018*	*E*	OL E Bulge	OL D Basis				.0002*
*H*	OL C Cusp	IL Cusp				.0093*	*E*	OL B Bulge	OL A Basis				.0001*
*H*	OL D Stylus, basis	OL B Stylus, basis				.0005*	*E*	OL E Stylus, term	OL D Stylus, term				.0004*
*H*	OL E Basis	OL A Basis				.0004*	*E*	OL D Bulge	OL D Basis				.0001*
*H*	OL C Stylus, term	OL B Stylus, term				.0010*	*E*	OL D Cusp	OL B Cusp				.0003*
*H*	OL C Stylus, term	IL Stylus, basis				.0079*	*E*	OL A Bulge	OL A Basis				<.0001*
*H*	OL B Bulge	OL B Basis				.0003*	*E*	OL B Basis	OL A Stylus, basis				<.0001*
*H*	OL C Bulge	OL B Basis				.0003*	*E*	OL B Bulge	OL A Stylus, basis				<.0001*
*H*	OL E Stylus, basis	OL C Stylus, basis				.0003*	*E*	OL C Basis	OL A Stylus, basis				<.0001*
*H*	OL E Stylus, term	OL D Stylus, term				.0009*	*E*	OL C Basis	OL B Stylus, basis				<.0001*
*H*	OL B Bulge	OL A Basis				.0002*	*E*	OL C Bulge	OL A Stylus, basis				<.0001*
*H*	OL C Bulge	OL A Basis				.0002*	*E*	OL C Bulge	OL B Stylus, basis				<.0001*
*H*	OL D Stylus, basis	OL A Stylus, basis				.0002*	*E*	OL D Basis	OL A Stylus, basis				<.0001*
*H*	OL E Bulge	OL D Basis				.0002*	*E*	OL D Basis	OL B Stylus, basis				<.0001*
*H*	OL D Bulge	OL D Basis				.0001*	*E*	OL D Basis	OL C Stylus, basis				<.0001*
*H*	OL E Cusp	OL A Stylus, term				.0004*	*E*	OL D Bulge	OL A Basis				<.0001*
*H*	OL B Stylus, basis	IL Stylus, basis				.0042*	*E*	OL D Bulge	OL A Stylus, basis				<.0001*
*H*	OL A Bulge	OL A Basis				<.0001*	*E*	OL D Bulge	OL B Basis				<.0001*
*H*	OL B Basis	OL A Stylus, basis				<.0001*	*E*	OL D Bulge	OL B Stylus, basis				<.0001*
*H*	OL B Bulge	OL A Stylus, basis				<.0001*	*E*	OL D Bulge	OL C Basis				<.0001*
*H*	OL C Basis	OL A Stylus, basis				<.0001*	*E*	OL D Bulge	OL C Stylus, basis				<.0001*
*H*	OL C Basis	OL B Stylus, basis				<.0001*	*E*	OL D Stylus, basis	OL A Stylus, basis				<.0001*
*H*	OL C Bulge	OL A Stylus, basis				<.0001*	*E*	OL D Stylus, basis	OL B Stylus, basis				<.0001*
*H*	OL C Bulge	OL B Stylus, basis				<.0001*	*E*	OL E Basis	OL A Stylus, basis				<.0001*
*H*	OL D Basis	OL A Stylus, basis				<.0001*	*E*	OL E Basis	OL B Stylus, basis				<.0001*
*H*	OL D Basis	OL B Stylus, basis				<.0001*	*E*	OL E Basis	OL C Stylus, basis				<.0001*
*H*	OL D Basis	OL C Stylus, basis				<.0001*	*E*	OL E Basis	OL D Stylus, basis				<.0001*
*H*	OL D Bulge	OL A Basis				<.0001*	*E*	OL E Bulge	OL A Basis				<.0001*
*H*	OL D Bulge	OL A Stylus, basis				<.0001*	*E*	OL E Bulge	OL A Stylus, basis				<.0001*
*H*	OL D Bulge	OL B Basis				<.0001*	*E*	OL E Bulge	OL B Basis				<.0001*
*H*	OL D Bulge	OL B Stylus, basis				<.0001*	*E*	OL E Bulge	OL B Stylus, basis				<.0001*
*H*	OL D Bulge	OL C Basis				<.0001*	*E*	OL E Bulge	OL C Basis				<.0001*
*H*	OL D Bulge	OL C Stylus, basis				<.0001*	*E*	OL E Bulge	OL C Stylus, basis				<.0001*
*H*	OL E Basis	OL A Stylus, basis				<.0001*	*E*	OL E Bulge	OL D Stylus, basis				<.0001*
*H*	OL E Basis	OL B Stylus, basis				<.0001*	*E*	OL E Stylus, basis	OL A Stylus, basis				<.0001*
*H*	OL E Basis	OL C Stylus, basis				<.0001*	*E*	OL E Stylus, basis	OL B Stylus, basis				<.0001*
*H*	OL E Basis	OL D Stylus, basis				<.0001*	*E*	OL E Stylus, basis	OL C Stylus, basis				<.0001*
*H*	OL E Bulge	OL A Basis				<.0001*	*E*	OL C Stylus, term	OL B Stylus, term				.0002*
*H*	OL E Bulge	OL A Stylus, basis				<.0001*	*E*	OL E Cusp	OL B Stylus, term				.0002*
*H*	OL E Bulge	OL B Basis				<.0001*	*E*	OL C Basis	IL Basis				.0024*
*H*	OL E Bulge	OL B Stylus, basis				<.0001*	*E*	OL E Cusp	OL C Cusp				<.0001*
*H*	OL E Bulge	OL C Basis				<.0001*	*E*	OL B Stylus, basis	IL Stylus, basis				.0017*
*H*	OL E Bulge	OL C Stylus, basis				<.0001*	*E*	OL B Basis	OL A Stylus, term				<.0001*
*H*	OL E Bulge	OL D Stylus, basis				<.0001*	*E*	OL B Basis	OL A Cusp				<.0001*
*H*	OL E Stylus, basis	OL A Stylus, basis				<.0001*	*E*	OL B Bulge	OL A Stylus, term				<.0001*
*H*	OL E Stylus, basis	OL B Stylus, basis				<.0001*	*E*	OL B Bulge	OL A Cusp				<.0001*
*H*	OL B Stylus, basis	OL A Stylus, term				.0001*	*E*	OL B Stylus, basis	OL A Stylus, term				<.0001*
*H*	OL Cusp	IL Stylus, term				.0024*	*E*	OL B Stylus, basis	OL A Cusp				<.0001*
*H*	OL E Stylus, term	OL B Stylus, basis				.0001*	*E*	OL C Basis	OL A Stylus, term				<.0001*
*H*	OL C Basis	IL Basis				.0027*	*E*	OL C Basis	OL A Cusp				<.0001*
*H*	OL B Stylus, term	OL A Cusp				.0001*	*E*	OL C Basis	OL B Stylus, term				<.0001*
*H*	OL E Cusp	OL B Cusp				.0001*	*E*	OL C Basis	OL B Cusp				<.0001*
*H*	OL D Stylus, basis	OL C Stylus, term				<.0001*	*E*	OL C Bulge	OL A Stylus, term				<.0001*
*H*	OL E Stylus, basis	OL D Stylus, term				<.0001*	*E*	OL C Bulge	OL A Cusp				<.0001*
*H*	OL C Stylus, basis	OL B Stylus, term				<.0001*	*E*	OL C Bulge	OL B Stylus, term				<.0001*
*H*	OL B Basis	OL A Stylus, term				<.0001*	*E*	OL C Bulge	OL B Cusp				<.0001*
*H*	OL B Basis	OL A Cusp				<.0001*	*E*	OL C Stylus, basis	OL A Stylus, term				<.0001*
*H*	OL B Bulge	OL A Stylus, term				<.0001*	*E*	OL C Stylus, basis	OL A Cusp				<.0001*
*H*	OL B Bulge	OL A Cusp				<.0001*	*E*	OL C Stylus, basis	OL B Stylus, term				<.0001*
*H*	OL B Stylus, basis	OL A Cusp				<.0001*	*E*	OL C Stylus, basis	OL B Cusp				<.0001*
*H*	OL C Basis	OL A Stylus, term				<.0001*	*E*	OL D Basis	OL A Stylus, term				<.0001*
*H*	OL C Basis	OL A Cusp				<.0001*	*E*	OL D Basis	OL A Cusp				<.0001*
*H*	OL C Basis	OL B Stylus, term				<.0001*	*E*	OL D Basis	OL B Stylus, term				<.0001*
*H*	OL C Basis	OL B Cusp				<.0001*	*E*	OL D Basis	OL B Cusp				<.0001*
*H*	OL C Bulge	OL A Stylus, term				<.0001*	*E*	OL D Basis	OL C Stylus, term				<.0001*
*H*	OL C Bulge	OL A Cusp				<.0001*	*E*	OL D Basis	OL C Cusp				<.0001*
*H*	OL C Bulge	OL B Stylus, term				<.0001*	*E*	OL D Bulge	OL A Stylus, term				<.0001*
*H*	OL C Bulge	OL B Cusp				<.0001*	*E*	OL D Bulge	OL A Cusp				<.0001*
*H*	OL C Stylus, basis	OL A Stylus, term				<.0001*	*E*	OL D Bulge	OL B Stylus, term				<.0001*
*H*	OL C Stylus, basis	OL A Cusp				<.0001*	*E*	OL D Bulge	OL B Cusp				<.0001*
*H*	OL C Stylus, basis	OL B Cusp				<.0001*	*E*	OL D Bulge	OL C Stylus, term				<.0001*
*H*	OL D Basis	OL A Stylus, term				<.0001*	*E*	OL D Bulge	OL C Cusp				<.0001*
*H*	OL D Basis	OL A Cusp				<.0001*	*E*	OL D Stylus, basis	OL A Stylus, term				<.0001*
*H*	OL D Basis	OL B Stylus, term				<.0001*	*E*	OL D Stylus, basis	OL A Cusp				<.0001*
*H*	OL D Basis	OL B Cusp				<.0001*	*E*	OL D Stylus, basis	OL B Stylus, term				<.0001*
*H*	OL D Basis	OL C Stylus, term				<.0001*	*E*	OL D Stylus, basis	OL B Cusp				<.0001*
*H*	OL D Basis	OL C Cusp				<.0001*	*E*	OL D Stylus, basis	OL C Stylus, term				<.0001*
*H*	OL D Bulge	OL A Stylus, term				<.0001*	*E*	OL D Stylus, basis	OL C Cusp				<.0001*
*H*	OL D Bulge	OL A Cusp				<.0001*	*E*	OL E Basis	OL A Stylus, term				<.0001*
*H*	OL D Bulge	OL B Stylus, term				<.0001*	*E*	OL E Basis	OL A Cusp				<.0001*
*H*	OL D Bulge	OL B Cusp				<.0001*	*E*	OL E Basis	OL B Stylus, term				<.0001*
*H*	OL D Bulge	OL C Stylus, term				<.0001*	*E*	OL E Basis	OL B Cusp				<.0001*
*H*	OL D Bulge	OL C Cusp				<.0001*	*E*	OL E Basis	OL C Stylus, term				<.0001*
*H*	OL D Stylus, basis	OL A Stylus, term				<.0001*	*E*	OL E Basis	OL C Cusp				<.0001*
*H*	OL D Stylus, basis	OL A Cusp				<.0001*	*E*	OL E Basis	OL D Stylus, term				<.0001*
*H*	OL D Stylus, basis	OL B Stylus, term				<.0001*	*E*	OL E Basis	OL D Cusp				<.0001*
*H*	OL D Stylus, basis	OL B Cusp				<.0001*	*E*	OL E Bulge	OL A Stylus, term				<.0001*
*H*	OL D Stylus, basis	OL C Cusp				<.0001*	*E*	OL E Bulge	OL A Cusp				<.0001*
*H*	OL E Basis	OL A Stylus, term				<.0001*	*E*	OL E Bulge	OL B Stylus, term				<.0001*
*H*	OL E Basis	OL A Cusp				<.0001*	*E*	OL E Bulge	OL B Cusp				<.0001*
*H*	OL E Basis	OL B Stylus, term				<.0001*	*E*	OL E Bulge	OL C Stylus, term				<.0001*
*H*	OL E Basis	OL B Cusp				<.0001*	*E*	OL E Bulge	OL C Cusp				<.0001*
*H*	OL E Basis	OL C Stylus, term				<.0001*	*E*	OL E Bulge	OL D Stylus, term				<.0001*
*H*	OL E Basis	OL C Cusp				<.0001*	*E*	OL E Bulge	OL D Cusp				<.0001*
*H*	OL E Basis	OL D Stylus, term				<.0001*	*E*	OL E Stylus, basis	OL A Stylus, term				<.0001*
*H*	OL E Basis	OL Cusp				<.0001*	*E*	OL E Stylus, basis	OL A Cusp				<.0001*
*H*	OL E Bulge	OL A Stylus, term				<.0001*	*E*	OL E Stylus, basis	OL B Stylus, term				<.0001*
*H*	OL E Bulge	OL A Cusp				<.0001*	*E*	OL E Stylus, basis	OL B Cusp				<.0001*
*H*	OL E Bulge	OL B Stylus, term				<.0001*	*E*	OL E Stylus, basis	OL C Stylus, term				<.0001*
*H*	OL E Bulge	OL B Cusp				<.0001*	*E*	OL E Stylus, basis	OL C Cusp				<.0001*
*H*	OL E Bulge	OL C Stylus, term				<.0001*	*E*	OL E Stylus, basis	OL D Stylus, term				<.0001*
*H*	OL E Bulge	OL C Cusp				<.0001*	*E*	OL E Stylus, basis	OL D Cusp				<.0001*
*H*	OL E Bulge	OL D Stylus, term				<.0001*	*E*	OL E Stylus, term	OL A Stylus, basis				<.0001*
*H*	OL E Bulge	OL Cusp				<.0001*	*E*	OL E Stylus, term	OL B Stylus, basis				<.0001*
*H*	OL E Stylus, basis	OL A Stylus, term				<.0001*	*E*	OL D Cusp	OL A Cusp				<.0001*
*H*	OL E Stylus, basis	OL A Cusp				<.0001*	*E*	OL B Stylus, term	OL A Cusp				<.0001*
*H*	OL E Stylus, basis	OL B Stylus, term				<.0001*	*E*	OL C Stylus, term	OL A Stylus, term				<.0001*
*H*	OL E Stylus, basis	OL B Cusp				<.0001*	*E*	OL C Stylus, term	OL A Cusp				<.0001*
*H*	OL E Stylus, basis	OL C Stylus, term				<.0001*	*E*	OL C Stylus, term	OL B Cusp				<.0001*
*H*	OL E Stylus, basis	OL C Cusp				<.0001*	*E*	OL D Stylus, term	OL A Stylus, term				<.0001*
*H*	OL E Stylus, basis	OL Cusp				<.0001*	*E*	OL D Stylus, term	OL A Cusp				<.0001*
*H*	OL E Stylus, term	OL A Stylus, basis				<.0001*	*E*	OL D Stylus, term	OL B Stylus, term				<.0001*
*H*	OL C Stylus, term	OL A Stylus, term				<.0001*	*E*	OL D Stylus, term	OL B Cusp				<.0001*
*H*	OL C Stylus, term	OL B Cusp				<.0001*	*E*	OL D Stylus, term	OL C Cusp				<.0001*
*H*	OL D Stylus, term	OL C Cusp				<.0001*	*E*	OL E Stylus, term	OL A Stylus, term				<.0001*
*H*	OL E Cusp	OL A Cusp				<.0001*	*E*	OL E Stylus, term	OL A Cusp				<.0001*
*H*	OL A Stylus, term	IL Stylus, term				.0007*	*E*	OL E Stylus, term	OL B Stylus, term				<.0001*
*H*	OL E Stylus, term	OL Cusp				<.0001*	*E*	OL E Stylus, term	OL B Cusp				<.0001*
*H*	OL D Stylus, term	OL B Stylus, term				<.0001*	*E*	OL E Stylus, term	OL C Stylus, term				<.0001*
*H*	OL C Stylus, term	OL A Cusp				<.0001*	*E*	OL E Stylus, term	OL C Cusp				<.0001*
*H*	OL D Stylus, term	OL A Stylus, term				<.0001*	*E*	OL E Stylus, term	OL D Cusp				<.0001*
*H*	OL D Stylus, term	OL A Cusp				<.0001*	*E*	OL E Cusp	OL A Stylus, term				<.0001*
*H*	OL D Stylus, term	OL B Cusp				<.0001*	*E*	OL E Cusp	OL A Cusp				<.0001*
*H*	OL E Stylus, term	OL A Stylus, term				<.0001*	*E*	OL E Cusp	OL B Cusp				<.0001*
*H*	OL E Stylus, term	OL A Cusp				<.0001*	*E*	OL C Stylus, term	IL Stylus, basis				.0005*
*H*	OL E Stylus, term	OL B Stylus, term				<.0001*	*E*	OL D Basis	IL Basis				.0002*
*H*	OL E Stylus, term	OL B Cusp				<.0001*	*E*	OL E Cusp	IL Stylus, basis				.0001*
*H*	OL E Stylus, term	OL C Stylus, term				<.0001*	*E*	OL E Basis	IL Basis				<.0001*
*H*	OL E Stylus, term	OL C Cusp				<.0001*	*E*	OL A Basis	IL Stylus, term				<.0001*
*H*	OL D Basis	IL Basis				.0002*	*E*	OL A Basis	IL Cusp				<.0001*
*H*	OL B Stylus, term	IL Stylus, term				<.0001*	*E*	OL A Bulge	IL Stylus, term				<.0001*
*H*	OL C Stylus, basis	IL Stylus, basis				<.0001*	*E*	OL A Bulge	IL Cusp				<.0001*
*H*	OL A Stylus, basis	IL Stylus, term				<.0001*	*E*	OL A Stylus, basis	IL Stylus, term				<.0001*
*H*	OL D Stylus, term	IL Stylus, basis				<.0001*	*E*	OL A Stylus, basis	IL Cusp				<.0001*
*H*	OL Cusp	IL Cusp				<.0001*	*E*	OL B Basis	IL Stylus, term				<.0001*
*H*	OL E Basis	IL Basis				<.0001*	*E*	OL B Basis	IL Cusp				<.0001*
*H*	OL A Basis	IL Stylus, term				<.0001*	*E*	OL B Bulge	IL Stylus, term				<.0001*
*H*	OL A Basis	IL Cusp				<.0001*	*E*	OL B Bulge	IL Cusp				<.0001*
*H*	OL A Bulge	IL Stylus, term				<.0001*	*E*	OL B Stylus, basis	IL Stylus, term				<.0001*
*H*	OL A Bulge	IL Cusp				<.0001*	*E*	OL B Stylus, basis	IL Cusp				<.0001*
*H*	OL A Stylus, basis	IL Cusp				<.0001*	*E*	OL C Basis	IL Stylus, term				<.0001*
*H*	OL B Basis	IL Stylus, term				<.0001*	*E*	OL C Basis	IL Cusp				<.0001*
*H*	OL B Basis	IL Cusp				<.0001*	*E*	OL C Bulge	IL Stylus, term				<.0001*
*H*	OL B Bulge	IL Stylus, term				<.0001*	*E*	OL C Bulge	IL Cusp				<.0001*
*H*	OL B Bulge	IL Cusp				<.0001*	*E*	OL C Stylus, basis	IL Stylus, term				<.0001*
*H*	OL B Stylus, basis	IL Stylus, term				<.0001*	*E*	OL C Stylus, basis	IL Cusp				<.0001*
*H*	OL B Stylus, basis	IL Cusp				<.0001*	*E*	OL D Basis	IL Stylus, term				<.0001*
*H*	OL C Basis	IL Stylus, term				<.0001*	*E*	OL D Basis	IL Cusp				<.0001*
*H*	OL C Basis	IL Cusp				<.0001*	*E*	OL D Bulge	IL Stylus, term				<.0001*
*H*	OL C Bulge	IL Stylus, term				<.0001*	*E*	OL D Bulge	IL Cusp				<.0001*
*H*	OL C Bulge	IL Cusp				<.0001*	*E*	OL D Stylus, basis	IL Stylus, term				<.0001*
*H*	OL C Stylus, basis	IL Stylus, term				<.0001*	*E*	OL D Stylus, basis	IL Cusp				<.0001*
*H*	OL C Stylus, basis	IL Cusp				<.0001*	*E*	OL E Basis	IL Stylus, term				<.0001*
*H*	OL D Basis	IL Stylus, term				<.0001*	*E*	OL E Basis	IL Cusp				<.0001*
*H*	OL D Basis	IL Cusp				<.0001*	*E*	OL E Bulge	IL Stylus, term				<.0001*
*H*	OL D Bulge	IL Stylus, term				<.0001*	*E*	OL E Bulge	IL Cusp				<.0001*
*H*	OL D Bulge	IL Cusp				<.0001*	*E*	OL E Stylus, basis	IL Stylus, term				<.0001*
*H*	OL D Stylus, basis	IL Stylus, term				<.0001*	*E*	OL E Stylus, basis	IL Cusp				<.0001*
*H*	OL D Stylus, basis	IL Cusp				<.0001*	*E*	OL A Stylus, term	IL Stylus, term				<.0001*
*H*	OL E Basis	IL Stylus, term				<.0001*	*E*	OL C Cusp	IL Cusp				<.0001*
*H*	OL E Basis	IL Cusp				<.0001*	*E*	OL C Stylus, basis	IL Stylus, basis				<.0001*
*H*	OL E Bulge	IL Stylus, term				<.0001*	*E*	OL A Basis	IL Stylus, basis				<.0001*
*H*	OL E Bulge	IL Cusp				<.0001*	*E*	OL A Bulge	IL Basis				<.0001*
*H*	OL E Stylus, basis	IL Stylus, term				<.0001*	*E*	OL A Bulge	IL Stylus, basis				<.0001*
*H*	OL E Stylus, basis	IL Cusp				<.0001*	*E*	OL B Basis	IL Stylus, basis				<.0001*
*H*	OL E Cusp	IL Stylus, term				<.0001*	*E*	OL B Bulge	IL Basis				<.0001*
*H*	OL B Bulge	IL Basis				<.0001*	*E*	OL B Bulge	IL Stylus, basis				<.0001*
*H*	OL C Bulge	IL Basis				<.0001*	*E*	OL C Basis	IL Stylus, basis				<.0001*
*H*	OL D Stylus, basis	IL Stylus, basis				<.0001*	*E*	OL C Bulge	IL Basis				<.0001*
*H*	OL A Stylus, term	IL Cusp				<.0001*	*E*	OL C Bulge	IL Stylus, basis				<.0001*
*H*	OL A Basis	IL Stylus, basis				<.0001*	*E*	OL D Basis	IL Stylus, basis				<.0001*
*H*	OL A Bulge	IL Basis				<.0001*	*E*	OL D Bulge	IL Basis				<.0001*
*H*	OL A Bulge	IL Stylus, basis				<.0001*	*E*	OL D Bulge	IL Stylus, basis				<.0001*
*H*	OL B Basis	IL Stylus, basis				<.0001*	*E*	OL D Stylus, basis	IL Stylus, basis				<.0001*
*H*	OL B Bulge	IL Stylus, basis				<.0001*	*E*	OL E Basis	IL Stylus, basis				<.0001*
*H*	OL C Basis	IL Stylus, basis				<.0001*	*E*	OL E Bulge	IL Basis				<.0001*
*H*	OL C Bulge	IL Stylus, basis				<.0001*	*E*	OL E Bulge	IL Stylus, basis				<.0001*
*H*	OL D Basis	IL Stylus, basis				<.0001*	*E*	OL E Stylus, basis	IL Stylus, basis				<.0001*
*H*	OL D Bulge	IL Basis				<.0001*	*E*	OL D Cusp	IL Stylus, term				<.0001*
*H*	OL D Bulge	IL Stylus, basis				<.0001*	*E*	OL B Stylus, term	IL Stylus, term				<.0001*
*H*	OL E Basis	IL Stylus, basis				<.0001*	*E*	OL A Stylus, term	IL Cusp				<.0001*
*H*	OL E Bulge	IL Basis				<.0001*	*E*	OL B Stylus, term	IL Cusp				<.0001*
*H*	OL E Bulge	IL Stylus, basis				<.0001*	*E*	OL C Stylus, term	IL Stylus, term				<.0001*
*H*	OL E Stylus, basis	IL Stylus, basis				<.0001*	*E*	OL C Stylus, term	IL Cusp				<.0001*
*H*	OL C Stylus, term	IL Stylus, term				<.0001*	*E*	OL D Stylus, term	IL Stylus, term				<.0001*
*H*	OL B Stylus, term	IL Cusp				<.0001*	*E*	OL D Stylus, term	IL Cusp				<.0001*
*H*	OL C Stylus, term	IL Cusp				<.0001*	*E*	OL D Cusp	IL Cusp				<.0001*
*H*	OL D Stylus, term	IL Stylus, term				<.0001*	*E*	OL E Stylus, term	IL Stylus, term				<.0001*
*H*	OL D Stylus, term	IL Cusp				<.0001*	*E*	OL E Stylus, term	IL Cusp				<.0001*
*H*	OL E Stylus, term	IL Stylus, term				<.0001*	*E*	OL E Cusp	IL Stylus, term				<.0001*
*H*	OL E Stylus, term	IL Cusp				<.0001*	*E*	OL E Cusp	IL Cusp				<.0001*
*H*	OL E Cusp	IL Cusp				<.0001*	*E*	OL D Stylus, term	IL Stylus, basis				<.0001*
*H*	OL E Stylus, term	IL Stylus, basis				<.0001*	*E*	OL E Stylus, term	IL Stylus, basis				<.0001*
*H*	IL Bulge	IL Basis				<.0001*	*E*	IL Bulge	IL Basis				<.0001*

*Note*: Orange *p*‐values = highly significant differences, red *p*‐values = significant differences, black = no significant differences.

Abbreviation: df, degrees of freedom; IL, inner lateral; OL, outer lateral; term, terminal.

## References

[ece310332-bib-0001] Anderson, P. S. L. (2018). Making a point: Shared mechanics underlying the diversity of biological puncture. The Journal of Experimental Biology, 221, jeb187294. 30446527 10.1242/jeb.187294

[ece310332-bib-0002] Barber, A. H. , Lu, D. , & Pugno, N. M. (2015). Extreme strength observed in limpet teeth. Journal of The Royal Society Interface, 12(105), 20141326. 25694539 10.1098/rsif.2014.1326 PMC4387522

[ece310332-bib-0003] Bendsøe, M. P. (1989). Optimal shape design as a material distribution problem. Structural Optimization, 1, 193–202.

[ece310332-bib-0004] Bendsøe, M. P. (1995). Optimization of structural topology. Shape and material. Springer.

[ece310332-bib-0005] Bendsøe, M. P. , & Kikuchi, N. (1988). Generating optimal topologies in structural design using a homogenization method. Computer Methods in Applied Mechanics and Engineering, 71, 197–224.

[ece310332-bib-0006] Berry, A. J. , & Thomson, D. R. (1990). Changing prey size preferences in the annual cycle of *Retusa obtusa* (Montagu) (Opisthobranchia) feeding on *Hydrobia ulvae* (Pennant) (Prosobranchia). Journal of Experimental Marine Biology and Ecology, 141, 145–158.

[ece310332-bib-0007] Beutel, R. G. , Richter, A. , Keller, R. A. , Garcia, F. H. , Matsumura, Y. , Economo, E. P. , & Gorb, S. N. (2020). Distal leg structures of the Aculeata (Hymenoptera): A comparative evolutionary study of *Sceliphron* (Sphecidae) and *Formica* (Formicidae). Journal of Morphology, 281, 737–753. 32364646 10.1002/jmor.21133

[ece310332-bib-0008] Brooker, L. R. , Lee, A. P. , Macey, D. J. , van Bronswijk, W. , & Webb, J. (2003). Multiple‐front iron‐mineralisation in chiton teeth (*Acanthopleura echinata*: Mollusca: Polyplacophora). Marine Biology, 142, 447–454.

[ece310332-bib-0009] Brooker, L. R. , & Shaw, J. A. (2012). The chiton radula: A unique model for biomineralization studies. In J. Seto (Ed.), Advanced topics in biomineralization (pp. 65–84). Intech Open.

[ece310332-bib-0010] Cedhagen, T. (1996). Foraminiferans as food for cephalaspideans (Gastropoda: Opisthobranchia), with notes on secondary tests around calcareous foraminiferans. Special Publication. Phuket Marine Biological Center, 16, 279–290.

[ece310332-bib-0011] Chester, C. M. (1993). Comparative feeding biology of *Acteocina canaliculata* (Say, 1826) and *Haminoea solitaria* (Say, 1822) (Opisthobranchia: Cephalaspidea). American Malacological Bulletin, 10(1), 93–101.

[ece310332-bib-0012] DeLaHoz, M. V. , Sardà, F. , Coll, M. , Sáez, R. , Mechó, A. , Oliva, F. , Ballesteros, M. , & Palomera, I. (2018). Biodiversity patterns of megabenthic non‐crustacean invertebrates from an exploited ecosystem of the northwestern Mediterranean Sea. Regional Studies in Marine Science, 19, 47–68.

[ece310332-bib-0013] Dumont, E. R. , Grosse, I. R. , & Slater, G. J. (2009). Requirements for comparing the performance of finite element models of biological structures. Journal of Theoretical Biology, 256, 96–103. 18834892 10.1016/j.jtbi.2008.08.017

[ece310332-bib-0014] Eilertsen, M. H. , & Malaquias, M. A. E. (2013). Unique digestive system, trophic specialization, and diversification in the deep‐sea gastropod genus *Scaphander* . Biological Journal of the Linnean Society, 109(3), 512–525.

[ece310332-bib-0015] Evans, L. A. , Macey, D. J. , & Webb, J. (1990). Characterization and structural organization of the organic matrix of radula teeth of the chiton *Acanthopleura hirtosa* . Philosophical Transactions of the Royal Society London B, 329, 87–96.

[ece310332-bib-0016] Evans, L. A. , Macey, D. J. , & Webb, J. (1994). Matrix heterogeneity in the radular teeth of the chiton *Acanthopleura hirtosa* . Acta Zoologica, 75, 75–79.

[ece310332-bib-0017] Faivre, D. , & Ukmar‐Godec, T. (2015). From bacteria to mollusks: The principles underlying the biomineralization of iron oxide materials. Angewandte Chemie, International Edition, 54(16), 4728–4747. 25851816 10.1002/anie.201408900

[ece310332-bib-0018] Freeman, P. W. , & Lemen, C. A. (2007). The trade‐off between tooth strength and tooth penetration: Predicting optimal shape of canine teeth. Journal of Zoology, 273, 273–280.

[ece310332-bib-0019] Friedrich, F. , & Kubiak, M. (2018). Comparative anatomy of pupal tarsi in caddisflies (Insecta: Trichoptera) with focus on the claw system. Zoomorphology, 137, 305–314.

[ece310332-bib-0020] Gorb, S. N. , & Krings, W. (2021). Mechanical property gradients of taenioglossan radular teeth are associated with specific function and ecological niche in Paludomidae (Gastropoda: Mollusca). Acta Biomaterialia, 134(15), 513–530. 34329785 10.1016/j.actbio.2021.07.057

[ece310332-bib-0021] Gordon, L. , & Joester, D. (2011). Nanoscale chemical tomography of buried organic–inorganic interfaces in the chiton tooth. Nature, 469, 194–198. 21228873 10.1038/nature09686

[ece310332-bib-0022] Grunenfelder, L. K. , de Obaldia, E. E. , Wang, Q. , Li, D. , Weden, B. , Salinas, C. , Wuhrer, R. , Zavattieri, P. , & Kisailus, D. (2014). Biomineralization: Stress and damage mitigation from oriented nanostructures within the radular teeth of *Cryptochiton stelleri* . Advanced Functional Materials, 24(39), 6093–6104.

[ece310332-bib-0023] Guralnick, R. , & Smith, K. (1999). Historical and biomechanical analysis of integration and dissociation in molluscan feeding, with special emphasis on the true limpets (Patellogastropoda: Gastropoda). Journal of Morphology, 241, 175–195. 10420163 10.1002/(SICI)1097-4687(199908)241:2<175::AID-JMOR7>3.0.CO;2-0

[ece310332-bib-0024] Han, Y. , Liu, C. , Zhou, D. , Li, F. , Wang, Y. , & Han, X. (2011). Magnetic and structural properties of magnetite in radular teeth of chiton *Acanthochiton rubrolinestus* . Bioelectromagnetics, 32, 226–233. 21365666 10.1002/bem.20636

[ece310332-bib-0025] Hawkins, S. J. , Watson, D. C. , Hill, A. S. , Harding, S. P. , Kyriakides, M. A. , Hutchinson, S. , & Norton, T. A. (1989). A comparison of feeding mechanisms in microphagous, herbivorous, intertidal, prosobranchs in relation to resource partitioning. Journal of Molluscan Studies, 55(2), 151–165.

[ece310332-bib-0026] Herrera, S. A. , Grunenfelder, L. , Escobar, E. , Wang, Q. , Salinas, C. , Yaraghi, N. , Geiger, J. , Wuhrer, R. , Zavattieri, P. , & Kisailus, D. (2015). Stylus support structure and function of radular teeth in *Cryptochiton stelleri*. 20th International Conference on Composite Materials, Copenhagen, 19–24th July.

[ece310332-bib-0027] Hickman, C. S. (1980). Gastropod radulae and the assessment of form in evolutionary paleontology. Paleobiology, 6, 276–294.

[ece310332-bib-0028] Hickman, C. S. (1984). Implications of radular tooth‐row functional‐integration for archaeogastropod systematics. Malacologia, 25, 143–160.

[ece310332-bib-0029] Hurst, A. (1965). Studies on the structure and function of the feeding apparatus of *Philineaperta* with comparative consideration of some other opisthobranchs. Malacologia, 2, 281–347.

[ece310332-bib-0030] Joester, D. , & Brooker, L. R. (2016). The chiton radula: A model system for versatile use of iron oxides. In D. Faivre (Ed.), Iron oxides: From nature to applications (pp. 177–205). Wiley‐VCH.

[ece310332-bib-0031] Kim, K. S. , Macey, D. J. , Webb, J. , & Mann, S. (1989). Iron mineralisation in the radula teeth of the chiton *Acanthopleura hirtosa* . Proceedings of the Royal Society B, 237, 335–346.

[ece310332-bib-0032] Kirschvink, J. L. , & Lowenstam, H. A. (1979). Mineralization and magnetization of chiton teeth: Paleomagnetic, sedimentalogic and biologic implications of organic magnetite. Earth and Planetary Science Letters, 44(2), 193–204.

[ece310332-bib-0033] Kisailus, D. , & Nemoto, M. (2018). Structural and proteomic analyses of iron oxide biomineralization in chiton teeth. In T. Matsunaga , T. Tanaka , & D. Kisailus (Eds.), Biological magnetic materials and applications (pp. 53–73). Springer.

[ece310332-bib-0034] Krings, W. (2020). Trophic specialization of paludomid gastropods from 'ancient' Lake Tanganyika reflected by radular tooth morphologies and material properties. Dissertation, University of Hamburg.

[ece310332-bib-0035] Krings, W. , Brütt, J.‐O. , & Gorb, S. N. (2022a). Micro‐cracks and micro‐fractures reveal radular tooth architecture and its functional significance in the paludomid gastropod *Lavigeria grandis* . Philosophical Transactions of the Royal Society A, 380, 20210335. 10.1098/rsta.2021.0335 35909353

[ece310332-bib-0036] Krings, W. , Brütt, J.‐O. , & Gorb, S. N. (2022b). Mechanical properties, degree of sclerotisation and elemental composition of the gastric mill in the red swamp crayfish *Procambarus clarkii* (Decapoda, Crustacea). Scientific Reports, 12, 17799. 36274188 10.1038/s41598-022-22724-w PMC9588795

[ece310332-bib-0037] Krings, W. , Brütt, J.‐O. , & Gorb, S. N. (2022c). Ontogeny of the elemental composition and the biomechanics of radular teeth in the chiton *Lepidochitona cinerea* . Frontiers in Zoology, 19, 19. 35690761 10.1186/s12983-022-00465-w PMC9188181

[ece310332-bib-0038] Krings, W. , Brütt, J.‐O. , & Gorb, S. N. (2022d). Material gradients in gastropod radulae and their biomechanical significance: A combined approach on the paludomid *Lavigeria grandis* . The Science of Nature, 109, 52. 10.1007/s00114-022-01822-9 PMC9630255 36322292

[ece310332-bib-0039] Krings, W. , Brütt, J.‐O. , Gorb, S. N. , & Glaubrecht, M. (2020). Tightening it up: Diversity of the chitin anchorage of radular‐teeth in paludomid freshwater‐gastropods. Malacologia, 63, 77–94.

[ece310332-bib-0040] Krings, W. , & Gorb, S. N. (2021). Radula packing and storage facilitated by tooth morphology in selected taenioglossan Gastropoda. Journal of Molluscan Studies, 87, eyab007.

[ece310332-bib-0041] Krings, W. , & Gorb, S. N. (2023a). Resistance to abrasive particles in soft radular teeth (Paludomidae, Gastropoda, Mollusca). Invertebrate Biology. In press.

[ece310332-bib-0042] Krings, W. , & Gorb, S. N. (2023b). Mechanical properties of larval mouthparts of the antlion *Euroleon nostras* (Neuroptera: Myrmeleontidae) and their correlation with cuticular material composition. Zoomorphology.

[ece310332-bib-0043] Krings, W. , Karabacak, H. , & Gorb, S. N. (2021). From the knitting shop: The first physical and dynamic model of the taenioglossan radula (Mollusca: Gastropoda) aids in unravelling functional principles of the radular morphology. Journal of the Royal Society Interface, 18(182), 20210377. 34520692 10.1098/rsif.2021.0377 PMC8440039

[ece310332-bib-0044] Krings, W. , Kovalev, A. , Glaubrecht, M. , & Gorb, S. N. (2019). Differences in the young modulus and hardness reflect different functions of teeth within the taenioglossan radula of gastropods. Zoology, 137, 125713. 31706151 10.1016/j.zool.2019.125713

[ece310332-bib-0045] Krings, W. , Kovalev, A. , & Gorb, S. N. (2021b). Influence of water content on mechanical behaviour of gastropod taenioglossan radulae. Proceedings of the Royal Society B, 288, 20203173. 33653134 10.1098/rspb.2020.3173 PMC7935061

[ece310332-bib-0046] Krings, W. , Kovalev, A. , & Gorb, S. N. (2021c). Collective effect of damage prevention in taenioglossan radular teeth is related to the ecological niche in Paludomidae (Gastropoda: Cerithioidea). Acta Biomaterialia, 135, 458–172. 34358696 10.1016/j.actbio.2021.07.073

[ece310332-bib-0047] Krings, W. , Marcé‐Nogué, J. , & Gorb, S. N. (2021). Finite element analysis relating shape, material properties, and dimensions of taenioglossan radular teeth with trophic specialisations in Paludomidae (Gastropoda). Scientific Reports, 11, 22775. 34815469 10.1038/s41598-021-02102-8 PMC8611077

[ece310332-bib-0048] Krings, W. , Marcé‐Nogué, J. , Karabacak, H. , Glaubrecht, M. , & Gorb, S. N. (2020). Finite element analysis of individual taenioglossan radular teeth (Mollusca). Acta Biomaterialia, 115, 317–332. 32853812 10.1016/j.actbio.2020.08.034

[ece310332-bib-0049] Krings, W. , Wägele, H. , Neumann, C. , & Gorb, S. N. (2023). Coping with abrasive food: Diverging composition of radular teeth in two Porifera‐consuming nudibranch species (Mollusca, Gastropoda). Journal of the Royal Society Interface, 20, 20220927. 37221862 10.1098/rsif.2022.0927 PMC10206459

[ece310332-bib-0050] Lee, A. P. , Brooker, L. R. , Macey, D. J. , Webb, J. , & van Bronswijk, W. (2003). A new biomineral identified in the cores of teeth from the chiton *Plaxiphora albida* . Journal of Biological Inorganic Chemistry, 8, 256–262. 12589561 10.1007/s00775-002-0410-y

[ece310332-bib-0051] Lee, A. P. , Brooker, L. R. , van Bronswijk, W. , Macey, D. J. , & Webb, J. (2003). Contribution of Raman spectroscopy to identification of biominerals present in teeth of *Acanthopleura rehderi*, *Acanthopleura curtisiana*, and *Onithochiton quercinus* . Biopolymers, 72, 299–301. 12833485 10.1002/bip.10380

[ece310332-bib-0052] Lehnert, M. S. , Johnson, D. D. , Wu, J. , Sun, Y. , Fonseca, R. J. , Michels, J. , Shell, J. S. , & Reiter, K. E. (2021). Physical adaptations of butterfly proboscises enable feeding from narrow floral tubes. Functional Ecology, 35, 1925–1937.

[ece310332-bib-0053] Lowenstam, H. A. , & Weiner, S. (1989). Mollusca. In H. A. Lowenstam & S. Weiner (Eds.), On biomineralization (pp. 88–305). Oxford University Press.

[ece310332-bib-0054] Lu, D. , & Barber, A. H. (2012). Optimized nanoscale composite behaviour in limpet teeth. Journal of the Royal Society Interface, 9(71), 1318–1324. 22158842 10.1098/rsif.2011.0688 PMC3350734

[ece310332-bib-0055] Mackenstedt, U. , & Märkel, K. (1987). Experimental and comparative morphology of radula renewal in pulmonates (Mollusca, Gastropoda). Zoomorphology, 107, 209–239.

[ece310332-bib-0056] Malaquias, M. , Condinho, S. , Cervera, J. , & Sprung, M. (2004). Diet and feeding biology of *Haminoea orbygniana* (Mollusca: Gastropoda: Cephalaspidea). Journal of the Marine Biological Association of the United Kingdom, 84(4), 767–772.

[ece310332-bib-0057] Matsumura, Y. , Kovalev, A. , & Gorb, S. N. (2021). Mechanical properties of a female reproductive tract of a beetle and implications for penile penetration. Proceedings of the Royal Society B, 288, 20211125. 34229492 10.1098/rspb.2021.1125 PMC8261216

[ece310332-bib-0058] Michels, J. , & Gorb, S. N. (2012). Detailed three‐dimensional visualization of resilin in the exoskeleton of arthropods using confocal laser scanning microscopy. Journal of Microscopy, 245(1), 1–16. 22142031 10.1111/j.1365-2818.2011.03523.x

[ece310332-bib-0059] Michels, J. , & Gorb, S. N. (2015). Mandibular gnathobases of marine planktonic copepods—Structural and mechanical challenges for diatom frustules in evolution of lightweight structures. In C. Hamm (Ed.), Biologically‐inspired systems (pp. 59–73). Springer.

[ece310332-bib-0060] Michels, J. , Vogt, J. , & Gorb, S. N. (2012). Tools for crushing diatoms—Opal teeth in copepods feature a rubber‐like bearing composed of resilin. Scientific Reports, 2, 465. 22745896 10.1038/srep00465 PMC3385419

[ece310332-bib-0061] Montroni, D. , Zhang, X. , Leonard, J. , Kaya, M. , Amemiya, C. , Falini, G. , & Rolandi, M. (2019). Structural characterization of the buccal mass of *Ariolimax californicus* (Gastropoda; Stylommatophora). PLoS One, 14, e0212249. 31390363 10.1371/journal.pone.0212249 PMC6685607

[ece310332-bib-0062] Morris, T. E. , & Hickman, C. S. (1981). A method for artificially protruding gastropod radulae and a new model of radula function. Veliger, 24, 85–89.

[ece310332-bib-0063] Morrow, C. , & Cárdenas, P. (2015). Proposal for a revised classification of the Demospongiae (Porifera). Frontiers in Zoology, 12, 7. 25901176 10.1186/s12983-015-0099-8 PMC4404696

[ece310332-bib-0064] Ong, E. , Hallas, J. M. , & Gosliner, T. M. (2017). Like a bat out of heaven: The phylogeny and diversity of the bat‐winged slugs (Heterobranchia: Gastropteridae). Zoological Journal of the Linnean Society, 180(4), 755–789.

[ece310332-bib-0065] Padilla, D. K. (2003). Form and function of radular teeth of herbivorous molluscs: Focus on the future. American Malacological Bulletin, 18(1–2), 163–168.

[ece310332-bib-0066] Peisker, H. , Michels, J. , & Gorb, S. N. (2013). Evidence for a material gradient in the adhesive tarsal setae of the ladybird beetle *Coccinella septempunctata* . Nature Communications, 4, 1661. 10.1038/ncomms2576 23552076

[ece310332-bib-0067] Pohl, A. , Herrera, S. A. , Restrepo, D. , Negishi, R. , Jung, J.‐Y. , Salinas, C. , Wuhrer, R. , Yoshino, T. , McKittrick, J. , Arakaki, A. , Nemoto, M. , Zavattieri, P. , & Kisailus, D. (2020). Radular stylus of *Cryptochiton stelleri*: A multifunctional lightweight and flexible fiber‐reinforced composite. Journal of the Mechanical Behavior of Biomedical Materials, 111, 103991. 32823075 10.1016/j.jmbbm.2020.103991

[ece310332-bib-0068] Rudman, W. B. (1972a). Structure and functioning of the gut in the Bullomorpha (Opisthobranchia). Part 3. Philinidae. Journal of Natural History, 6, 459–474.

[ece310332-bib-0069] Rudman, W. B. (1972b). The genus *Philine* (Opisthobranchia, Gastropoda). Proceedings of the Malacological Society of London, 40, 171–187.

[ece310332-bib-0070] Rudman, W. B. (1978). A new species and genus of the Aglajidae and the evolution of the philinacean opisthobranch molluscs. Zoological Journal of the Linnean Society, 62(1), 89–107.

[ece310332-bib-0071] Runham, N. W. (1963). A study of the replacement mechanism of the pulmonate radula. Journal of Cell Science, 3(66), 271–277.

[ece310332-bib-0072] Runham, N. W. , & Isarankura, K. (1966). Studies on radula replacement. Malacologia, 5, 73.

[ece310332-bib-0073] Saunders, M. , Kong, C. , Shaw, J. A. , & Clode, P. L. (2011). Matrix‐mediated biomineralization in marine mollusks: A combined transmission electron microscopy and focused ion beam approach. Microscopy and Microanalysis, 17, 220–225. 21371372 10.1017/S1431927610094547

[ece310332-bib-0074] Saunders, M. , Kong, C. , Shaw, J. A. , Macey, D. J. , & Clode, P. L. (2009). Characterization of biominerals in the radula teeth of the chiton, *Acanthopleura hirtosa* . Journal of Structural Biology, 167, 55–61. 19292994 10.1016/j.jsb.2009.03.003

[ece310332-bib-0075] Shaw, J. A. , Macey, D. J. , Brooker, L. R. , & Clode, P. L. (2010). Tooth use and wear in three iron‐biomineralizing mollusc species. The Biological Bulletin, 218, 132–144. 20413790 10.1086/BBLv218n2p132

[ece310332-bib-0076] Shaw, J. A. , Macey, D. J. , Brooker, L. R. , Stockdale, E. J. , Saunders, M. , & Clode, P. L. (2009a). The chiton stylus canal: An element delivery pathway for tooth cusp biomineralization. Journal of Morphology, 270, 588–600. 19107814 10.1002/jmor.10705

[ece310332-bib-0077] Shaw, J. A. , Macey, D. J. , Brooker, L. R. , Stockdale, E. J. , Saunders, M. , & Clode, P. L. (2009b). Ultrastructure of the epithelial cells associated with tooth biomineralization in the chiton *Acanthopleura hirtosa* . Microscopy and Microanalysis, 15(2), 154–165. 19284897 10.1017/S1431927609090230

[ece310332-bib-0078] Shepelenko, M. , Brumfeld, V. , Cohen, S. R. , Klein, E. , Lubinevsky, H. , Addadi, L. , & Weiner, S. (2015). The gizzard plates in the Cephalaspidean gastropod *Philine quadripartita*: Analysis of structure and function. Quaternary International, 390, 4–14.

[ece310332-bib-0079] Shonman, D. , & Nybakken, J. W. (1978). Food preferences, food availability and food re‐source partioning in two sympatric species of cephalaspidean opisthobranchs. The Veliger, 21(1), 120–126.

[ece310332-bib-0080] Solem, A. (1972). Malacological applications of scanning electron microscopy II. Radular structure and functioning. Veliger, 14, 327–336.

[ece310332-bib-0081] Solem, A. (1974). The shell makers: Introducing mollusks. Jon Wiley & Sons.

[ece310332-bib-0082] Stegbauer, L. , Smeets, P. J. M. , Free, R. , Wallace, S. G. , Hersam, M. C. , Alp, E. E. , & Joester, D. (2021). Persistent polyamorphism in the chiton tooth: From a new biomineral to inks for additive manufacturing. Proceedings of the National Academy of Sciences of the United States of America, 118, e2020160118. 34088834 10.1073/pnas.2020160118 PMC8202020

[ece310332-bib-0083] Steneck, R. S. , & Watling, L. (1982). Feeding capabilities and limitation of herbivorous molluscs: A functional group approach. Marine Biology, 68, 299–319.

[ece310332-bib-0084] Thompson, T. E. (1976). Biology of opisthobranch Molluscs. The Ray Society 206 pp.

[ece310332-bib-0085] Thompson, T. E. (1988). Molluscs: Benthic opisthobranchs (Mollusca: Gastropoda). Synopses of the British Fauna (New Series), No. 8 (2nd ed.). E.J. Brill & Dr. W. Backhuys 356 pp.

[ece310332-bib-0086] Ukmar‐Godec, T. (2016). Mineralization of goethite in limpet radular teeth. In D. Faivre (Ed.), Iron oxides: From nature to applications (pp. 207–224). Wiley‐VCH.

[ece310332-bib-0087] Ukmar‐Godec, T. , Bertinetti, L. , Dunlop, J. W. C. , Godec, A. , Grabiger, M. A. , Masic, A. , Nguyen, H. , Zlotnikov, I. , Zaslansky, P. , & Faivre, D. (2017). Materials nanoarchitecturing via cation‐mediated protein assembly: Making limpet teeth without mineral. Advanced Materials, 29(27), 1701171. 10.1002/adma.201701171 28485089

[ece310332-bib-0088] Ukmar‐Godec, T. , Kapun, G. , Zaslansky, P. , & Faivre, D. (2015). The giant keyhole limpet radular teeth: A naturally‐grown harvest machine. Journal of Structural Biology, 192, 392–402. 26433029 10.1016/j.jsb.2015.09.021 PMC4658332

[ece310332-bib-0089] van der Wal, P. (1989). Structural and material design of mature mineralized radula teeth of *Patella vulgata* (Gastropoda). Journal of Ultrastructure and Molecular Structure Research, 102, 147–161.

[ece310332-bib-0090] van der Wal, P. , Giesen, H. J. , & Videler, J. J. (1999). Radular teeth as models for the improvement of industrial cutting devices. Materials Science and Engineering: C, 7(2), 129–142.

[ece310332-bib-0091] van der Wal, P. , Videler, J. J. , Havinga, P. , & Pel, R. (1989). Architecture and chemical composition of the magnetite‐bearing layer in the radula teeth of *Chiton olivaceus* (Polyplacophora). In R. E. Crick (Ed.), Origin, evolution, and modern aspects of biomineralization in plants and animals (pp. 153–166). Springer.

[ece310332-bib-0092] Vortsepneva, E. , Mikhlina, A. , & Kantor, Y. (2022). Main patterns of radula formation and ontogeny in Gastropoda. Journal of Morphology, 284, e21538. 10.1002/jmor.21538 36426387

[ece310332-bib-0093] Wang, C. , Li, Q. Y. , Wang, S. N. , Qu, S. X. , & Wang, X. X. (2014). Microstructure and self‐sharpening of the magnetite cap in chiton tooth. Materials Science and Engineering: C, 37, 1–8. 24582215 10.1016/j.msec.2013.12.029

[ece310332-bib-0094] Wang, Q. , Nemoto, M. , Li, D. , Weaver, J. C. , Weden, B. , Stegemeier, J. , Bozhilov, K. N. , Wood, L. R. , Milliron, G. W. , Kim, C. S. , DiMasi, E. , & Kisailus, D. (2013). Phase transformations and structural developments in the radular teeth of *Cryptochiton stelleri* . Advanced Functional Materials, 23, 2908–2917.

[ece310332-bib-0095] Wealthall, R. J. , Brooker, L. R. , Macey, D. J. , & Griffin, B. J. (2005). Fine structure of the mineralized teeth of the chiton *Acanthopleura echinata* (Mollusca: Polyplacophora). Journal of Morphology, 265, 165–175. 15959908 10.1002/jmor.10348

[ece310332-bib-0096] Weaver, J. C. , Wang, Q. , Miserez, A. , Tantuccio, A. , Stromberg, R. , Bozhilov, K. N. , Maxwell, P. , Nay, R. , Heier, S. T. , & DiMasi, E. (2010). Analysis of an ultra hard magnetic biomineral in chiton radular teeth. Materials Today, 13(1–2), 42–52.

